# Gene-specific machine learning model EpiPred identifies likely pathogenic variants in the epilepsy-related gene STXBP1

**DOI:** 10.1172/JCI207160

**Published:** 2026-09-17

**Authors:** Jeffrey D. Calhoun, Chengbing Wang, Carina G. Biar, Jonathan R. Gunti, John S. Lee, Aaron M. Geller, Jung H. Hong, Santiago Schnell, Louis T. Dang, Yu Wang, Jack M. Parent, Lori L. Isom, Michael D. Uhler, Heather C. Mefford, M. Elizabeth Ross, Vanessa Aguiar-Pulido, Gemma L. Carvill

**Affiliations:** 1 Ken and Ruth Davee Department of Neurology, Northwestern Feinberg School of Medicine, Chicago, Illinois, USA.; 2 Center for Neurogenetics, Feil Family Brain and Mind Research Institute, Weill Cornell Medicine, New York, New York, USA.; 3 Northwestern IT Research Computing and Data Services, Evanston, Illinois, USA.; 4 Departments of Mathematics, Biochemistry & Cell Biology and Biomedical Data Science, Dartmouth College, Hanover, New Hampshire, USA.; 5 Department of Pediatrics,; 6 Department of Neurology,; 7 Department of Pharmacology, and; 8 Department of Biological Chemistry and Molecular Neuroscience Institute, University of Michigan, Ann Arbor, Michigan, USA.; 9 Center for Pediatric Neurological Disease Research, St. Jude Children’s Research Hospital, Memphis, Tennessee, USA.; 10 Department of Computer Science, University of Miami, Miami, Florida, USA.; 11 Department of Pharmacology, Northwestern Feinberg School of Medicine, Chicago, Illinois, USA.

**Keywords:** Genetics, Neuroscience, Epilepsy, Genetic diseases, Genetic variation

## Abstract

*STXBP1* variants are a frequent cause of early onset developmental and epileptic encephalopathies and related neurodevelopmental disorders, but the clinical interpretation of these variants remains a major challenge. Most reported *STXBP1* missense variants are classified as variants of uncertain significance (VUS), complicating diagnosis, counseling, and patient eligibility for precision therapies. Here, we developed EpiPred, a gene-specific machine learning classifier that predicts the pathogenicity of *STXBP1* missense variants and tests these predictions using empirical evidence from well-established cellular assays. Trained on a curated set of pathogenic and benign variants, EpiPred outperformed global prediction tools in accuracy, sensitivity, and specificity. We validated the model’s predictions using variant effect assays that measure protein abundance, solubility, stability, and interaction with the SNARE complex partner syntaxin 1. These biochemical readouts aligned closely with model outputs and enabled reclassification of several possibly misdiagnosed variants, which warrant further validation and clinical reevaluation. We deployed EpiPred in an interactive web application that allows clinicians, researchers, and patients to explore predictions for all possible STXBP1 missense variants. By identifying likely pathogenic *STXBP1* variants, including those that may respond to emerging therapies, such as protein stabilizers. By coupling gene-calibrated machine learning with orthogonal variant-effect assays and public deployment, EpiPred provides a transferable framework for VUS resolution, trial enrichment, and precision diagnosis across clinically actionable Mendelian disease genes.

## Abstract 2

New tool, EpiPred, uses machine learning plus cellular modeling to resolve uncertain STXBP1 gene variants, helping diagnose epilepsy patients and enable precision therapies.

## Introduction

Pathogenic loss-of-function variants in the gene *STXBP1* are one of the most common monogenic causes of epilepsy and related neurodevelopmental disorders (NDDs). Clinically, disease-causing *STXBP1* variants were first described in the Developmental and Epileptic Encephalopathies (DEEs) (1), but there has been a widening of the phenotypic spectrum to NDDs more broadly. However, epilepsy remains a prominent feature, present in 95% of patients (2). Two metaanalyses revealed *STXBP1* as the 5th most common epilepsy-related gene and the 6th most commonly mutated gene relative to its size of all de novo variant containing genes (3, 4). *STXBP1* has been included in gene panels since approximately 2011, and, as such, there are hundreds of variants of uncertain significance (VUS). For example, in a cohort of approximately 10,000 individuals sequenced for *STXBP1,* an almost equal number of pathogenic variants (0.48%) were identified as the number of individuals reported with an *STXBP1* VUS (0.3%) (5). When we began this study, there were 150 missense VUS listed in ClinVar, the database for deposition of clinically relevant genetic variants. This number has subsequently risen to 222, an increase of 33% in just a few years, highlighting the need for VUS resolution. The emerging challenge of VUS is not limited to *STXBP1,* as there are currently over 1.5 million VUS in ClinVar accounting for over 90% of all variants in this collation (6). Moreover, roughly half of all clinically reported variants are VUS (7). Here, we built EpiPred, a supervised machine learning model designed to classify STXBP1 missense variants using both computational and biochemical characterization. This study forms part of the Epilepsy Multiplatform Variant Prediction (EpiMVP) study, an NIH-funded, multicenter collaborative effort to resolve epilepsy-related VUS through the integration of computational modeling and cellular and animal-based experimental assays.

The majority of pathogenic *STXBP1* variants are truncations that arise de novo, consistent with a disease mechanism of haploinsufficiency. The 100 STXBP1 pathogenic missense variants are located throughout the protein. This lack of clustering into discrete functional domains limits VUS resolution strategies that have been shown to be effective in other epilepsy-related genes, such as the voltage-gated ion channels, where variants tend to aggregate in the pore-forming regions (8). Thus, we must leverage other key features of pathogenic missense variants to discriminate between pathogenic and benign *STXBP1* variants. STXBP1, also known as UNC18/MUNC18, together with STX1, synaptobrevin and SNAP25 form the SNARE complex (9). This complex is a key component of neuronal function, as it facilitates fusion of the synaptic vesicle with the plasma membrane allowing neurotransmitter release into the synapse.

Fortunately, there is an increasing understanding of STXBP1 missense pathogenic variant effects. This includes reduced abundance of mutant STXBP1 proteins in HEK293 cells, *Stxbp1* null mouse neurons (10, 11), induced pluripotent stem cells (iPSCs) (12), as well as engineered *C*. *elegans* models (13–15). Pathogenic missense variants also lead to reduced interaction with SNARE partner, STX1, and interactor Doc1 (10, 11). Finally, there is some evidence for protein aggregation (11, 15). Together, these studies converge on a common STXBP1 missense variant effect phenotype characterized by reduced protein abundance, stability, and solubility, along with disrupted STX1 interaction; features that enable the experimental resolution of missense VUS.

In addition to experimental assessment of variant effect, global and gene-specific variant effect predictors (VEPs) are increasingly used to predict variant effect. Global, or genome-wide, VEPs, such as REVEL, AlphaMissense, ClinPred, VARITY, VESM, gMVP are often used as supporting evidence when determining variant pathogenicity, per the American College of Medical Genetics (ACMG) guidelines (16–21). These global predictors are the most common and generalize reasonably well due to the large datasets available for training. However, there are increasing efforts to develop both disease (cancer, cardiac conditions) (22, 23) and gene-specific (BRCA1/2) classifiers (24–27). These gene-specific classifiers are necessarily narrower in scope, but they can exploit gene-level biology, curated clinical labels, and mechanistic functional data to improve clinically relevant calibration. This includes predicting STXBP1-related disorder (PRESR), a gene-specific predictor benchmarked primarily against pathogenic variants alone (13).

Here, we generated an *STXBP1*-specific VEP, EpiPred (Epilepsy Prediction), using a supervised machine learning approach. We benchmarked EpiPred-*STXBP1* against commonly used global VEPs and tested our predictions using known readouts of *STXBP1* variant effect, including reduced protein abundance, stability, and solubility, and disrupted STX1 interaction (Figure 1A).

Collectively, EpiPred-STXBP1 exhibited improved performance over global VEPs and had high concordance with experimental analyses across 20 variants, including equal distribution of known positive (pathogenic) and negative (benign) controls to better validate the dynamic range of predictive power. We also generated predictions for every possible missense variant in *STXBP1* and demonstrated that the core of the STXBP1 protein is susceptible to perturbation by missense variants and likely underpins reduced abundance of STXBP1. Importantly, the reduced abundance of STXBP1 can be rescued in both animal and cellular models using 4-phenylbutyrate (PBA), an FDA-approved medication commonly used to lower serum ammonia levels in individuals with urea cycle disorders (15). A small clinical trial of PBA in individuals with *STXBP1*-related neurodevelopmental disorders suggests that the treatment may reduce seizure frequency and improve EEG abnormalities (28). Thus, collectively, our study highlights the power of integrating computational and experimental characterization of STXBP1 missense variants to inform precision diagnostics and empower precision clinical care, and suggests similar gene-specific calibration of VEPs, or gene-specific models can improve in silico variant prediction broadly in genetic conditions.

## Results

### EpiPred-STXBP1 accurately distinguishes between pathogenic and benign variants.

We used our curated dataset of STXBP1 missense variants, which includes 80 BLB and 96 PLP variants annotated with 42 distinct features, including other VEPs, to develop the single-gene ensemble classifier, EpiPred-STXBP1 (Supplemental Tables 1–3; supplemental material available online with this article; https://doi.org/10.1172/JCI207160DS1). We excluded any global VEPs that were trained on ClinVar data, given that this was a primary source for variants used to train EpiPred. We built a random forest model, which performed as a robust gene-specific classifier for our Truth Set (TS) (Supplemental Figure 1).

Despite being trained on a substantially smaller dataset, EpiPred outperformed all global VEPs with an AUROC value of 98%, with the next 2 closest competitors having AUROC values of 97% (Varity) and 96% (ClinPred) (Figure 1B and Supplemental Table 4). Indeed, EpiPred outperformed the global VEPs and the *STXBP1*-specific tool, PRESR, on sensitivity (EpiPred: 96%; PRESR: 90%), specificity (EpiPred: 96%; PRESR: 93%), and accuracy (EpiPred: 96%; PRESR: 92%) (Supplemental Table 4 and Supplemental Figure 2). Several global VEPs that were not trained on ClinVar data, including AlphaMissense and VEST4, were prevalent features used in EpiPred, as assessed by SHAP, a game theory–based method to reveal feature impact on model decisions (29) (Supplemental Figure 3). Other features that impact model predictions range from evolutionary conservation (PhyloP; 470way mammalian), residue solvent accessibility (RSA), and in silico prediction of protein stability (MAESTRO). These latter features strongly suggested that stability may be a key defining feature of *STXBP1* PLP missense variants, aligning with the decreased abundance and stability observed for missense variants in animal and cell models (12–15).

We used this model to score all variants with a Probability of PLP (EpiPred-P^PLP^ score) (Figure 1, C and D, and Supplemental Table 1). Using a Gaussian mixture model, we defined the PLP threshold as an EpiPred-P^PLP^ score greater than 0.871 and the BLB threshold as an EpiPred-P^PLP^ score less than 0.625, with variants between these thresholds classified as ‘neutral’ (Figure 1C).

In general, gnomAD variants present greater than thrice in the dataset had EpiPred-P^PLP^ scores below the PLP threshold (77/80 – 96% accuracy). The 3 exceptions were Phe153Ser and Arg536His, both predicted PLP and Ala553Thr in the ambiguous range (Supplemental Table 1 and Supplemental Figure 4). In the PLP TS, 81% (78/96) of variants exceeded the EpiPred PLP threshold, with 9 variants in the ambiguous range and the remaining 10 PLP variants more likely to be benign by EpiPred (Supplemental Table 1 and Supplemental Figure 4). VUS (*n* = 150) values were scored broadly across the range, with 23% (35/150) in the PLP range and 55% (83/150) in the BLB range (Figure 1, C and D, and Supplemental Table 2). We mapped these conflicting variants (10 PLP, 5 BLB) onto the 3D structure of the protein and assessed the SHAP outputs for each variant, but we were unable to detect any common features that could consistently explain this misclassification (Supplemental Figures 4 and 5).

### Missense variant effect aligns with EpiPred predictions.

We selected 20 variants for experimental validation of EpiPred performance in a cellular system, including 10 BLB, 5 PLP, and 5 VUS. Of these VUS, 3 (p.Ala202Val, p.Trp288Leu, and p.Ile576Thr) were predicted PLP by EpiPred and 2 (p.Gln301Leu, p.Lys333Glu) were predicted BLB by EpiPred (Figure 1C and Supplemental Data Files 1 and 2). We used 4 readouts of variant effect that had been previously reported in models of *STXBP1* pathogenic missense variants. These included STXBP1 protein abundance (Figure 2A), solubility (Figure 2B), stability (Figure 2C), and the protein-protein interaction (PPI) between STXBP1 and STX1 (Figure 2D) (Supplemental Data Files 1 and 2) (10, 11).

Individually, these assays differentiated pathogenic from benign variants to some extent (Figure 2). In particular, the best discriminator was abundance; all of the PLP variants (*n* = 5) showed statistically significant reduced abundance, with a mean reduction to 14% (range 4%–42%) of WT levels, while only 5 of 10 BLB variants showed a significant change, with 4 variants reduced to approximately 71% (range 50%–73%) of WT, and one with 28% more protein than WT (Figure 2A). Similarly, protein stability was a good predictor of pathogenicity; all 5 PLP variants had statistically significant reduced stability (mean: 55%, range 38–64%), compared to only 3 of the BLB variants (*n* = 3/10) with a smaller reduction (mean 80%, range 75%–81%) (Figure 2C). There was a statistically significant correlation between stability and abundance (R = 0.73, *P* = 0.0005), as expected given the relatedness of these 2 assays. All but one of the PLP variants (p.Leu41Gln) were less soluble (mean 41%, range 32%–45%), compared with only one of the BLB variants (p.Val84Ile, 56% of WT).

The STX1:STXBP1 interactions were more complex. Two of the PLP variants (p.Arg292His and p.Arg406His) had statistically significantly reduced interactions (33% and 36% respectively); similarly, the predicted PLP variant p.Trp288Leu interacted with STX1 at only 6% of WT levels. However, one PLP variant (p.Ala101Pro) showed a greater than 2-fold increased interaction with STX1, as did 3 BLB variants, with a mean increase of 1.7-fold compared with WT (range 1.4–2.2). Overall, our VUS predictions by EpiPred were correct and the VUS that were predicted as either PLP or BLB clustered with known variants of each class.

In addition to these individual correlations, we considered the effects of the 4 readouts of variant effect in combination and generated a combined variant effect score using principal component analysis (Supplemental Table 4). The first principal component accounted for 56% of the total variance and effectively distinguished between a BLB and a PLP cluster of variants (Figure 3A). All assays contributed to this distinction, with stability and abundance among the largest contributors, and STX1 interaction at the least. Moreover, EpiPred had the strongest correlation with the combined variant effect score (0.72), compared with all other features, including global VEPs trained on ClinVar that were not used for EpiPred (Figure 3B, Supplemental Figures 6–8, and Supplemental Table 5).

Using this combined variant effect score, the p.Arg536His variant clustered with the benign variants. This BLB variant, present in gnomAD (*n* = 30), was predicted as pathogenic by EpiPred, PRESR, and most global VEPs, and had reduced abundance levels similar to PLP variants, with minimal effects on solubility and stability (Figure 2). However, p.Arg536His also demonstrated a greater than 2-fold increased interaction with STX1, which led to a combined variant effect score in the BLB range. This example highlights the importance of leveraging multiple assays as a readout of missense STXBP1 variant effect, as the detrimental impacts of a missense variant in one assay may potentially be ‘rescued’ by a different property of that variant in another.

### Evaluation of EpiPred in a holdout dataset demonstrates the ability to detect misclassified disease-relevant missense variants.

Leveraging the rapid pace of sequencing in population cohorts and individuals with epilepsy, we were able to establish a holdout set consisting of 43 unique PLP missense variants, primarily from a large *STXBP1* phenotyping study (30), as well as 78 unique missense BLB variants (Regeneron exome server and gnomAD v4) (Supplemental Table 6) (31). EpiPred, and indeed all VEPs investigated, exhibited reduced performance on the Holdout set relative to the truth set (Figure 4A, Supplemental Figures 9–13, and Supplemental Table 7). All top performers in the truth set, EpiPred (98%), Varity (97%), and ClinPred (96%), had reduced AUROCs in the holdout set: EpiPred (89%), Varity (88%), and ClinPred (80%) (Supplemental Table 7). To explore this reduced performance, we evaluated the accuracy of the model for PLP and BLB variants in EpiPred. In the BLB holdout set, EpiPred had a 92% (72/78) accuracy, with 3 variants predicted ambiguous and 3 predicted PLP, thus performing similarly to the truth set (96% accuracy). Moreover, ultra-rare variants present in GnomAD with an allele count less than 3 were also largely (85%) predicted to be benign (257/304), and only 10 variants (3%) predicted as PLP, with the remainder in the ambiguous range (Figure 4B). In contrast, In the PLP holdout set accuracy diminished from 96% accuracy in the truth set, to 67% (29/43) in the holdout set. This included 5 variants in the ambiguous range, and the remaining 9 PLP variants predicted more likely to be benign by EpiPred (Figure 4B). Thus, these findings revealed a potential misclassification of PLP variants in the cohort study used to derive the holdout set. The criteria for PLP designation were described in the publication as “a modified annotation pipeline and only included variants that were either pathogenic or likely pathogenic by ACMG criteria or de novo variants in the coding region of STXBP1”. Moreover, we found good agreement between EpiPred and ClinPred/Varity predictions, with both global VEPs predicting ‘PLP’ missense variants as benign (*n* = 8) or ‘ambiguous’ (*n* = 1) (Supplemental Figures 11 and 12, and Supplemental Table 8). We reevaluated the classification of these variants and determined that most would not meet the ACMG criteria for PLP classification; indeed 2 (p.Asn398Ser and p.Ser253Asn) are classified as VUS in ClinVar. More specifically, the inheritance of these variants was not reported, 3 were found in individuals without epilepsy or in atypical phenotypes (e.g., focal cortical dysplasia), and these amino acid locations (though different substitutions) were present in the general population (gnomAD). Finally, 2 variants, p.Asp207Gly and p.Asn398Lys, showed no change in any of the biochemical assays or STX1 interactions that were used to assess the core STXBP1 phenotype (Figure 4D and Supplemental Data Files 1 and 2).

We thus reclassified these 9 variants as VUS and reassessed EpiPred classification performance with 34 PLP (from *n* = 43 originally) and the same 78 BLB variants. We observed increased AUROC performance in both EpiPred (97.8%) and global VEPs, Varity (95%),and ClinPred (93%) (Figure 4C, Supplemental Figure 10, and Supplemental Table 9). The AUROC for all VEPs in this modified holdout set was also less variable, suggesting that reclassification led to more stable VEP predictions (Figure 4C). PRESR had reduced performance compared with EpiPred in the holdout set across all metrics (Figure 4, A and C, and Supplemental Figure 10). Both EpiPred, PRESR, and global VEPs (ClinPred/Varity) were still marginally outperformed by AlphaMissense (AUROC – 98.3%). This result suggests strong generalizability of AlphaMissense in the holdout set; however, EpiPred retained greater sensitivity, which is better aligned with the clinical utility goals of this VEP to identify individuals with pathogenic variants for clinical trial eligibility. In this context, sensitivity is particularly important because it reflects the ability to capture true positives rather than miss potentially eligible individuals. Collectively, while the holdout dataset had lower performance than the truth set, EpiPred generalized well and still outperformed global VEPs and PRESR. This analysis also highlights the power of EpiPred and global VEPs to accurately classify variants and identify false positives (and potentially misdiagnoses) in the literature.

### Gene-wide EpiPred simulation reveals specific regions susceptible to perturbation.

To facilitate dissemination of EpiPred to the scientific and epilepsy community, we generated EpiPred scores for all possible missense *STXBP1* variants (*n* = 3,939 total, *n* = 3,492 simulated, and *n* = 447 observed) resulting from a single nucleotide change (Supplemental Table 10, Figure 4B, and Figure 5A). The simulated variants exhibit a broad distribution of scores across the range, including 13% (466/3,492) predicted as PLP. This is expected, as a variant that has never been observed could indeed be pathogenic if it were to arise de novo in an individual with epilepsy. We also collated data from the literature that characterized missense variants (*n* = 26) using both cellular and animal model systems and generated an aggregate variant effect score for each variant (Supplemental Table 11) (10–15). This variant effect score, essentially a measure of how often a tested missense variant exhibited a phenotype, correlated reasonably well with EpiPred and PRESR, though EpiPred outperformed PRESR across metrics (Supplemental Figures 14–16, and Supplemental Table 12).

Using our comprehensive STXBP1 EpiPred simulation, we scored all VUS in the latest ClinVar dataset, which now includes an expanded set of 222 variants, up from 150 when we initiated this study (Figure 5B and Supplemental Tables 13 and 14). This includes 133 VUS from the original dataset, as well as 32 simulation or ‘new’ variants, which had comparable median EpiPred-P^PLP^ scores, 0.25 for new VUS, and 0.44 for previously identified VUS. There were, however, more VUS that were predicted to be pathogenic in the previously identified VUS (31/133–23%) as compared to the newly identified VUS (3/32–9%), though this difference was not statistically significant (Fisher’s Exact Test, Odds Ratio: 0.34, *P*-value of 0.092). There were also multiple VUS that were previously classified as BLB due to presence in gnomAD more than thrice, this group had a lower median EpiPred-P^PLP^ score (0.09). In addition, only 1 variant in this BLB group is predicted to be pathogenic (1/48–2%), which is far less than all VUS (40/222–18%) (Fisher’s Exact Test, Odds Ratio: 0.097, *P*-value of 0.0032). Finally, there were also 9 variants that were previously classified as PLP and have subsequently been reclassified as VUS. This includes 4 variants that were classified as benign by EpiPred, 2 variants that we reclassified from the holdout set (p.Asn398Ser, p.Ser253Ans) and 2 variants from the truth set p.Lys7Glu (EpiPred-P^PLP^ score 0.53) and p.Ser80Pro (EpiPred-P^PLP^ score 0.43) (Supplemental Figure 5 and Supplemental Table 10).

Overall, this reclassification analysis further demonstrates the utility of EpiPred to reclassify STXBP1 missense variants. To facilitate this, we developed a webapp, EpiPred (https://carvill-lab.shinyapps.io/epipred-shiny/) a Shiny-based user interface for clinicians, families and researchers to access our data and use the model to aid interpretation of STXBP1 missense VUS. We envision a workflow whereby EpiPred can assist in the reclassification of variants from clinical genetic testing, prioritizing high yield variants for experimental characterization in the instance of predicted pathogenicity, and revisiting the ACMG classification criteria in the instance of predicted benignity (Figure 5D).

To visualize whether predicted pathogenic/benign variants cluster in specific regions of STXBP1, we aggregated EpiPred scores at each variant position and projected these onto the STXBP1 3D structure (Figure 5C). Rather than clustering within specific domains, which STXBP1 lacks, we found that known and predicted PLP variants tend to localize within the central core of the protein. This localization may underlie the reduced abundance and stability observed in STXBP1 proteins with pathogenic missense variants; highlighting a subset of variants and patients that could benefit from protein-stabilizing–based therapies such as PBA.

## Discussion

Herein, we present EpiPred, a publicly accessible gene-specific classifier for pathogenicity prediction in the gene, *STXBP1*. EpiPred outperformed or matched leading global VEPs, and the STXBP1-specific, PRESR, in most clinically relevant metrics and, importantly, provided gene-calibrated sensitivity and interpretability aligned with VUS triage and trial-enrichment goals. EpiPred predictions were validated through 4 established variant effect assays that comprise a core *STXBP1* missense variant phenotype. This phenotype consists of reduced STXBP1 abundance, solubility, stability, and generally less interaction with syntaxin-1, though some PLP variants showed enhanced binding. While there was a strong correlation between EpiPred and both our and published experimental data, there was a small subset of conflicting variants where EpiPred (and other VEP) predictions did not match the known class. These variants represent a unique opportunity to understand variant effect and the complex interaction of protein features, but also the ability to identify misclassified variants. We show that roughly a quarter of VUS could be pathogenic and develop an EpiPred Shiny app to facilitate the accessibility of variant interpretation and utility in clinical care. Beyond precision diagnostics, our computational and variant effect data consistently highlight reduced STXBP1 abundance as a key driver of pathogenicity. This insight paves the way for targeted precision therapies addressing protein abundance and instability, including promising approaches such as PBA-based treatments (28).

EpiPred outperforms all other global VEPs and PRESR, across multiple metrics, with an AUROC of 98%, as well as specificity, sensitivity and accuracy of 96% in the truth set, with a 60:40 split of training and test datasets. These metrics were reduced in a modified holdout dataset, which excluded 9 VUS (see below), to an AUROC of 97.8%, specificity of 83%, sensitivity of 100%, and accuracy of 88%. Even in the holdout dataset, EpiPred outperformed all global VEPs and PRESR, with only AlphaMissense (AUROC 98.3%) slightly outperforming EpiPred in the holdout set; by comparison, the AUROC of AlphaMissense in the truth set was 94%. Thus, overall, EpiPred outperformed all other global VEPs, despite being trained on a substantially smaller dataset. This is comparable to the handful of other gene-specific ensemble VEPs, particularly those for *BRCA1*/2, which demonstrated a 96% sensitivity and 98% sensitivity (25). Collectively, we demonstrate the utility of gene-specific VEPs for clinically relevant genes, and our goal in EpiMVP is to extend this approach to other non-ion channel–related genes, prioritizing those more commonly implicated in epilepsy: *SYNGAP1*, *SLC6A1*, and *PCDH19*. These results are likely to extend beyond the epilepsies, to all clinically relevant genes, as has also been the case for *BRCA1/2*.

While EpiPred demonstrates strong predictive performance, it remains essential to experimentally characterize variants and uncover how distinct features of STXBP1 missense variants interact and contribute to disease mechanisms. Experimental measurements also provide evidence to correctly classify any false positive or negative variants which are likely present in EpiPred and other VEPs. Here, we also propose a framework by which EpiPred could be deployed for reclassification of STXBP1 missense variants including prioritizing variants for experimental validation. Specifically, VUS predicted to be pathogenic by EpiPred, as well as known pathogenic variants predicted to be benign, warrant closer examination.

For instance, one of the most intriguing variants in this study was p.Arg536His, classified as BLB, and present in 30 individuals in gnomAD, primarily in individuals of non-Finnish European ancestry (*n* = 27). However, EpiPred and, indeed, all other global VEPs and PRESR, predict p.Arg536His as likely pathogenic (EpiPred-P^PLP^ score = 0.96). In our heterologous system, this variant exhibited reduced abundance and solubility compared with WT, in keeping with the majority of the pathogenic variants. However, protein stability was unchanged, while the variant displayed a 2-fold increased binding of STX1. Thus, one could speculate that increased STX1 binding might ‘rescue’ the reduced abundance of the mutant protein. In keeping with this logic, the combined variant effect score suggested benignity, as the variant clustered with other BLB variants. This observation highlights the strength of evaluating multiple biochemical consequences of a missense variant and provides motivation for the combined variant effect score. Importantly, this finding also highlights key residues for the development of targeted therapeutics in the form of allosteric regulators.

Another key example is p.Ala101Pro, a PLP variant that also had an approximately 2-fold increase in STX1 interaction, but greatly reduced abundance, stability, and solubility, which is presumably insufficient to offset the increased STX1 interaction. In this instance, both EpiPred and the combined variant effect score correctly predict this variant as pathogenic. We also speculate that a number of conflicting variants may display similar complex interactions between the SNARE protein binding partners as well as the biophysical and biochemical properties of the mutant protein. Overall, these 2 examples, as well as the other 15 discordant variants, should be prioritized for experimental examination, both within our system and in more pathologically relevant models, such as iPSC-derived neurons and animals. For instance, in our EpiMVP companion paper we modeled a truncation (p.Gly544Val-fs*2) and a missense p.Tyr75Cys (EpiPred-P^PLP^ score = 0.98) variant derived from individuals with DEE in human iPSC-derived neural precursors and glutamatergic neurons. Similar to our overexpression data, we demonstrate decreased STXBP1 and STX1 protein levels, as well as decreased solubility. Glutamatergic neurons from these patients also exhibited reduced synaptogenesis, increased neurodegeneration, and decreased neuronal activity and network synchrony. Collectively, our studies implicate the core mutant STXBP1 protein phenotype as an integral molecular component of disease pathophysiology. A systematic analysis of conflicting variants offers a valuable opportunity to deepen our understanding of variant effect and the intricate interplay of features underlying the core STXBP1 missense variant phenotype.

Our experimental and computational analyses reveal that, rather than clustering in specific domains, which STXBP1 does not have, PLP variants tend to occur in the middle core of the protein. Further, RSA, a measure of how buried an amino acid residue is within a protein, was one of the primary drivers of EpiPred, suggesting the core of STXBP1 is more likely to harbor disease-related variants. This aligns with global protein predictions, where pathogenic variants generally tend to be more buried within a protein than polymorphisms that tend to be exposed (32). RSA is also a proxy for stability of a protein, which was one of the strongest experimental discriminators of pathogenic and benign variants, again highlighting the strength of combining biochemical measurements and in silico predictions.

There are now multiple studies, including our companion EpiMVP paper, which show that PBA can rescue phenotypes associated with STXBP1 loss in yeast, *C*. *elegans*, mouse neurons (15), and iNeurons. These studies are timely, as PBA is currently in clinical trials for *STXBP1*-related disorder and *SLC6A1*-related NDD (28). In our companion EpiMVP paper, we show that 2 patients in this PBA trial, with a missense variant, p.Arg292Pro (EpiPred-P^PLP^ score = 0.98), and a truncating variant (p.Ser311PhefsX3) had reduced seizure frequencies after treatment (personal communication). Collectively, our data suggest that individuals with missense variants that are confirmed or predicted to be unstable might benefit from PBA, or similar, therapies. In fact, stability of mutant proteins was one of the biggest drivers of pathogenicity of EpiPred. In the future, high-throughput methods such as VAMP-seq, which uses GFP-tagged proteins and massively parallel sequencing to measure steady-state protein abundance, could be used to create a comprehensive map of STXBP1 missense variant abundance (33). This approach was used successfully to evaluate the entire coding sequence of *PTEN* (34), *CYP2C9* (35), *Parkin* (36), and other clinically relevant genes, revealing key domains prone to instability or targeted by proteostasis.

Further, for variants that are classified in ClinVar as pathogenic but predicted by EpiPred to be benign, there is a strong case that these variants may indeed be benign or neutral. We propose a framework for deployment of EpiPred after initial classification by clinical genetic testing. For those variants that EpiPred predicts as benign, experimental characterization is necessary, including multiple ‘PLP’ variants in the holdout dataset that are more likely benign. The vast majority of pathogenic variants in the holdout dataset were from a large cohort study (*n* = 534) examining the clinical features of STXBP1-related NDD (37). We reclassified 9 of these variants as VUS, using ACMG criteria, and, indeed, 2 (p.Asn398Ser and p.Ser253Asn) are classified as VUS in ClinVar. These decisions were based on the following evidence: good agreement between EpiPred and other global VEPs that these variants are more likely benign, the inheritance of these variants was not reported, and these amino acid locations (though different substitutions) were present in the general population (gnomAD).

Variant effect assays for 2 of these variants (p.Asp207Gly and p.Asn398Lys) demonstrated no change in STXBP1 abundance, stability, solubility, or interaction with STX1, in keeping with WT STXBP1. Thus, collectively, it is likely that some of these VUS, except for p.Asp207Gly as discussed below, are misdiagnosed, and further characterization in our iNeuron system is warranted. In addition, 3 of these variants were identified in individuals without epilepsy, or in atypical phenotypes (e.g., focal cortical dysplasia); we speculate that these variants may indeed be pathogenic, just not for epilepsy, and that EpiPred, having been trained solely on epilepsy-related variants, may misclassify these variants. Again, further characterization, using multiplexed assays of variant effect (MAVEs) and iNeurons, is needed to support this hypothesis. Moreover, these insights will be advanced as additional genotype-phenotype correlations and as more individuals with rare *STXBP1* variants are identified in individuals with neurological conditions without epilepsy.

Despite the high predictive power of EpiPred, it still has limitations. For instance, in the holdout dataset, the variant p.Asp207Gly was predicted as benign by all global VEPs, PRESR and EpiPred (EpiPred-P^PLP^ score = 0.13) and had the same biochemical properties and syntaxin interaction as WT STXBP1 in variant effect studies. However, iPSCs derived from the patient carrying this variant revealed reduced mRNA and protein levels of STXBP1, possibly due to the generation of a cryptic splice site (12). Unfortunately, this aberrant splicing was not explored in the iPSC model, and an additional missense variant (p.Arg235Gln) showed reduced mRNA levels, suggesting that reduced mRNA is a more general feature of pathogenic missense variants that warrants further study.

However, this p.Asp207Gly variant does highlight an important limitation of our study: that missense variants that disrupt splicing via alteration of an exonic splice enhancer cannot be detected in EpiPred or our heterologous overexpression-based system. The most popular splice-site prediction tool, SpliceAI, did not predict p.Asp207Gly to disrupt splicing; however, the misclassified PLP variant, p.Glu487Asp with a benign EpiPred-P^PLP^ score (0.48) is predicted to disrupt an existing donor site and create a new one approximately 100 bp upstream. Thus, tools such as SpliceAI (38) and other predictors focused on motifs within exons, should be incorporated into EpiPred and other gene-specific predictors in the future.

To facilitate the utility of, and access to, EpiPred, we built a user-friendly Shiny-based web app that allows users to input their *STXBP1* missense variant of interest and obtain the EpiPred-P^PLP^ score. Furthermore, the user receives information to aid in interpretation, including an explainer of variant classes and what the prediction means, as well as where the variant lies in the 3D structure. Our intention is that the ‘Check my Variant’ tab be utilized by clinicians, genetic counselors, parents, and caregivers, to add interpretation of an STXBP1 missense variant, though all results should be considered in combination with other clinical and genetic data, preferably with a genetic counselor and the clinical care team. We also make the same information available in the ‘For Researchers’ tab, where EpiPred predictions for every possible missense variant can be compared to global VEP predictions, correlations between VEPs can be visualized, and other features including gnomAD allele counts and amino acid positions are delineated.

As part of the design of the EpiPred Shiny app, we generated every possible missense variant that could result from a single nucleotide change (*n* = 3,492 missense variants). Of these, 13% (*n* = 466) are predicted to be pathogenic by EpiPred. Moreover, of the 151 VUS in our original ClinVar dataset, 23% (*n* = 35) have scores in the pathogenic range, thus suggesting that many existing and putative VUS may be related to the disease phenotype. While global VEPs and variant effect data are routinely used as ‘supporting’ and ‘supporting to moderate’ evidence in the ACMG framework, our study suggests that integrating EpiPred could have high clinical utility.

In the current study, we propose a model whereby EpiPred is deployed after initial variant classification leveraging the webapp and community engagement. Future systematic incorporation of EpiPred for reclassification will most readily be achieved through the Epilepsy Gene Variant Curation Groups within the ClinGen initiative. Within this framework, it will be important to not only flag VUS that may be pathogenic to facilitate a genetic diagnosis but also examine those VUS that are currently misclassified as pathogenic. These insights are also integral given the emerging precision therapy opportunities for individuals with STXBP1-related NDD, including the clinical trial for PBA. Identification of patients with a precise genetic diagnosis is crucial for enrollment in a precision therapy trial. Finally, both our computational and variant effect analyses demonstrate abundance and stability as key features associated with *STXBP1* pathogenic missense variants. Coupled with VAMP-seq in the future, this feature will allow us to a-priori define those missense variants that perturb abundance, and, thus, the individuals that may best benefit from PBA or more targeted small molecule chaperones. Finally, although this study focuses on STXBP1, the EpiPred framework is broadly relevant to rare disease genomics. STXBP1 serves as a clinically meaningful proof-of-principle for gene-tailored variant effect prediction, but the same strategy could be adapted to other disease-related genes with sufficient curated variant data. Such extensions could help prioritize missense VUS for functional validation, support variant reclassification, and improve identification of individuals who may benefit from genotype-informed clinical trials across a wider range of Mendelian and neurodevelopmental disorders.

## Methods

### Sex as a biological variable

Sex was not a variable in this study, *STXBP1* pathogenic variants are identified in males and females equally, with the same clinical presentation.

### Supervised learning to develop EpiPred-STXBP1

To develop our training set, missense variants were obtained from ClinVar, gnomAD v3 (variants with an allele count > 3). After removing duplicates, our dataset contained 80 benign or likely benign (BLB) variants (gnomAD and ClinVar) and 96 pathogenic or likely pathogenic (PLP) variants (ClinVar) defined as per ACMG criteria. This training dataset is referred to as the Truth Set (TS) (Supplemental Table 1). We further identified 150 VUS, listed as either VUS or conflicting in ClinVar (Supplemental Table 2). Each variant was annotated with 42 features including amino acid properties, conservation, stability predictions, solvent accessibility predictions and other VEPs, none of which were trained on ClinVar data (*n* = 42; listed in Supplemental Table 3). Empty values were replaced with the median value for that feature. Initially, multiple models including multiple linear regression, decision tree, and random forest were explored. We identified random forest as a model which could accurately perform the classification task, in agreement with previous gene-specific VEPs (8). A random forest model was developed using the Python scikit-learn package and hyperparameters were tuned using five-fold cross validation. EpiPred-*STXBP1* was trained using 160 trees with maximum depth 5 on a random 60% subset of the TS and tested on the remaining 40%. Leave-one-out cross validation (LOOCV) was used to gauge model classification on unseen data. The negative mean absolute error from LOOCV was 0.155. Performance of EpiPred-*STXBP1* and competing in silico pathogenicity predictors were investigated using the R pROC package.

### Validation of EpiPred-STXBP1 on a holdout dataset

Missense variants were curated from gnomAD v4 (new variants not present in v3), the Regeneron million exome server, and the manuscript from Xian et al. (30) For BLB variants, we required an allele count of > 2 in either gnomAD v4 or the Regeneron exome server. Variants that overlapped with the TS were removed. The final Holdout Set (HS) contained 121 unique missense variants, including 78 BLB and 43 PLP (Supplemental Table 8).

### Gaussian mixture modeling

The mclust R package was used to develop a univariate, unequal variance model based on the EpiPred scores for TS and HS variants (39). We found that the upper 95% mean confidence interval for BLB variants was 0.625179 while the lower 95% mean confidence interval was 0.8711709. Based on this modeling, we considered variants with EpiPred scores in the interval between these 2 values to be ambiguous.

### Global and local model explanations

To investigate global and local model decisions, we utilized a tree explainer within the Python SHapley Additive exPlanations (SHAP) package (29). At the global level we used these interpretations to determine what features drove EpiPred generally across the STXBP1 missense variant landscape. We leveraged local explanations for each variant, particularly those with conflicting EpiPred predictions to determine whether certain features drove these misclassifications.

### Characterization of STXBP1 missense variant effect

A subset of variants (*n* = 20) was selected for assessment of variant effect; experimenters were blinded to the classification (PLP/VUS/BLB) of each variant. At least 3 independent replicates were tested for each variant. Moreover, all quantification was performed by 2 independent experimenters blinded to a variant identity. *t*-tests were performed for all pairwise comparisons of each variant to WT; significance threshold was 0.05.

The plasmid (pPB-CAG-EGFP Addgene 40973), with the EGFP coding region replaced with either WT *STXBP1* or one of the missense variants, was transformed into DH5α Competent Cells (18265017, Thermo Fisher Scientific). Three bacterial clones were picked and inoculated into LB broth with 100μg/ml carbenicillin. The bacteria were cultured for 16–18 hours. The plasmid DNA was prepared with GenCatch Plasmid DNA Mini-Prep Kit (2160-250, Epoch Life Science, Inc).

#### STXBP1 abundance assay.

HEK293T cells (ATCC, CRL-3216) were maintained with Corning DMEM [+] 4.5 g/L glucose, L-glutamine, sodium pyruvate (45000-304, VWR), supplemented with 10% Fetal Bovine Serum, value (A5256701, Thermo Fisher Scientific) and 100 U/ml Penicillin-Streptomycin (10,000 U/mL) (15140122, Thermo Fisher Scientific). The day before transfection, 0.5 × 10^6^ cells/well were plated into 6-well plates. The cells were transfected with 1 μg of the same batch-prepared plasmid with PolyJet In Vitro DNA Transfection Reagent (SL100688, SignaGen Laboratories). With 48-hour post-transfection, cells were washed with cold 1XPBS and lysed using RIPA buffer containing protease inhibitors. The protein concentration was determined with Pierce BCA Protein Assay Kits (23227, Thermo Fisher Scientific). Equal amounts of protein were separated by SDS-PAGE and transferred to polyvinylidene difluoride (PVDF) membranes (1620177, Bio-Rad Laboratories, Inc.). After blocking, blots were probed with Munc18-1 (D406V) Rabbit mAb #13414 (1:1000, Cell Signaling Technology). Blots were stripped and reprobed for Alpha Tubulin with Alpha Tubulin Monoclonal antibody (1:2000, 66031-1-Ig, Proteintech Group).

#### STXBP1 solubility assay.

HEK293T cells in 60mm Petri dishes were transfected with different amounts of plasmid DNA to ensure a similar protein abundance, according to the plasmid DNA expression potency. With 48 hours post-transfection, the cells were washed with PBS + 1mM MgCl2. After that, 1XPBS+0.1% Triton X 100+ protease inhibitors+ Pierce Universal Nuclease for Cell Lysis (1:1000, 88700, Thermo Scientific) was added to the dishes. The cells were collected into 1.6ml Eppendorf tubes with a cell scraper. The tubes were placed on an agitation shaker and shaken gently in the cold room for 1 hour. The lysates were pelleted at 4°C, 20,000 × *g* for 20 minutes. The supernatants (containing the soluble STXBP1 protein) were transferred into new Eppendorf tubes and placed in ice. The pellets were resuspended with 1% SDS in PBS. The tubes were placed on a shaker at 30°C at 300 rpm, for 20 minutes, and then centrifuged at 10,000 × *g* for 20 minutes at 23°C. The pellets were removed. The supernatants stood at room temperature during the protein concentration measurement with a BCA kit. The protein concentration was normalized to 1–2mg/ml with PBS with 0.1% Triton X 100 or 1% SDS in PBS. 15 μl of the lysates were loaded into each lane after mixed with 4xsample buffer+DTT and warmed at 42°C for 10 minutes. Soluble and insoluble fractions were collected. Equal amounts of each fraction were separated by SDS-PAGE and transferred to PVDF membranes. After being blocked, blots were probed with Munc18-1 (D406V) Rabbit mAb #13414 (1:1000, Cell Signaling Technology) or Monoclonal ANTI-FLAG M2 antibody produced in mouse (1:1000, F1804, MilliporeSigma). Blots were stained with Revert 700 Total Protein Stain for Western Blot Normalization (926-11011, LI-COR Technology) to confirm equal loading.

#### STXBP1 stability assay.

HEK293T cells in 60 mm Petri dishes were transfected with different amounts of plasmid DNA to ensure a similar protein abundance, according to the plasmid DNA expression potency. 40 hours post-transfection, cells were then treated with 10 μg/ml cycloheximide or vehicle control for 8 hours. Cells were lysed using RIPA buffer containing protease inhibitors. Equal amounts of each fraction were separated by SDS-PAGE and transferred to PVDF membranes. After being blocked, blots were probed with Munc18-1 (D406V) Rabbit mAb #13414 (1:1000, Cell Signaling Technology) or Monoclonal ANTI-FLAG M2 antibody produced in mouse (1:1000, F1804, MilliporeSigma). Blots were stained with Revert 700 Total Protein Stain for Western Blot Normalization (926-11011, LI-COR technology) to confirm equal loading.

#### Co-immunoprecipitation of STXBP1 and STX1.

TRE-T-SNARE stable cells were generated by transfection of HEK293T cells with 1 μg of pPB-TRE-T-SNARE (pPB-TRE-STX1A-P2A-SNAP25-IRES-VAMP1) plasmid DNA + 1 μg of pPB-CAG TET-ON-mCherry plasmid DNA + 1 μg of pPB-CAG- PuroR plasmid DNA, 3 μg of pPB-CAG- piggyBac transposase plasmid DNA with DNA PolyJet In Vitro DNA Transfection Reagent (SL100688, SignaGen Laboratories). The transfected cells were selected with 1 μg/ml puromycin (ant-pr-1, InvivoGen) for 3 days. The selected cells were growing to form clones on the plates. The clones were picked and propagated in 24-well plates. The T-SNARE (STX1A, SNAP2, and VAMP1) protein abundance was verified with Western blotting.

TRE-T-SNARE cells in 6-well plates were transfected with different amounts of plasmid DNA to ensure a similar protein abundance, according to the plasmid DNA expression potency. 8 hours post-transfection, The T-SNARRE protein expression was induced with 1 μg/ml Doxycycline (A4052, APExBIO Technology LLC) 40 hours later, the cells were lysed with IGEPAL CA-630 lysis buffer (50 mM Tris-Cl (pH7.5), 10% Glycerol, 150 mM NaCl, 0.5% IGEPAL CA-630) containing Protease Inhibitor Cocktail (EDTA-Free, 100X in DMSO) (B14002, Selleck Chemicals LLC), Phosphatase Inhibitor Cocktail (2 Tubes, 100X) (B15002, Selleck Chemicals LLC), and 10 mM 2-mercaptoethanol, followed by immunoprecipitation using anti-FLAG (B23102; Selleck Chemicals LLC). Following SDS-PAGE and transfer to PVDF membrane, samples were probed with anti-STX1 antibody (1:1000, 18572, Cell Signaling Technology).

### Principal component analysis and k-means clustering of variant effect data

Principal components were calculated from variant effect data on all variants. PC1 and PC2 accounted for most of the variation in the dataset (60.9% and 21.2%, respectively). PC1 is correlated with all 4 individual variant effect measurements; we use PC1 as a metric representing a combined variant effect score. K-means clustering was performed on principal components with 2 clusters specified (K = 2).

### Statistics

All statistical models and tests used in this study are described in the methods section for each component. For 2-tailed Student’s *t* tests of immunoblot quantification data, STXBP1 variants were tested against STXBP1 WT. *P* values of less than 0.05 were considered significant.

### Study approval

No human or animal studies were conducted in this manuscript.

### Data availability

All code is available on the Carvill Lab Github. The most recent version is available (https://github com/carvill-lab/STXBP1-epiPred) and the version of record for this publication is archived in Zenodo (https://doi.org/10.5281/zenodo.23171423

## Manuscript information

### Author contributions.

Conceptualization: GLC, MER, VAP, LLI, YW, and JP. Methodology: GLC, MER, VAR, LLI, YW, JMP, JDC, CW, and HCM. Investigation: JDC, CW, CB, JRG, JSL, AMG, JHH, SS, LTD, LLI, MDU, GLC, YW, JMP, HCM, MER, and VAP. Funding acquisition: GLC, MER, VAP, LLI, YW, and JP. Supervision: GLC, MER, and VAP. Writing – original draft: GLC and JDC. Writing – review & editing: all.

### AI disclosure.

No AI was used in the preparation of this manuscript.

### Conflict of interest.

The authors have declared that no conflict of interest exists.

### Funding.

This study was supported by the following:

- NIH EpiMVP CWOW NINDS U54NS117170 (to LLI, JP, MER, GLC, YW, MDU).

This work is the result of NIH funding, in whole or in part, and is subject to the NIH Public Access Policy. Through acceptance of this federal funding, the NIH has been given a right to make the work publicly available in PubMed Central.

## Supplementary Material

Supplemental data

Unedited blot and gel images

Supplemental tables 1-14

Supporting data values

## Figures and Tables

**Figure 1:**
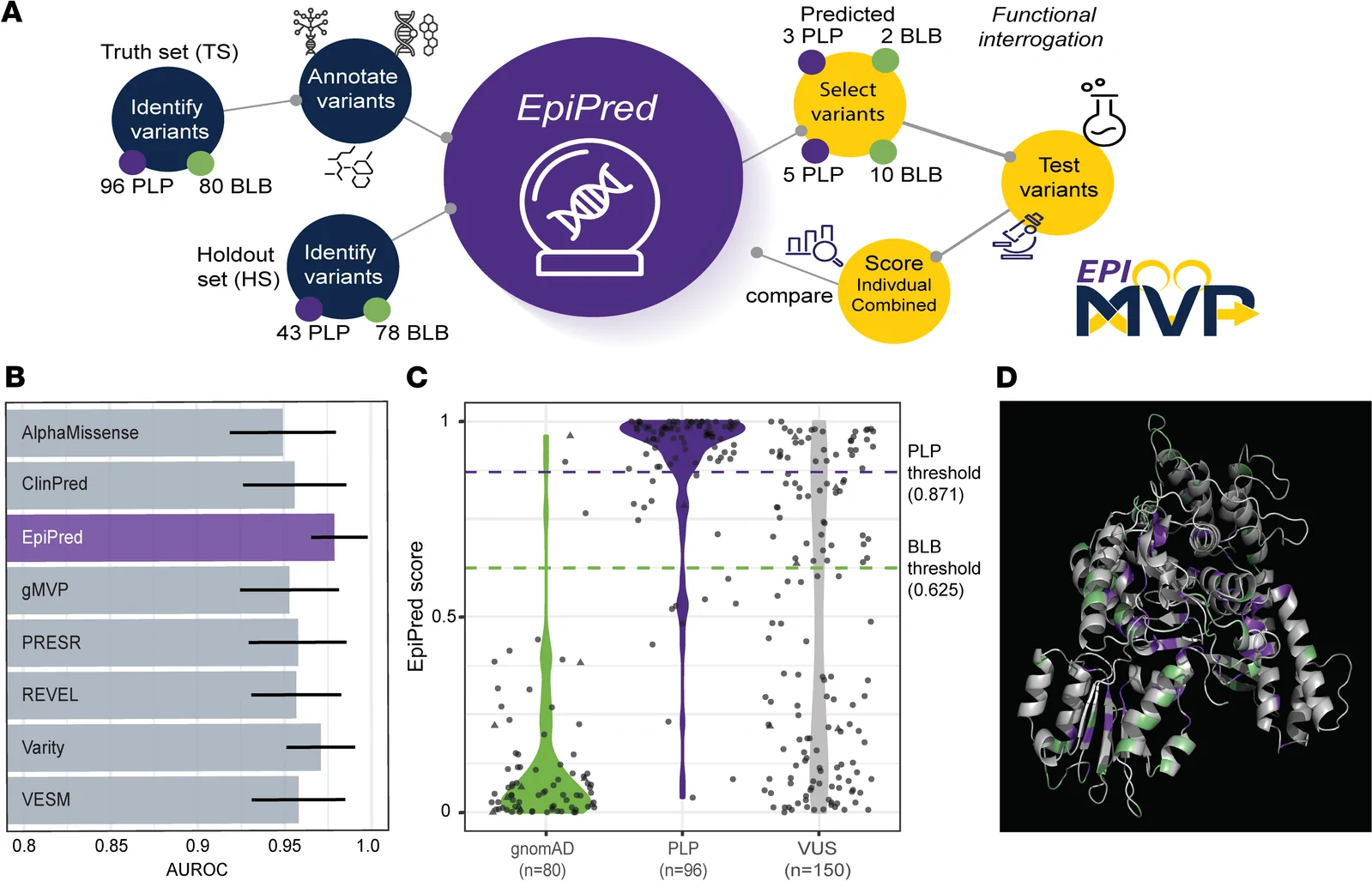
Supervised learning facilitates the generation of an *STXBP1*-specific variant effect predictor, EpiPred. (**A**) Overview of the approach adopted for the generation of the EpiPred model, including the input datasets (blue) and the variant effect testing of the EpiPred predictions using 3 biochemical assays (stability, abundance, solubility) and STX1 interaction in an overexpression model system (yellow). (**B**) Comparison of area under the receiver operator curve (AUROC) for EpiPred and a subset of high-performing globally trained VEPs, and the STXBP1-specific tool, PRESR, across the full truth set. Error bars give the 95% confidence interval. (**C**) EpiPred Probability of PLP (EpiPred-P^PLP^) score distributions in the full truth set separated by class as well as the full set of VUS. EpiPred-P^PLP^ scores of 1 suggest pathogenicity, while scores of 0 suggest benignity (**D**) EpiPred-P^PLP^ output for known PLP and BLB variants used in the truth set mapped onto hSTXBP1 predicted structure (AlphaFold structure: AF-P61764-F1-model_v4), EpiPred-P^PLP^ scores close to 1 are shown in purple, while scores close to 0 appear in green.

**Figure 2:**
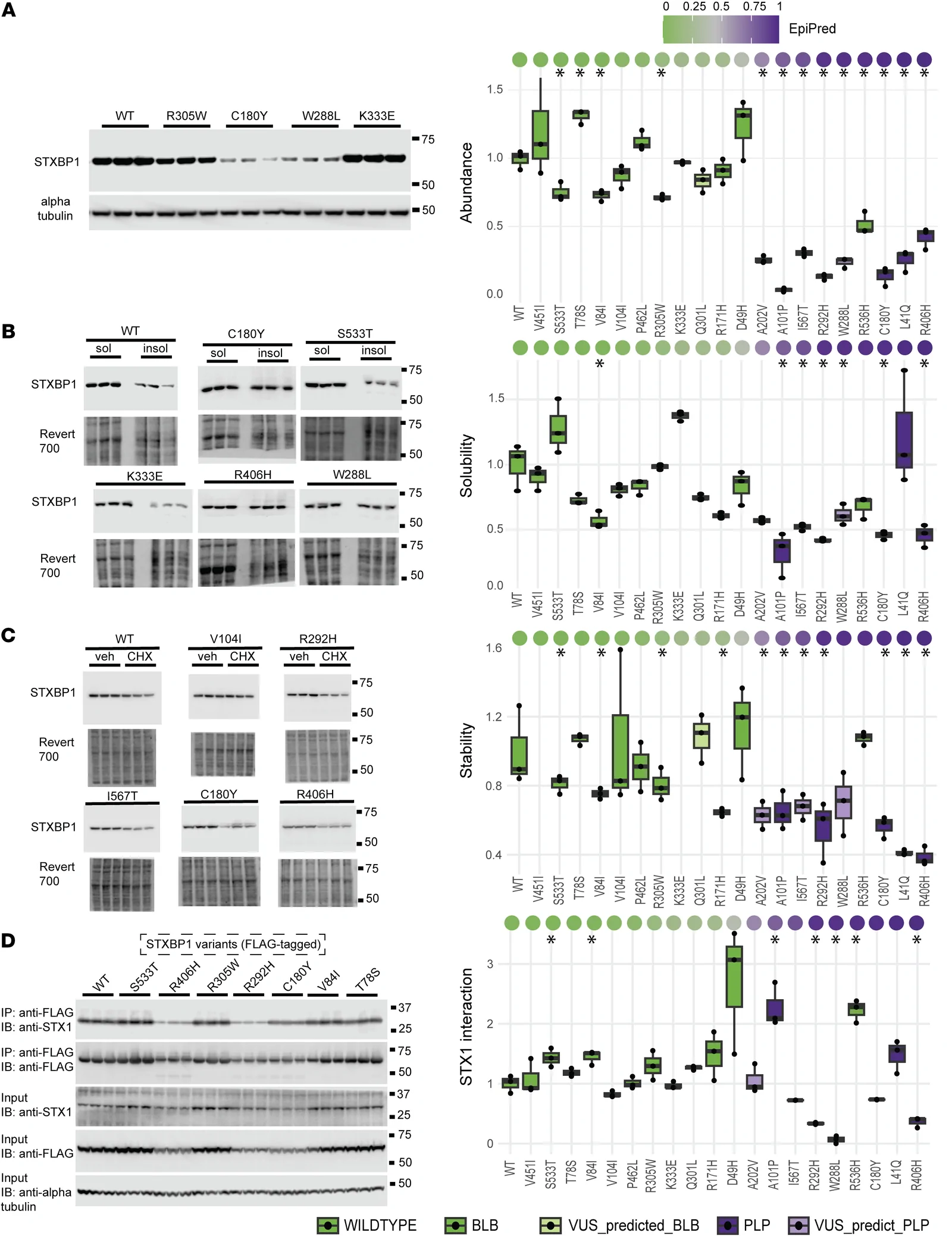
Biochemical assays and syntaxin 1 interaction distinguish pathogenic from benign *STXBP1* missense variants. Data from representative Western blots (left) and quantification (right) are plotted for the WT STXBP1 and 20 missense variants. Each assay is plotted separately: (**A**) Abundance, (**B**) Stability, (**C**) Solubility, and (**D**) STX1 PPI. Scores are normalized to the WT reference allele score. The quantified data is sorted, left to right, by EpiPred-P^PLP^ score from lowest (likely benign) to highest (likely pathogenic) and colored by class (WT, PLP, BLB, VUS predicted PLP/BLB). *n* = 3, *P < 0.05 (2-tailed Student’s *t* test); all raw data and *P* values in Supplemental Data Files 1–2.

**Figure 3:**
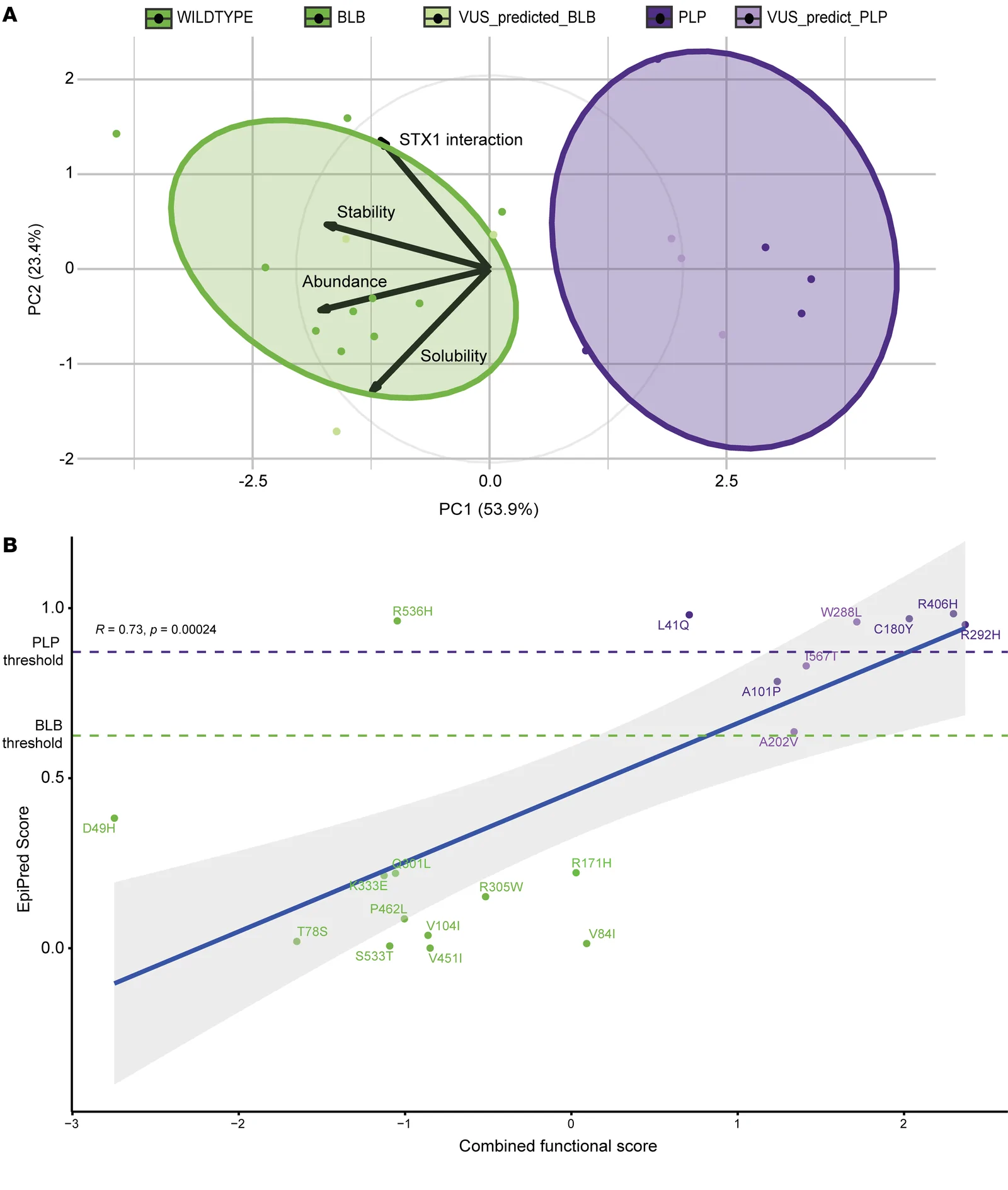
Integrating 4 variant effect assays with principal component analysis. (**A**) Biplot showing distinct BLB and PLP clusters within the dataset across 2 principal components, with PC1 explaining most of the variance in the data. (**B**) Scatterplot of EpiPred-P^PLP^ scores and combined variant effect score (PC1). EpiPred-P^PLP^ scores are highly correlated with the combined variant effect score. Variant and colored by class: PLP, dark purple; BLB, dark green; VUS predicted PLP, light purple; VUS predicted BLB, light green. (*r* = 0.73; *P* = 0.00024 Pearson).

**Figure 4:**
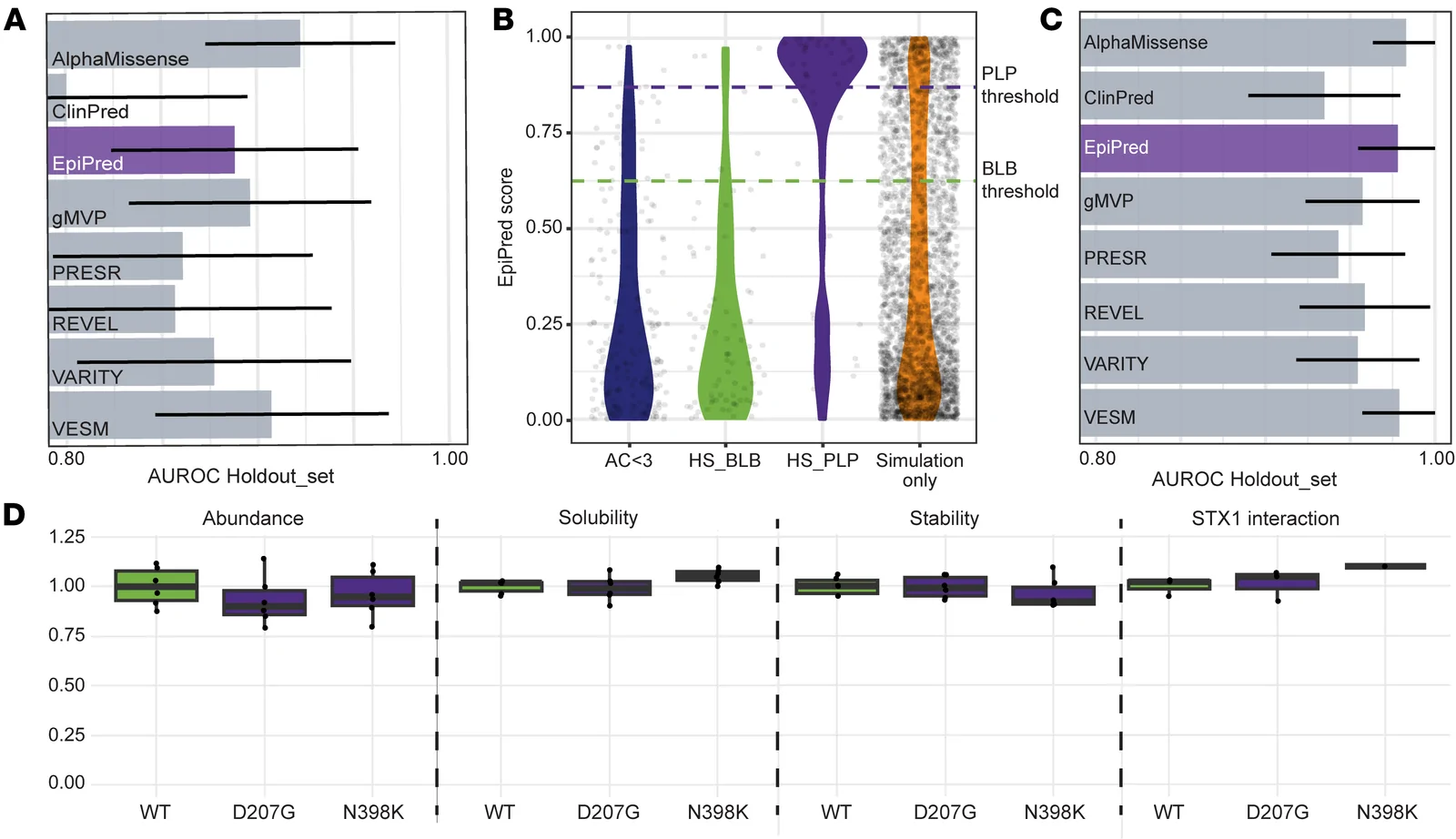
EpiPred classification of a holdout dataset. (**A**) Comparison of area under the ROC curve (AUROC) for EpiPred and a subset of high-performing globally trained VEPs and PRESR across the holdout dataset demonstrates reduced performance of EpiPred, error bars show the 95% CI. (**B**) EpiPred-P^PLP^ score distributions in the full holdout data set show the conserved performance for BLB variants, but a larger percentage of PLP variants with EpiPred-P^PLP^ scores in the benign/neutral range. Ultra-rare variants in GnomAD (allele count < 3) have scores more similar to BLB variants, while simulated variants occur across the range. EpiPred-P^PLP^ scores of 1 suggest pathogenicity, while scores of 0 suggest benignity. (**C**) Comparison of area under the ROC curve (AUROC) for EpiPred, PRESR, and a subset of high-performing globally trained VEPs across the holdout dataset with reclassified variants, including the exclusion of 9 VUS from the PLP set, demonstrates improved EpiPred performance, on par with AlphaMissense, error bars show the 95% CI. (**D**) Modeling of 2 misclassified variants, reclassified to VUS, which demonstrate properties similar to WT for abundance, stability, solubility and syntaxin interaction. *n* = 6, *P < 0.05 (2-tailed Student’s *t* test); all raw data and *P* values in Supplemental Data Files 1–2.

**Figure 5:**
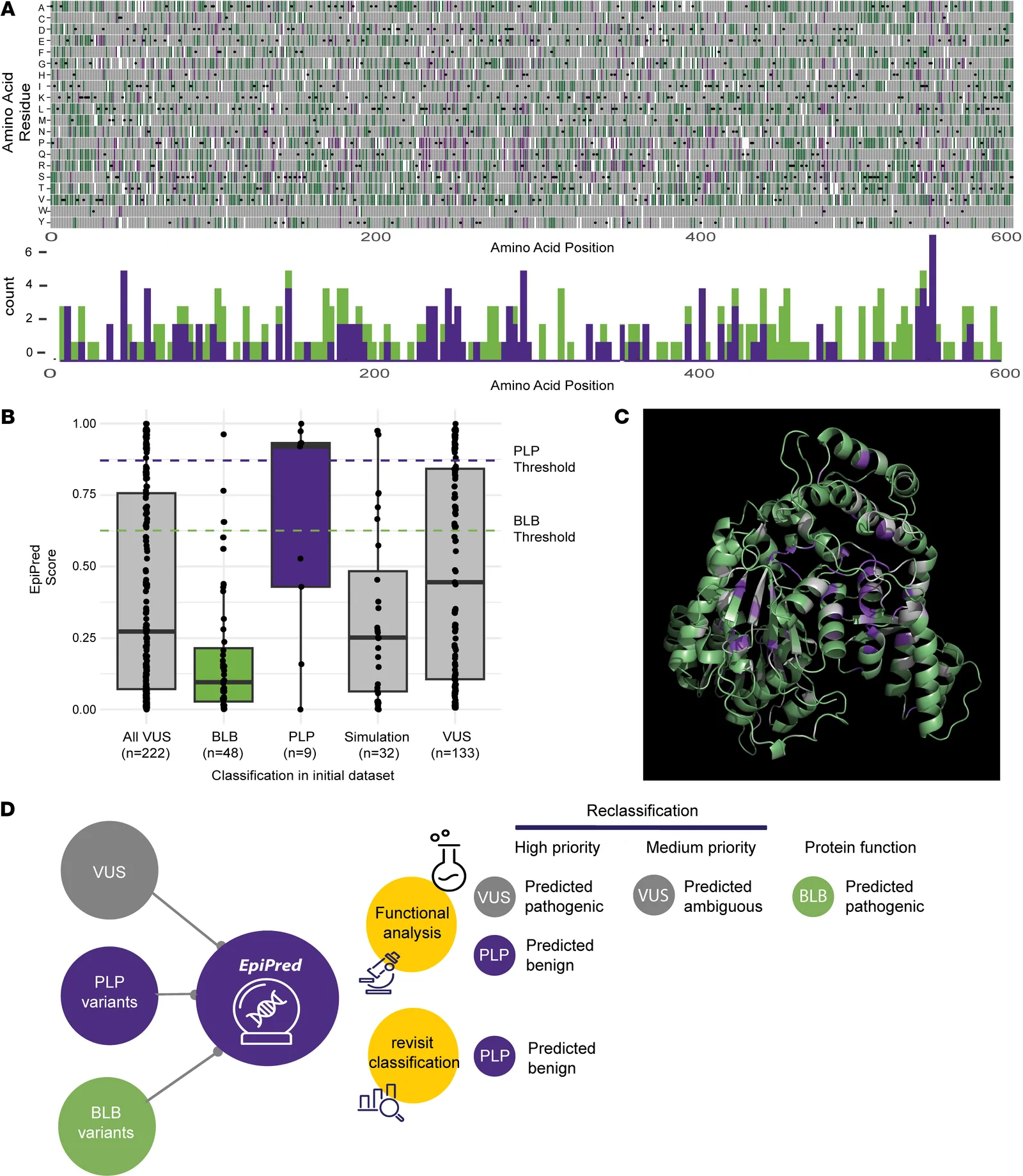
EpiPred-P^PLP^ scores for every possible *STXBP1* missense variant and comparison with published experimental data. (**A**) EpiPred-P^PLP^ variant calls for every possible SNV in the *STXBP1* gene represented as a heatmap (top). Variants called BLB are shown in green and those called PLP are shown in purple. Variants called ambiguous are displayed in grey. Distribution of all known BLB and PLP variants across STXBP1 (bottom) (**B**) Distribution of EpiPred scores for latest version of ClinVar (accessed 8-7-2025) (**C**) EpiPred-P^PLP^ scores for every possible missense variant (mean per residue) were mapped onto the AlphaFold structure using PyMol. (**D**) Potential workflow for deploying EpiPred in reclassification of all classes of variants. For instance, those VUS EpiPred predicted as pathogenic, or PLP variants predicted benign are the highest priority for further characterization to confirm pathogenicity. Conversely, those VUS and PLP variants predicted benign by EpiPred should be reevaluated in terms of the ACMG criteria for classification, as these may not be contributory.

## Version 1 09/17/2026

In-Press Preview

## Version 2 11/02/2026

Electronic publication
