# Supplementary material for: Gene-specific machine learning model EpiPred identifies likely pathogenic variants in the epilepsy-related gene STXBP1
Source: J Clin Invest. 2026 Sep 17;136(21):e207160. doi: 10.1172/JCI207160 (PMC13722379; doi:10.1172/JCI207160)
Supplement: Supplementary file 1 [file jci-136-207160-s168.pdf]

Supplementary material for:

EpiPred: A gene-specific machine learning model for classifying missense variants in the epilepsy-related gene *STXBP1*

Authors: Jeffrey D Calhoun[†1], Chengbing Wang [†2], Carina Biar[1], Jonathan R Gunti [1], John Lee[3], Aaron M Geller[3], Jung H Hong[1], Santiago Schnell[4], Louis T Dang[5], Yu Wang[6], Jack Parent[6] Lori L. Isom[6,7], Michael D Uhler[8,] Heather C. Mefford[9], M. Elizabeth Ross [2†*], Vanessa Aguiar-Pulido[10,2†], Gemma L Carvill[1,11†*]

Affiliations:

[1] Ken and Ruth Davee Department of Neurology, Northwestern Feinberg School of Medicine; Chicago, Illinois, USA

[2] Center for Neurogenetics, Feil Family Brain and Mind Research Institute, Weill Cornell Medicine; New York, NY, USA

[3] Northwestern IT Research Computing and Data Services; Evanston, IL, USA

[4] Departments of Mathematics, Biochemistry & Cell Biology and Biomedical Data Science, Dartmouth College; Hanover, NH , USA

[5] Department of Pediatrics, University of Michigan; Ann Arbor, MI, USA

[6] Department of Neurology, University of Michigan; Ann Arbor, MI, USA

[7] Department of Pharmacology, University of Michigan; Ann Arbor, MI, USA

[8] Department of Biological Chemistry and Molecular Neuroscience Institute, University of Michigan; Ann Arbor, MI, USA

[9] Center for Pediatric Neurological Disease Research, St. Jude Children's Research Hospital; Memphis, TN, USA

[10] University of Miami, Miami, FL, USA

[11] Department of Pharmacology, Northwestern Feinberg School of Medicine; Chicago, Illinois, USA

*Corresponding author. Email: Gemma L. Carvill (gemma.carvill@northwestern.edu) and M. Elizabeth Ross (mer2005@med.cornell.edu)

† These authors contributed equally to this work

Table of Contents

EpiPred: A gene-specific machine learning model for classifying missense variants in the epilepsy-related gene STXBP1 .... *1*

List of supplementary tables **4**

Supplementary Table 1: All truth set STXBP1 variants annotated with EpiPred model output **4**

Supplementary Table 2: All STXBP1 missense VUS annotated with EpiPred model output **4**

Supplementary Table 3: Feature lists for EpiPred model training and benchmarking **4**

Supplementary Table 4: Comprehensive model metrics for each annotated feature and collection of global VEPs across full truth set **4**

Supplementary Table 5: Pearson correlation table to demonstrate correlation of each functional assay with each annotated feature **4**

Supplemental Table 6: All holdout set STXBP1 variants annotated with EpiPred model output **4**

Supplemental Table 7: Comprehensive model metrics for each annotated feature across a holdout set. **4**

Supplemental Table 8: PLP Variants called in the Truth and Holdout dataset predicted to be BLB by EpiPred **4**

Supplemental Table 9: Comprehensive model metrics for each annotated feature across holdout set with updated Holdout Set minus variants reclassified to VUS (n=9). **4**

Supplemental Table 10: All possible missense variants in STXBP1 along with metrics and EpiPred predictions **4**

Supplemental Table 11: Published functional data for a subset of missense variants **4**

Supplemental Table 12: Pearson and spearman correlation of PRESR model with functional data. EpiPred correlation also shown for comparison. **4**

Supplemental Table 13: All VUS in current version of ClinVar (date accessed 8-4-2025) **4**

Supplemental Table 14: Summary of EpiPred properties of all VUS in current version of ClinVar (date accessed 8-4-2025) **4**

Supplementary Figure 1: AUROC for the EpiPred truth set **5**

Supplementary Figure 2: Specificity, sensitivity, and accuracy of EpiPred and other VEPs on the full truth set **5**

Supplementary Figure 3: SHAP distributions of feature importance for the truth set (top). **6**

BLB missense variants that are predicted pathogenic by EpiPred **7**

Supplementary Figure 4: Variants from the truth set that are incorrectly called by EpiPred **10**

Supplementary Figure 5: Location of conflicting variants in the 3D representation of STXBP1 11

Supplementary Figure 6: Correlation plot (Pearson) across functional readouts, combined scores and EpiPred 12

Supplementary Figure 7: Individual correlation plots (Pearson) across functional readouts 15

Supplementary Figure 8: Correlation plot (Pearson) for functional data, EpiPred, and all features used in the EpiPred model as well as common global VEPs 16

Supplementary Figure 9: ROC for the EpiPred holdout dataset 17

Supplementary Figure 10: Specificity, sensitivity, and accuracy of EpiPred and other VEPs on the full holdout set with and without the PLP variants (n=9) reclassified as VUS. 17

Supplementary Figure 11: Feature distribution for EpiPred in the holdout set. 18

Supplementary Figure 12: EpiPred and global VEP comparisons 19

Supplementary Figure 13: Most VEPs/features have better classification performance on truth set as compared to holdout set. 20

Supplementary Figure 14: An aggregate functional score from multiple publications, and correlation with EpiPred-PPLP scores 21

Supplementary Figure 15: ROC curve and ROC_{AUC} for EpiPRED compared with PRESR. 22

Supplementary Figure 16: PRESR and EpiPRED are highly correlated. 23

List of supplementary tables

Supplementary Table 1: All truth set STXBP1 variants annotated with EpiPred model output

Supplementary Table 2: All STXBP1 missense VUS annotated with EpiPred model output

Supplementary Table 3: Feature lists for EpiPred model training and benchmarking

Supplementary Table 4: Comprehensive model metrics for each annotated feature and collection of global VEPs across full truth set

Supplementary Table 5: Pearson correlation table to demonstrate correlation of each functional assay with each annotated feature

Supplemental Table 6: All holdout set STXBP1 variants annotated with EpiPred model output

Supplemental Table 7: Comprehensive model metrics for each annotated feature across a holdout set.

Supplemental Table 8: PLP Variants called in the Truth and Holdout dataset predicted to be BLB by EpiPred

Supplemental Table 9: Comprehensive model metrics for each annotated feature across holdout set with updated Holdout Set minus variants reclassified to VUS (n=9).

Supplemental Table 10: All possible missense variants in STXBP1 along with metrics and EpiPred predictions

Supplemental Table 11: Published functional data for a subset of missense variants

Supplemental Table 12: Pearson and spearman correlation of PRESR model with functional data. EpiPred correlation also shown for comparison.

Supplemental Table 13: All VUS in current version of ClinVar (date accessed 8-4-2025)

Supplemental Table 14: Summary of EpiPred properties of all VUS in current version of ClinVar (date accessed 8-4-2025)

Please see separate excel document

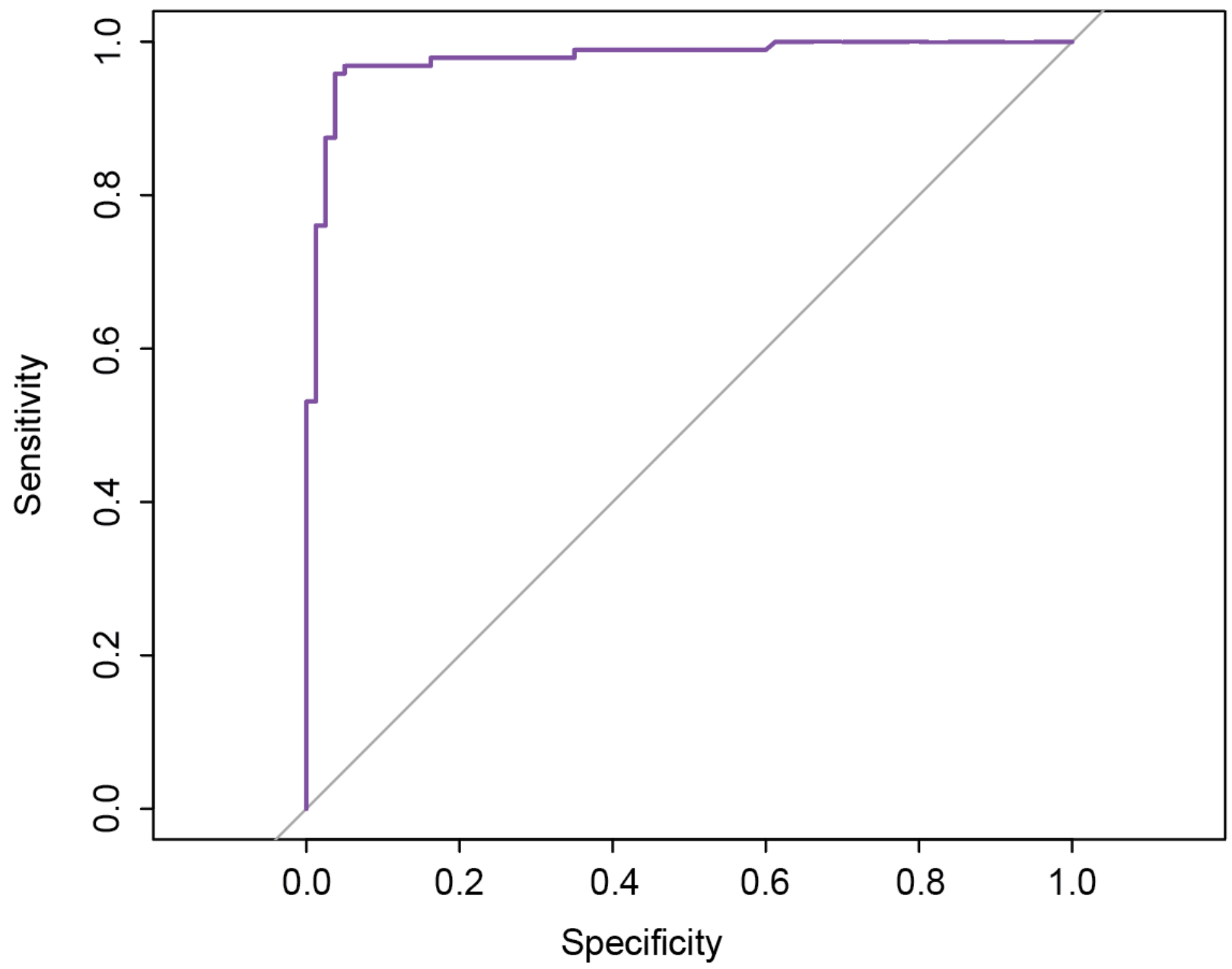

Supplementary Figure 1: AUROC for the EpiPred truth set

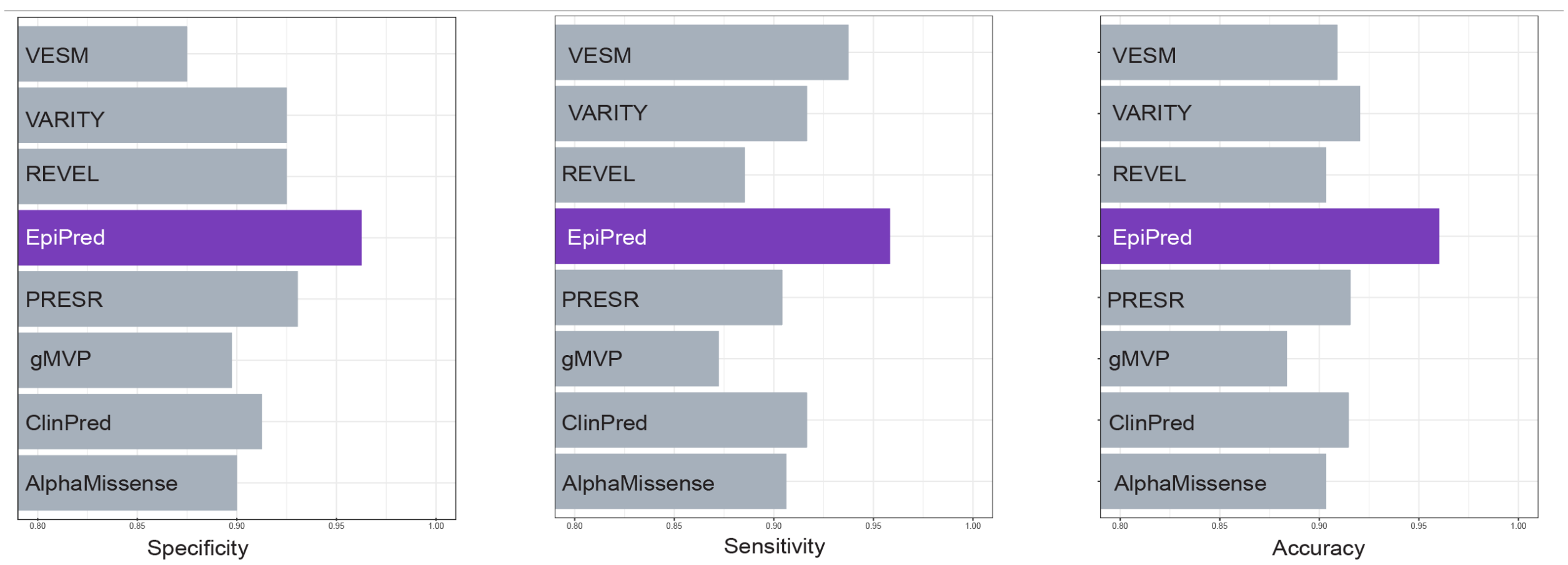

Supplementary Figure 2: Specificity, sensitivity, and accuracy of EpiPred and other VEPs on the full truth set

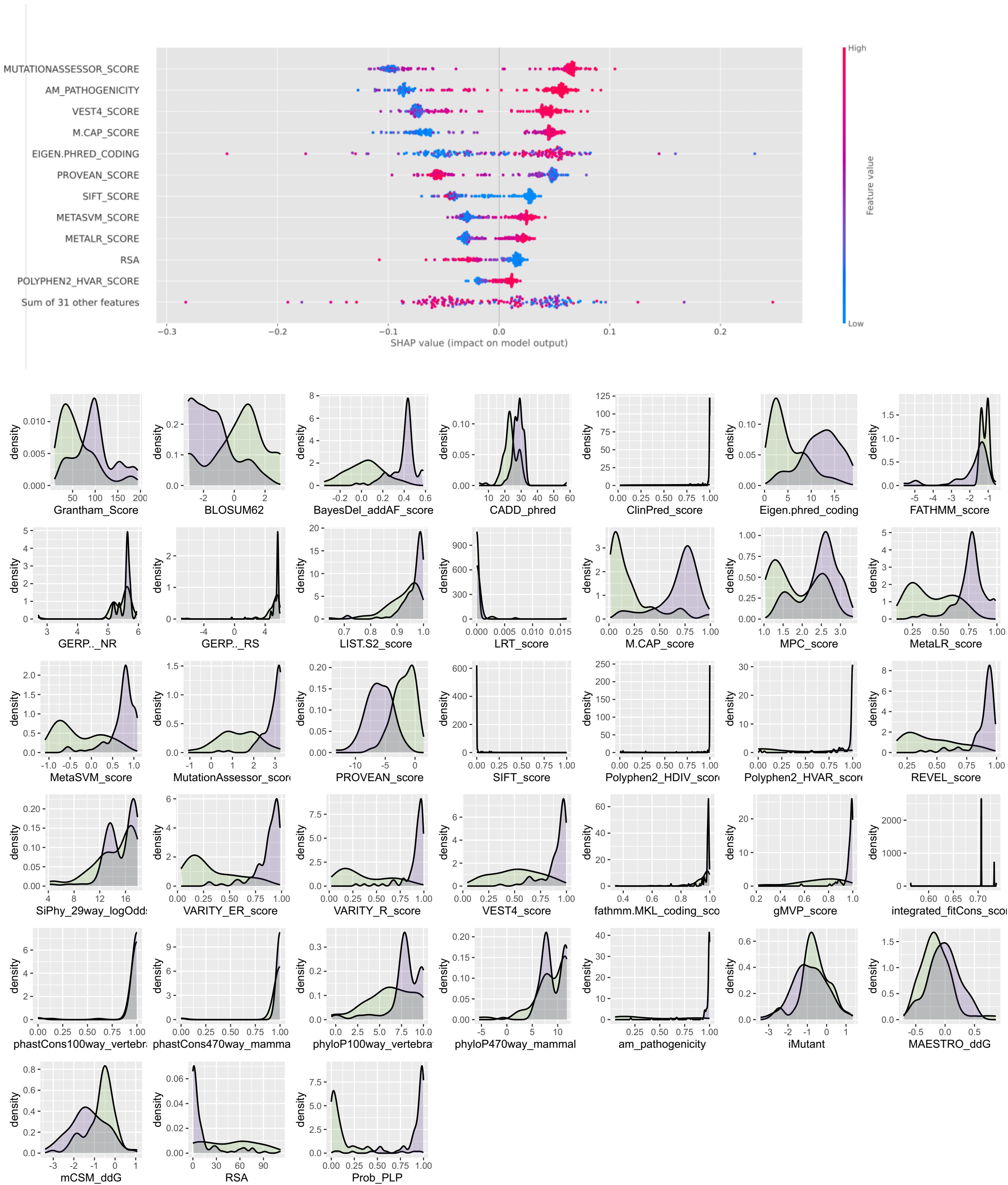

Supplementary Figure 3: SHAP distributions of feature importance for the truth set (top). The top features leveraged in EpiPred are the global VEPs. With Mutation Assessor being the strongest contributor, residue solvent accessibility (RSA) also ranks highly. Feature distribution for EpiPredSTXBP1 in the truth set (bottom). Green is class 0 (Truth set BLB); purple is class 1 (Truth set PLP). For unabbreviated feature names, see Table S3.

BLB missense variants that are predicted pathogenic by EpiPred

Phe153Ser (gnomAD allele count 2)

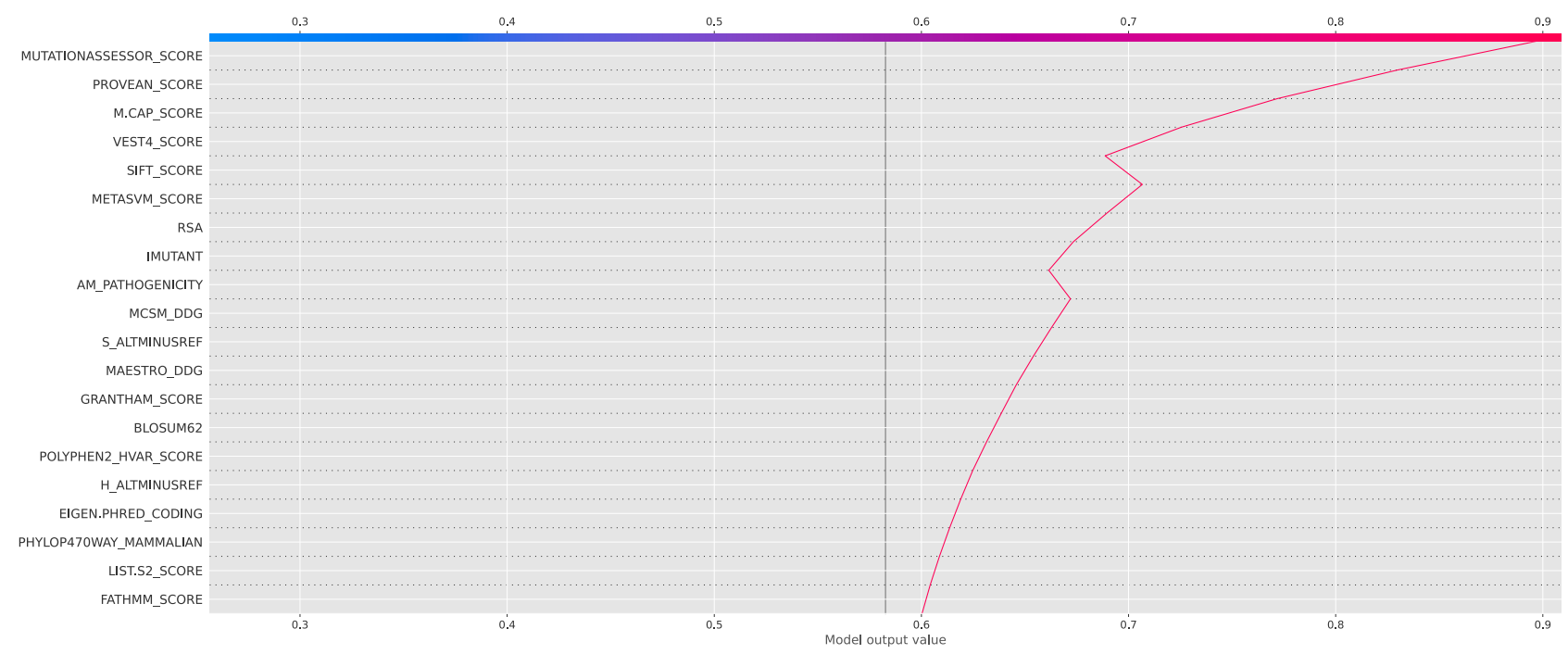

Arg536His (gnomAD allele count 30)

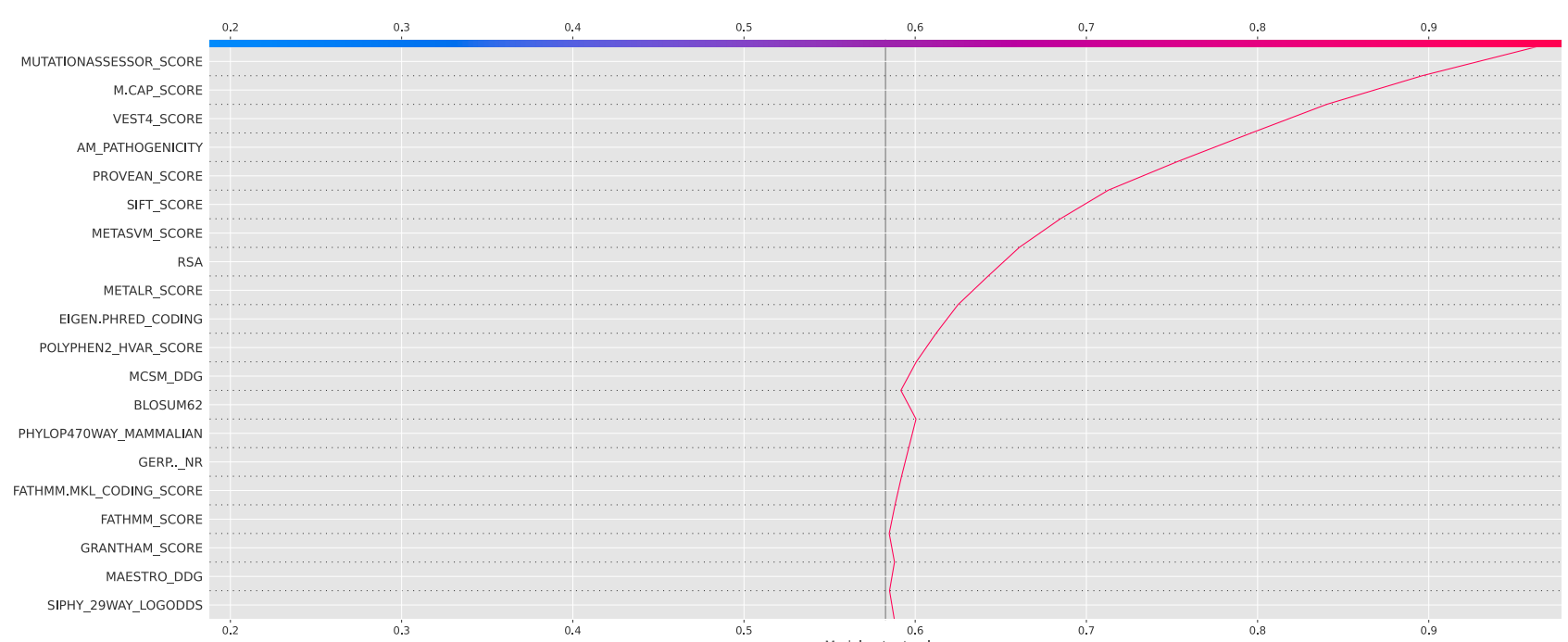

PLP missense variants that are predicted benign by EpiPred

Gly193Val

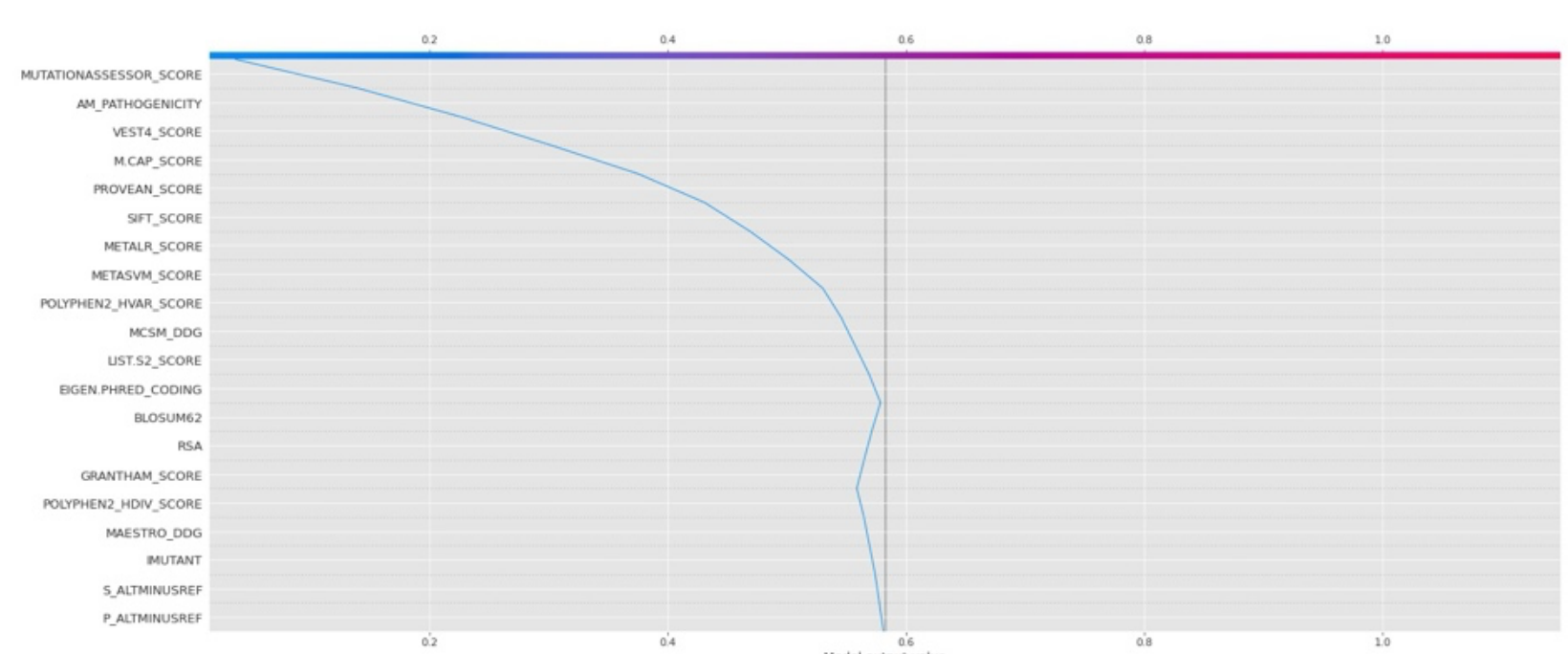

Asn548Asp

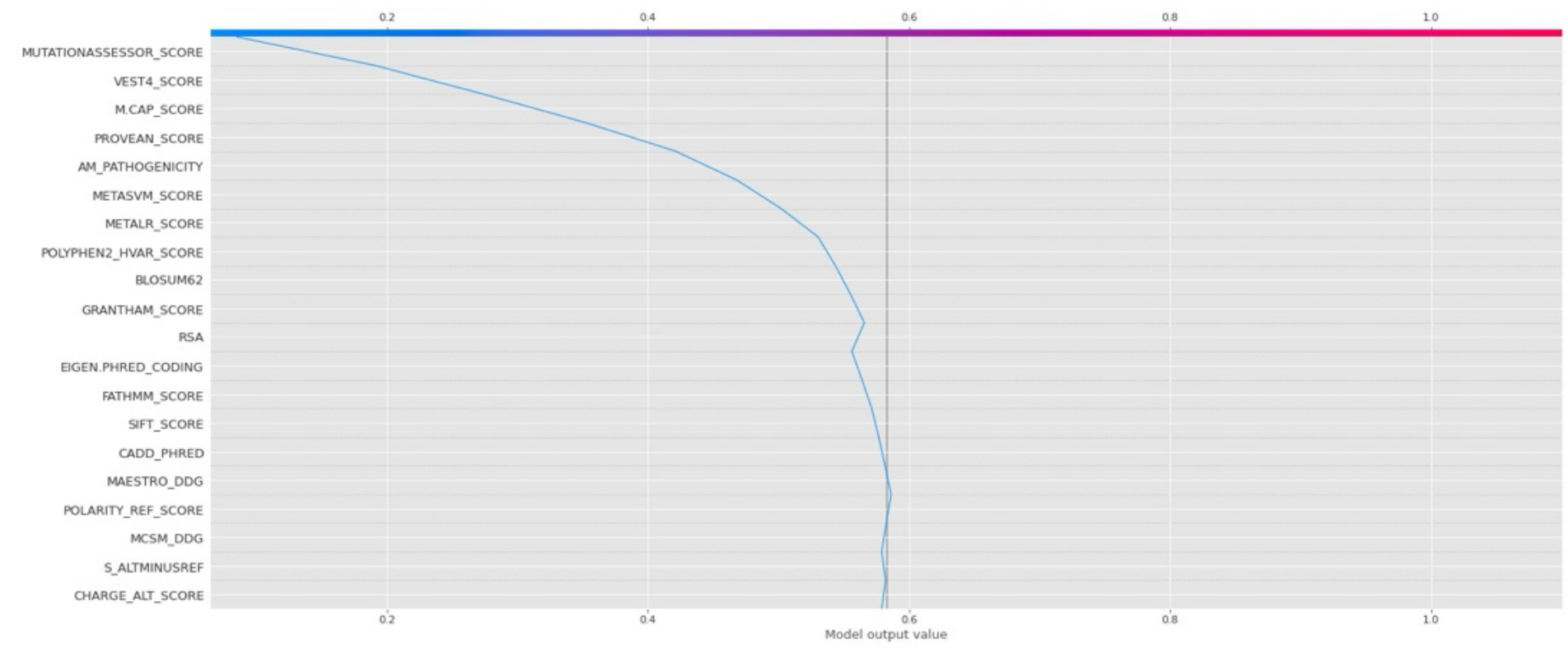

Ala517Ser

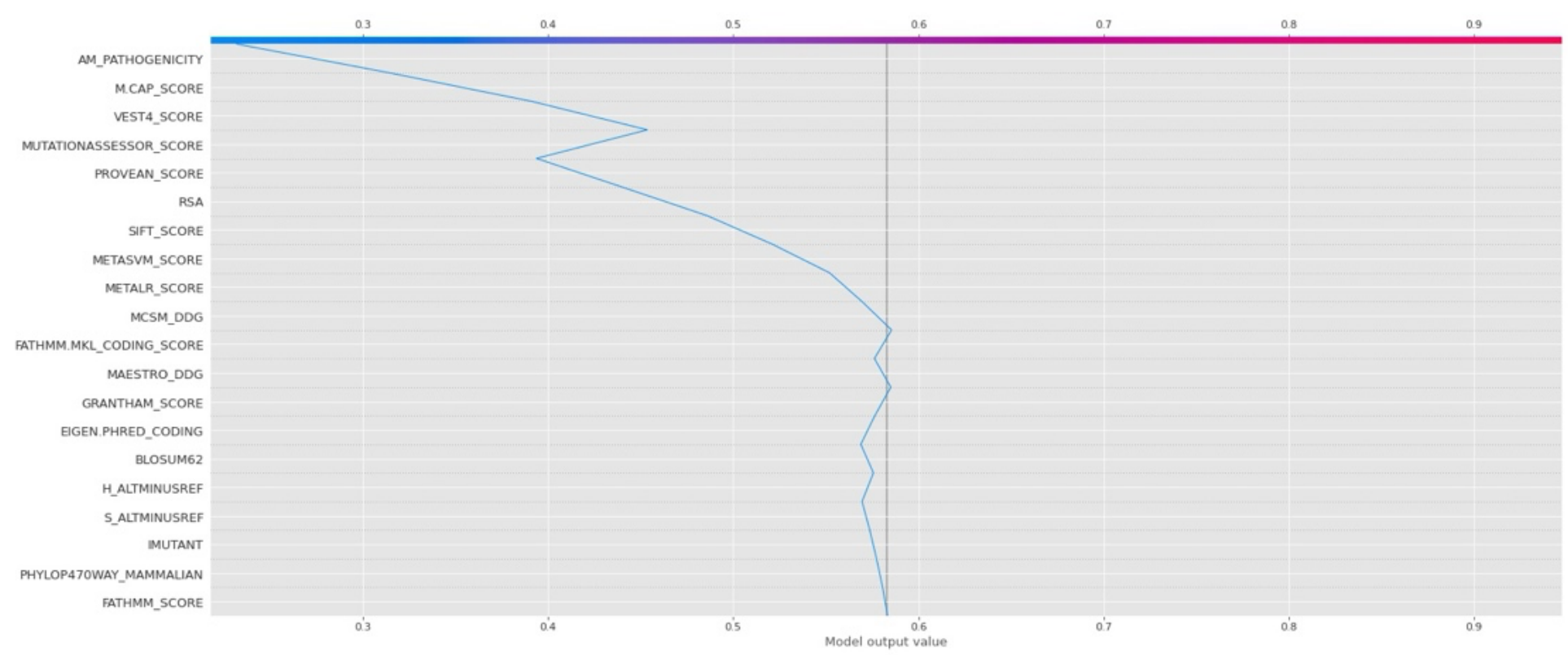

Ser80Pro

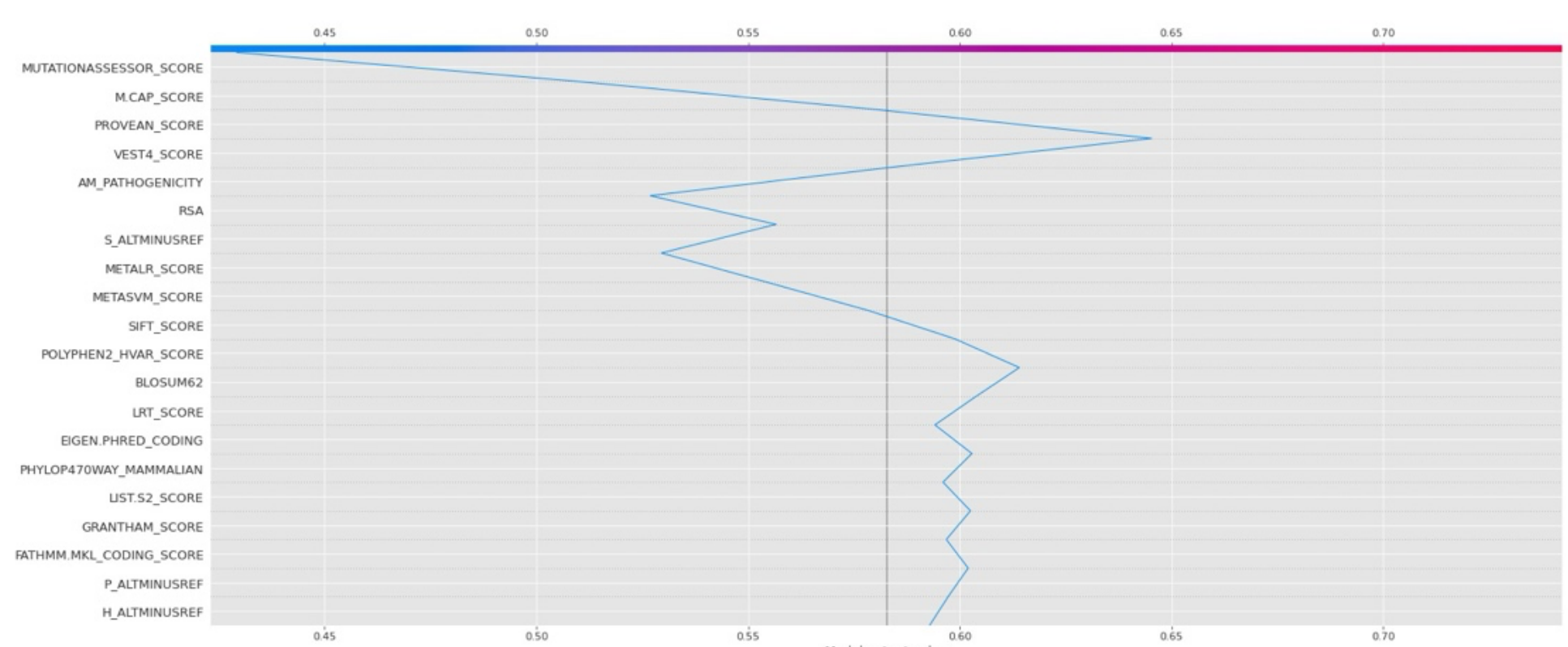

Glu487Asp

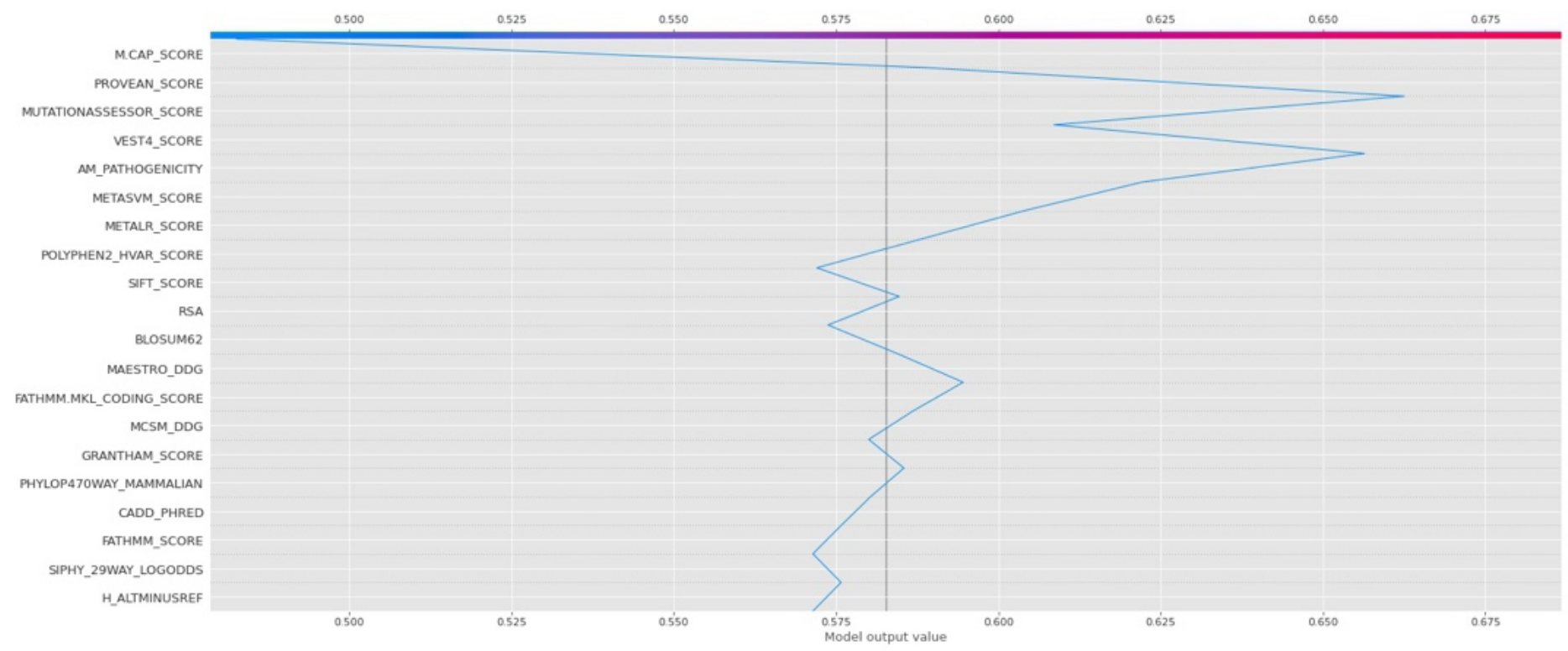

Thr129Pro

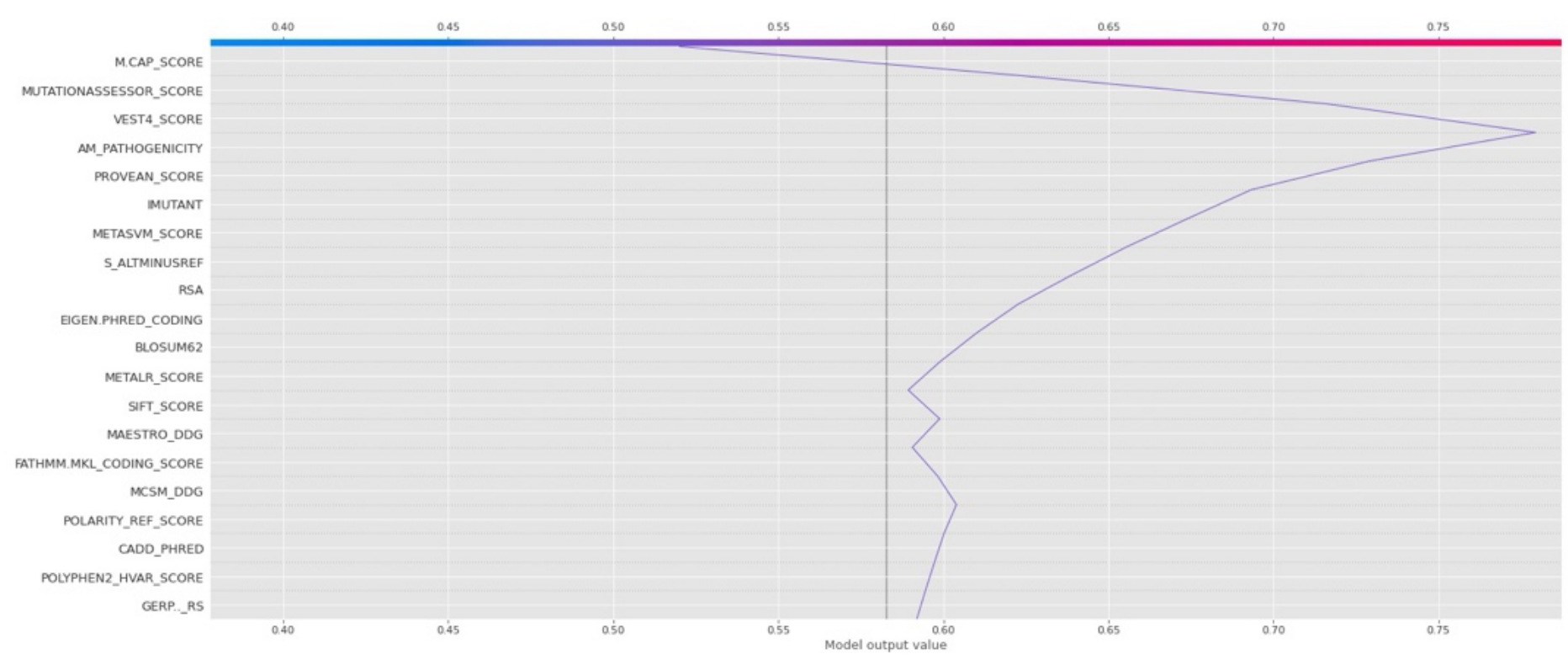

Lys7Glu

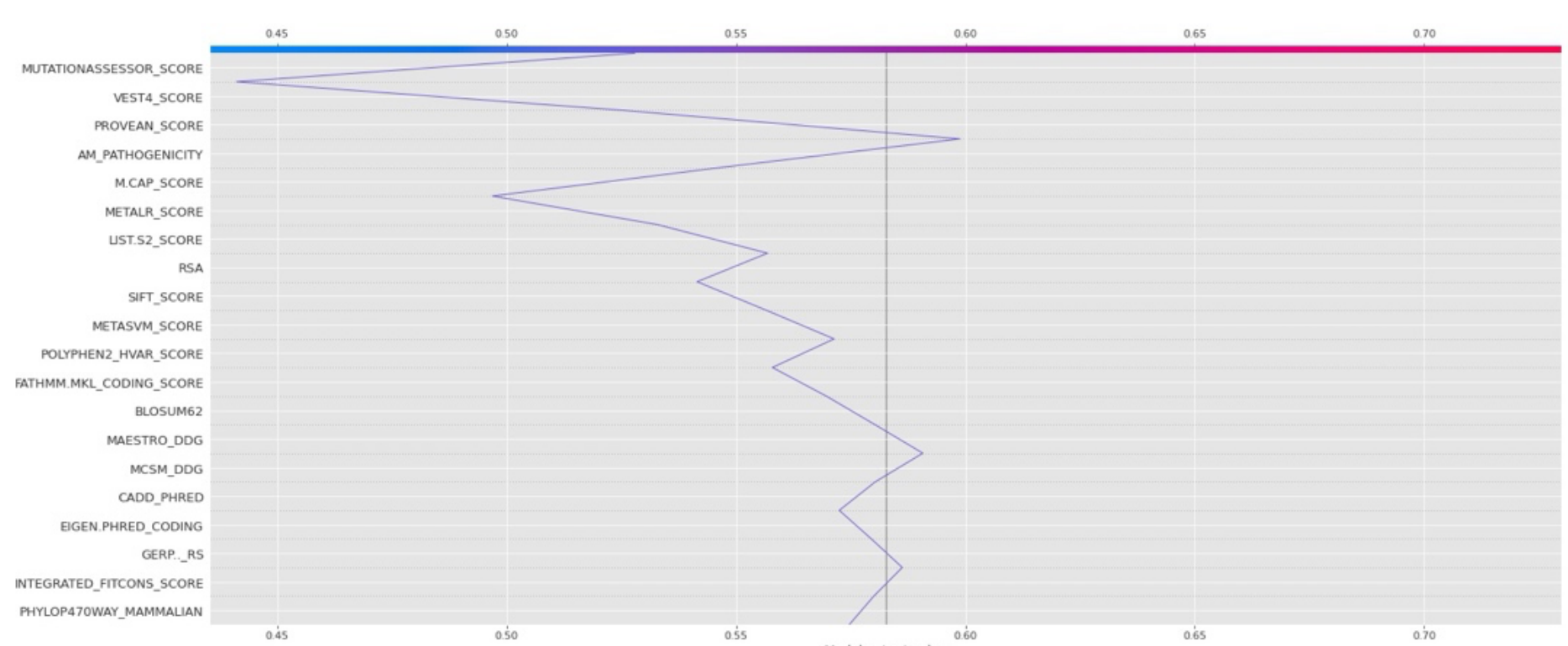

Asp284Tyr

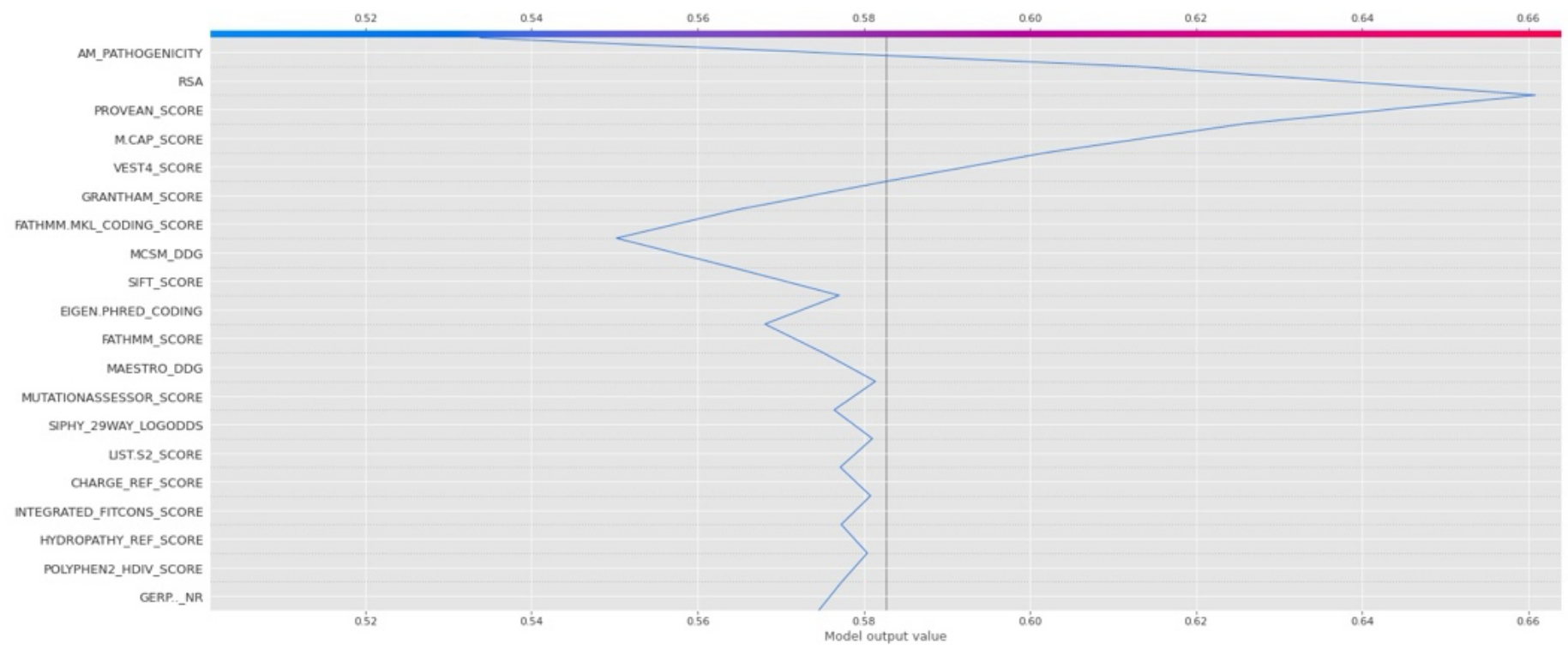

Thr574Pro

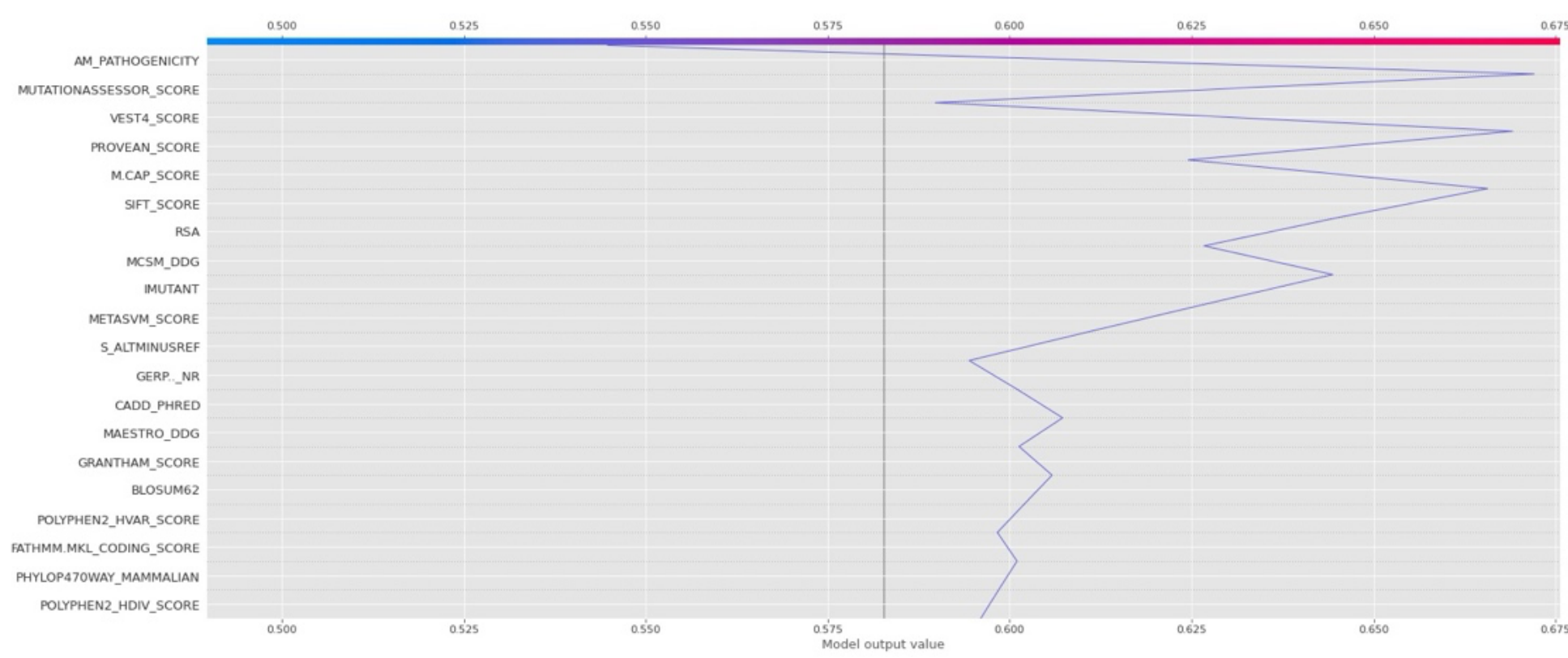

Thr361Ile

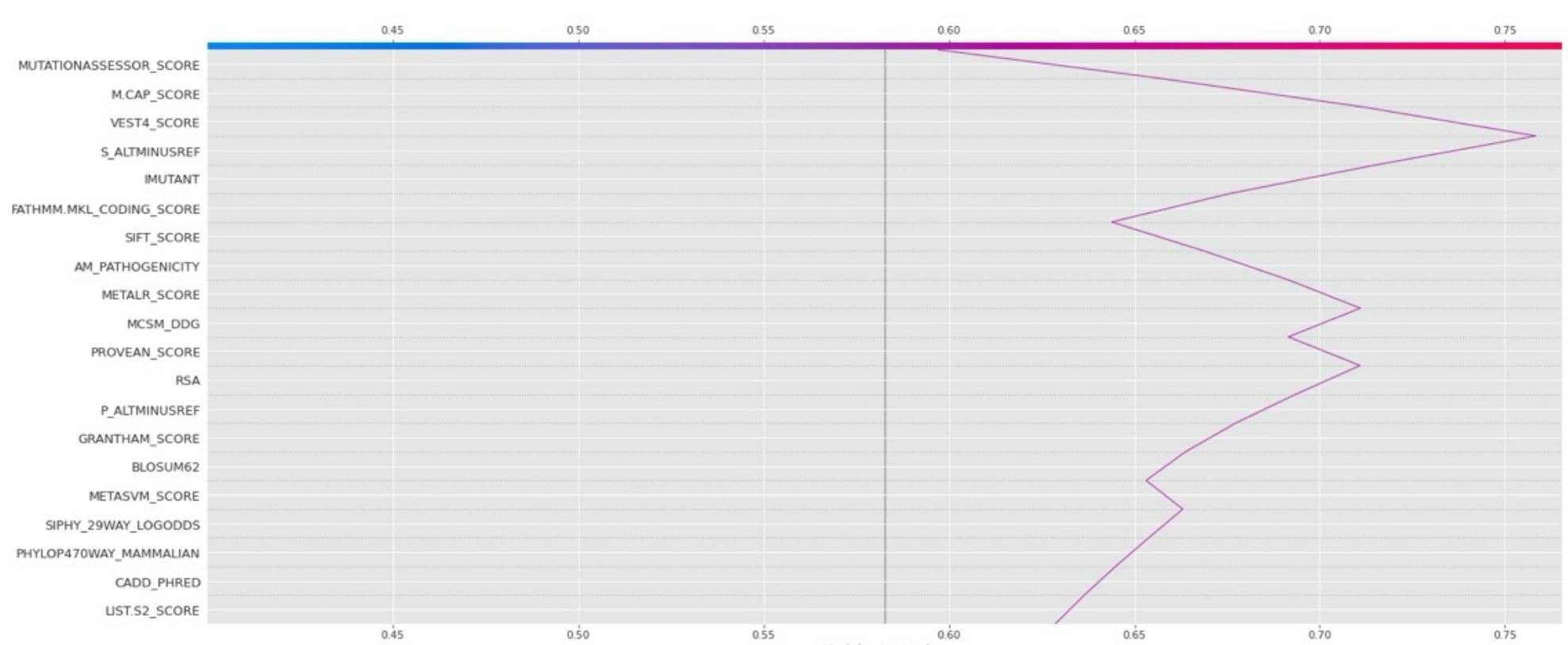

Supplementary Figure 4: Variants from the truth set that are incorrectly called by EpiPred

Conflicting variants (n=15) in the truth set called the incorrect class by EpiPred-STXBP1				
**Variant**	**Class**	**EpiPred score (prediction)**	**SpliceAi score (max)***	**Variant at same position**
K7E	PLP	0.53 (BLB)	0.01	No
S80P	PLP	0.43 (BLB)	0	No
T129P	PLP	0.52 (BLB)	0	No
G193V	PLP	0.04 (BLB)	0.49 (donor loss)	No
D284Y	PLP	0.53 (BLB)	0.01	No
T361I	PLP	0.60 (BLB)	0.01	No
E487D	PLP	0.48 (BLB)	**0.78 (donor loss)**	No
A517S	PLP	0.23 (BLB)	0	Yes, A517T, BLB
N548D	PLP	0.08 (BLB)	0	No, but also N548I in the holdout set PLP called BLB by EpiPred (0.26)
T574P	PLP	0.54 (BLB)	0.05	No
H103D	BLB	0.97 (PLP)	NA	Yes, H103P, PLP
F153S	BLB	0.90 (PLP)	NA	No
Y266C	BLB	0.95 (PLP)	NA	No
R536C	BLB	0.95 (PLP)	NA	No
**R536H**	**BLB**	**0.96** (PLP)	NA	**No**

***** SpliceAi gives four scores for acceptor/donor gain/loss we show only highest score, 0.8 indicates a site predicted likely to disrupt splicing

Variants in red classified as VUS in most recent version of ClinVar

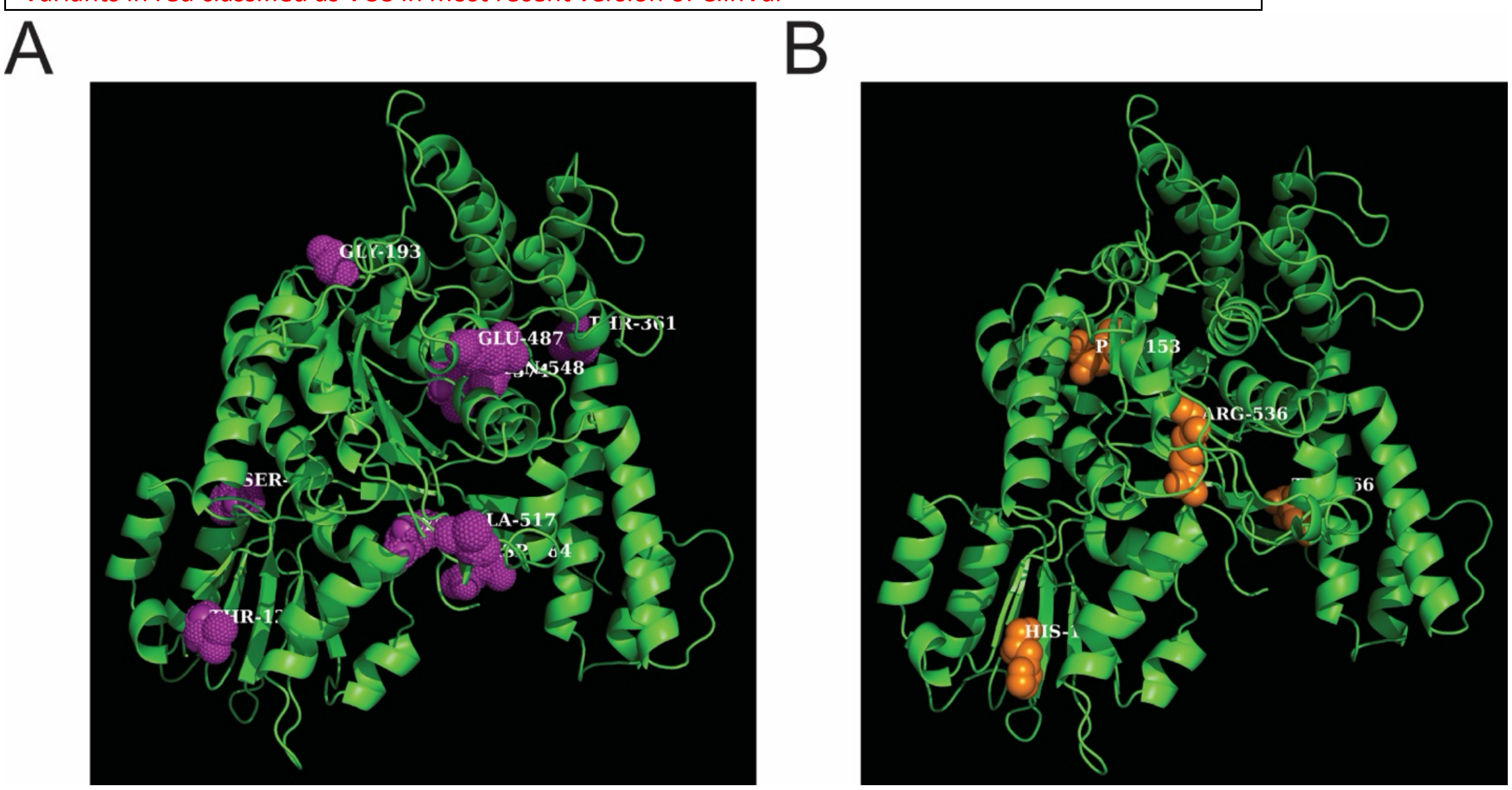

Supplementary Figure 5: Location of conflicting variants in the 3D representation of STXBP1

The 10 PLP variants (left in purple) and 5 BLB variants (right in orange) that are incorrectly predicted by EpiPred and other global variant effect predictors, i.e. where the known ACMG class and EpiPred do not match.

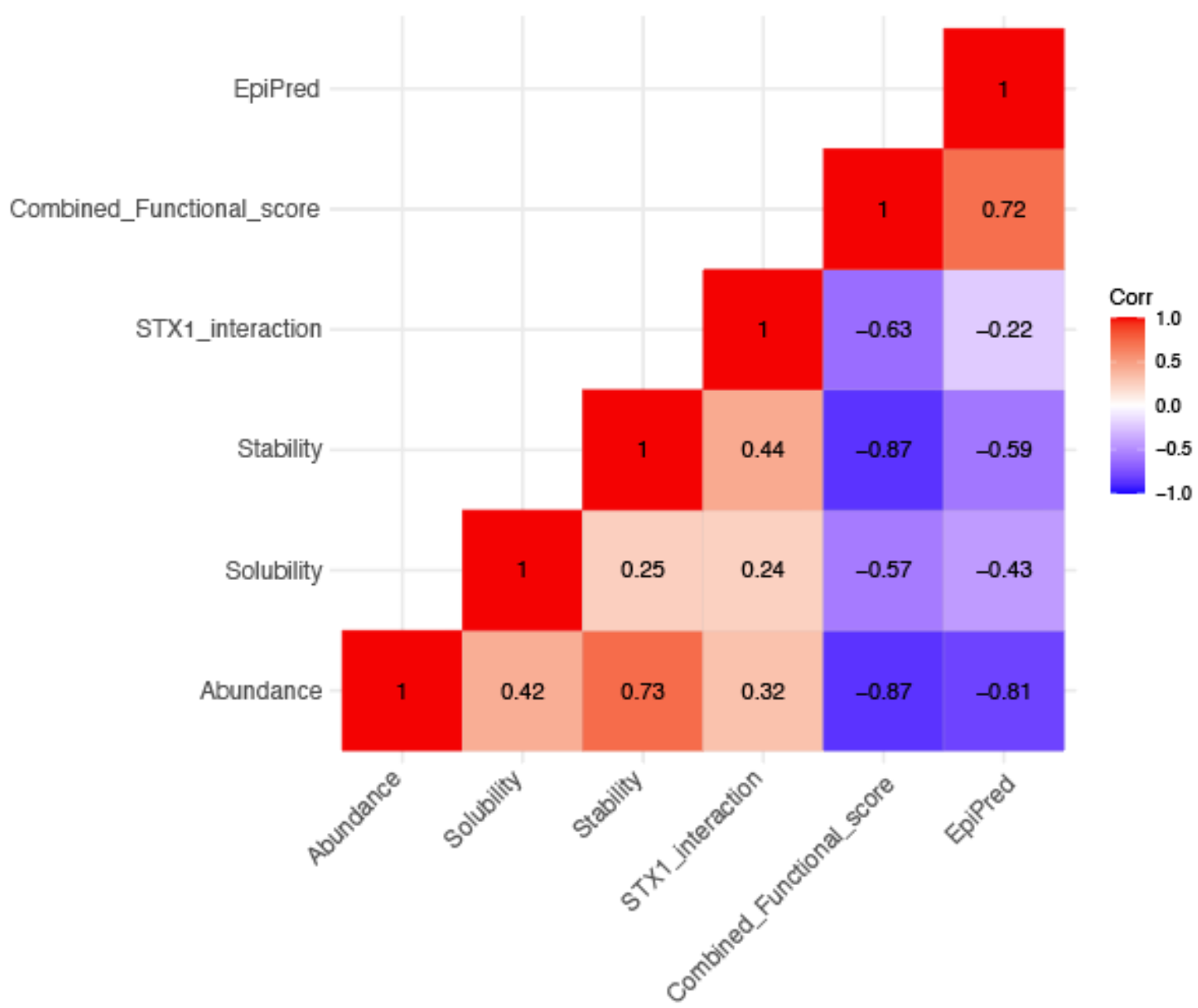

Supplementary Figure 6: Correlation plot (Pearson) across functional readouts, combined scores and EpiPred

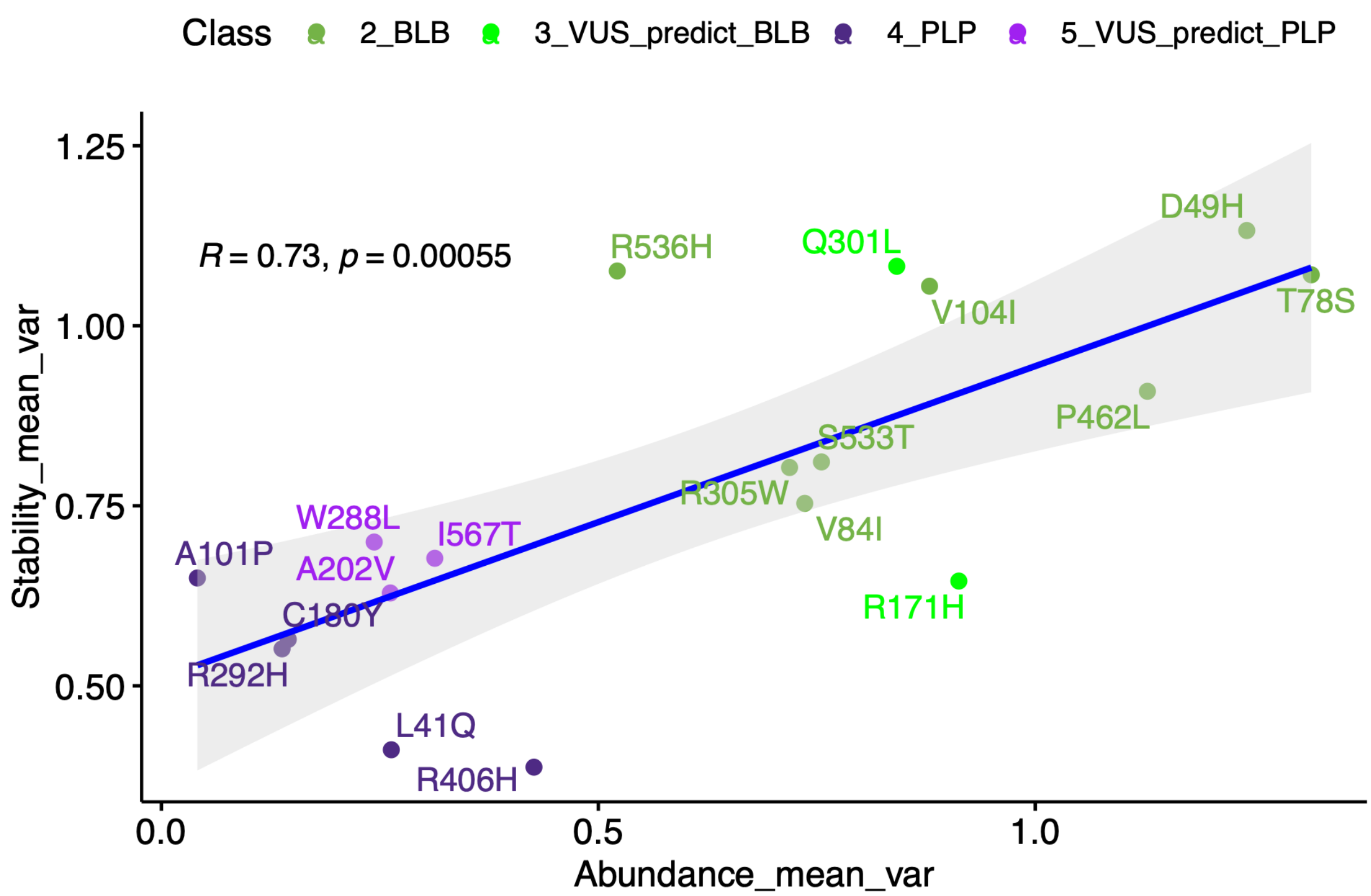
Class
2_BLB
3_VUS_predict_BLB
4_PLP
5_VUS_predict_PLP
R = 0.73, p = 0.00055
D49H
R536H
Q301L
V104I
T78S
P462L
S533T
R305W
V84I
W288L
I567T
A101P
A202V
C180Y
R171H
R292H
L41Q
R406H
Stability_mean_var
Abundance_mean_var
1.25
1.00
0.75
0.50
0.0
0.5
1.0

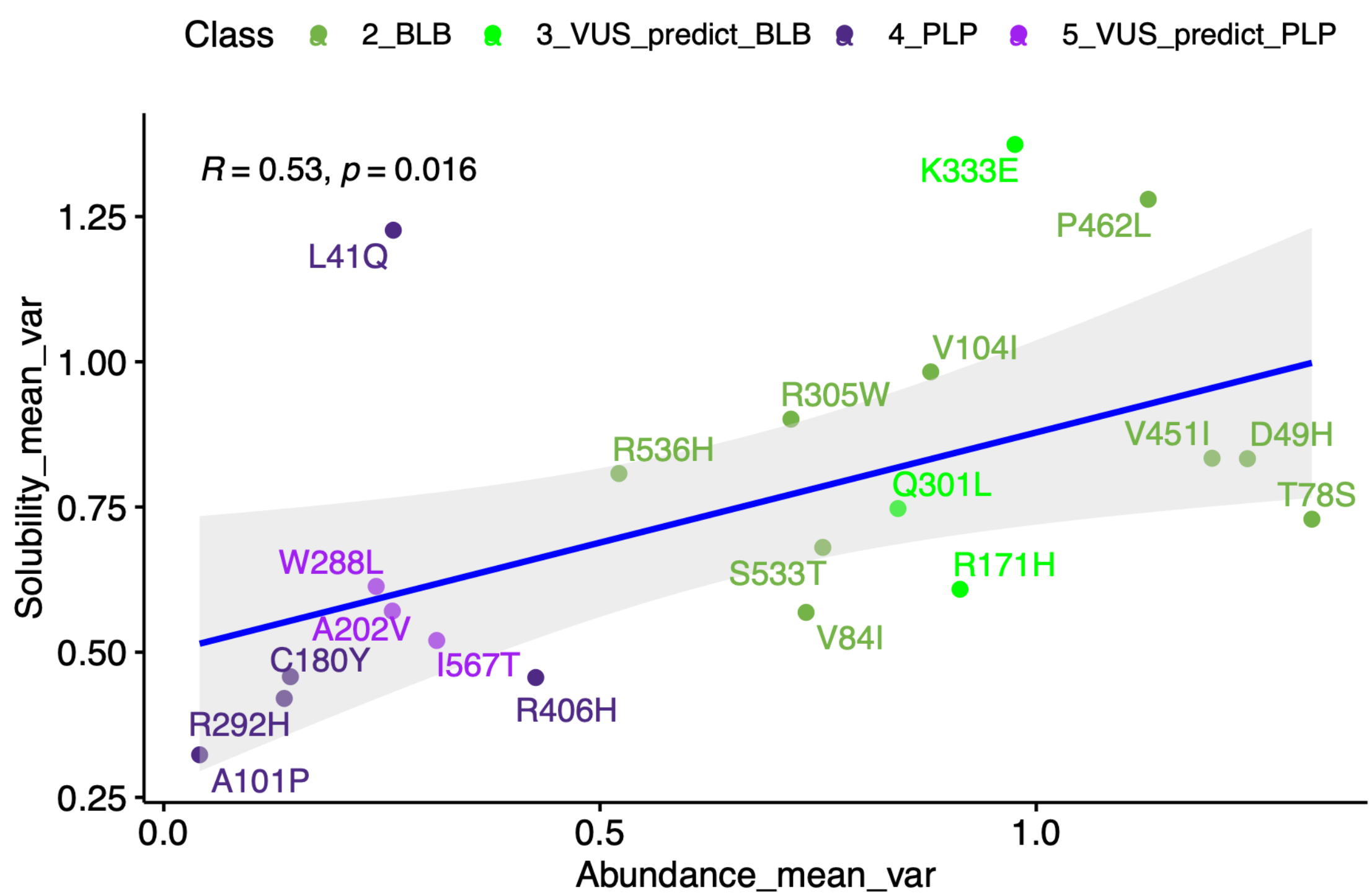
Class
2_BLB
3_VUS_predict_BLB
4_PLP
5_VUS_predict_PLP
R = 0.53, p = 0.016
K333E
P462L
L41Q
V104I
R305W
V451I
D49H
R536H
Q301L
T78S
W288L
S533T
R171H
A202V
V84I
C180Y
I567T
R406H
R292H
A101P
Solubility_mean_var
Abundance_mean_var
1.25
1.00
0.75
0.50
0.25
0.0
0.5
1.0

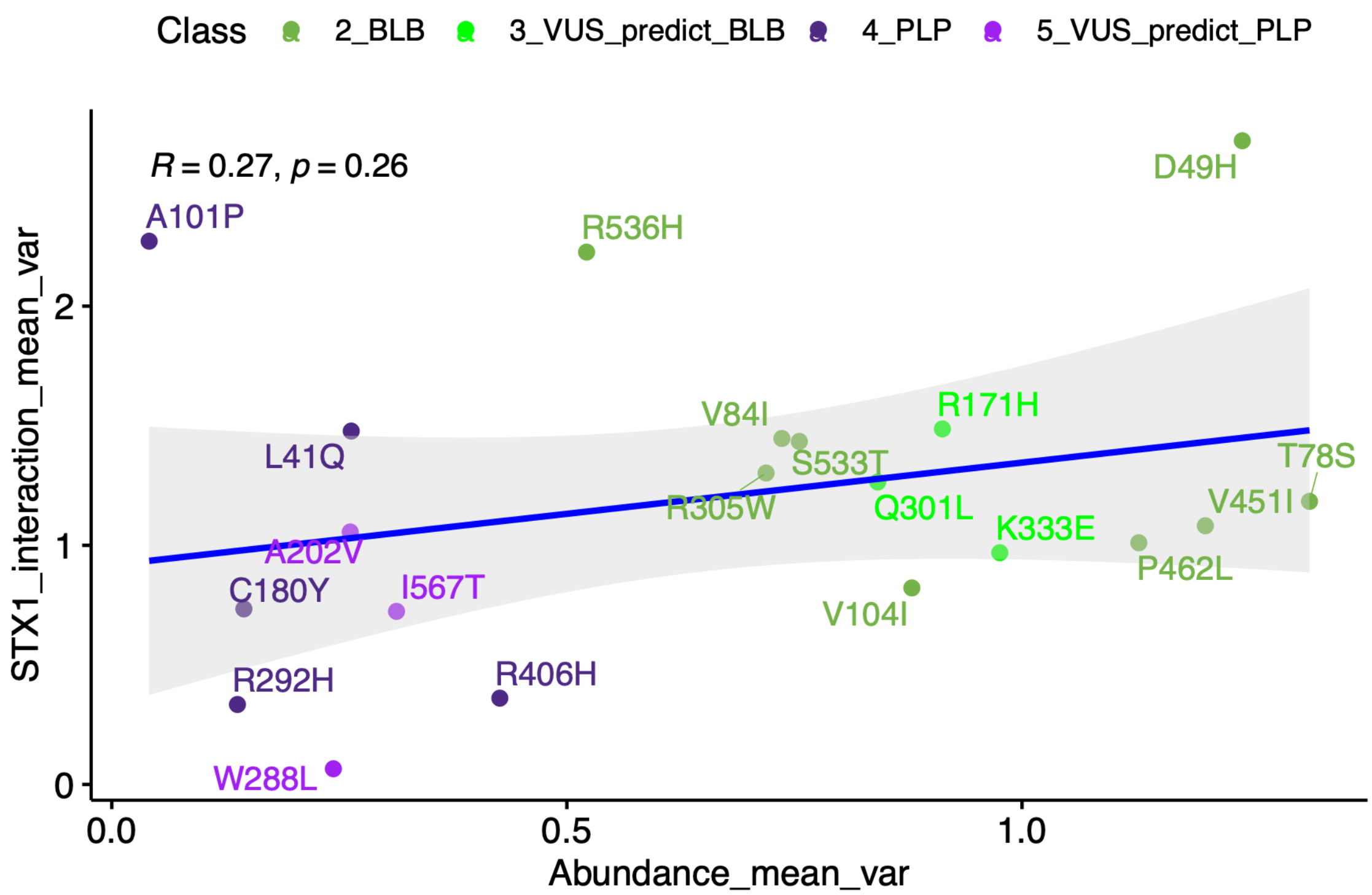
Class 2_BLB 3_VUS_predict_BLB 4_PLP 5_VUS_predict_PLP
R = 0.27, p = 0.26
STX1_interaction_mean_var
Abundance_mean_var
D49H
A101P
R536H
V84I
R171H
L41Q
S533T
T78S
R305W
Q301L
V451I
K333E
A202V
P462L
C180Y
I567T
V104I
R292H
R406H
W288L

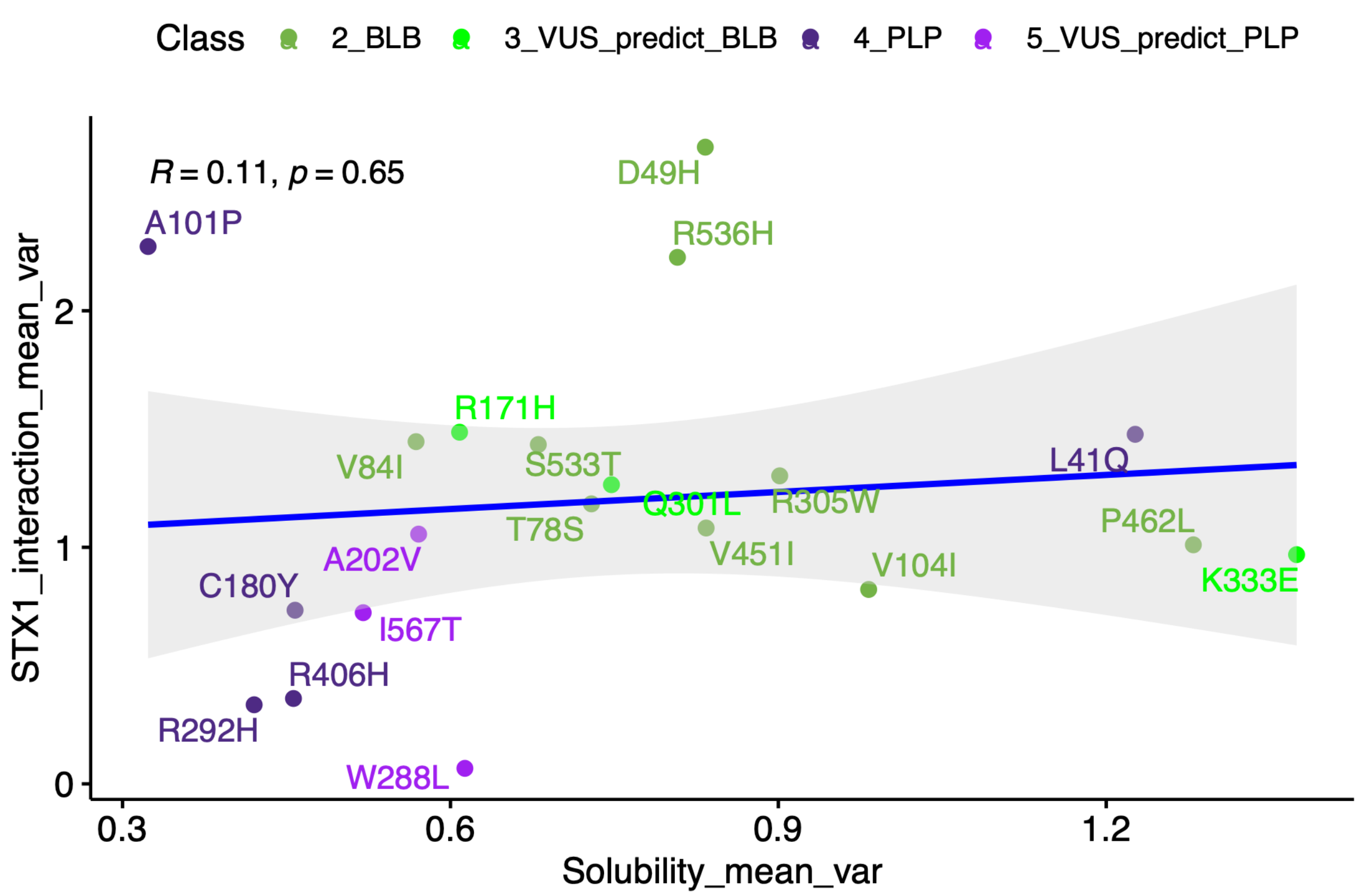
Class 2_BLB 3_VUS_predict_BLB 4_PLP 5_VUS_predict_PLP
R = 0.11, p = 0.65
STX1_interaction_mean_var
Solubility_mean_var
D49H
A101P
R536H
R171H
V84I
S533T
L41Q
Q301L
R305W
T78S
P462L
V451I
V104I
A202V
K333E
C180Y
I567T
R406H
R292H
W288L

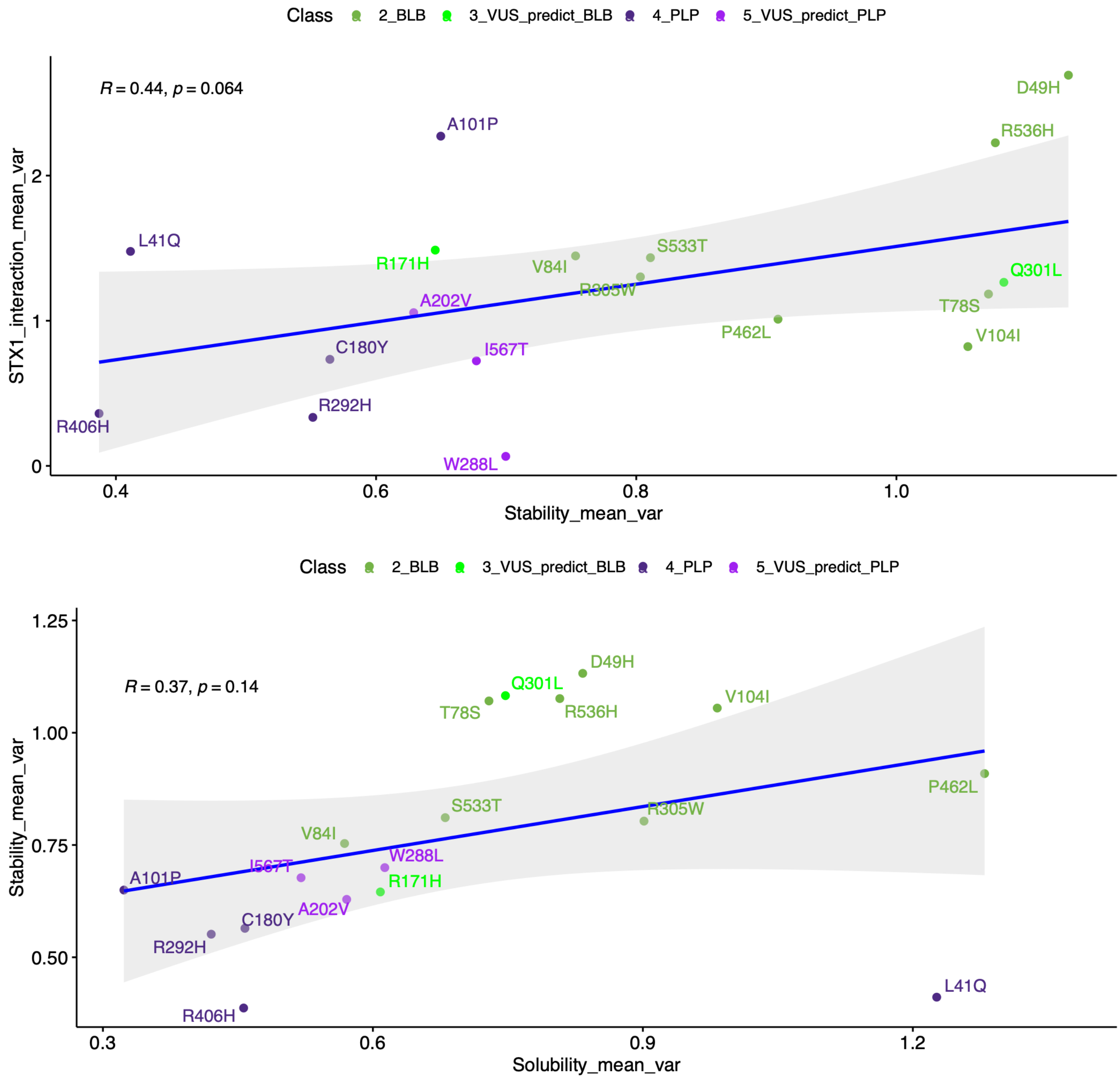

Supplementary Figure 7: Individual correlation plots (Pearson) across functional readouts

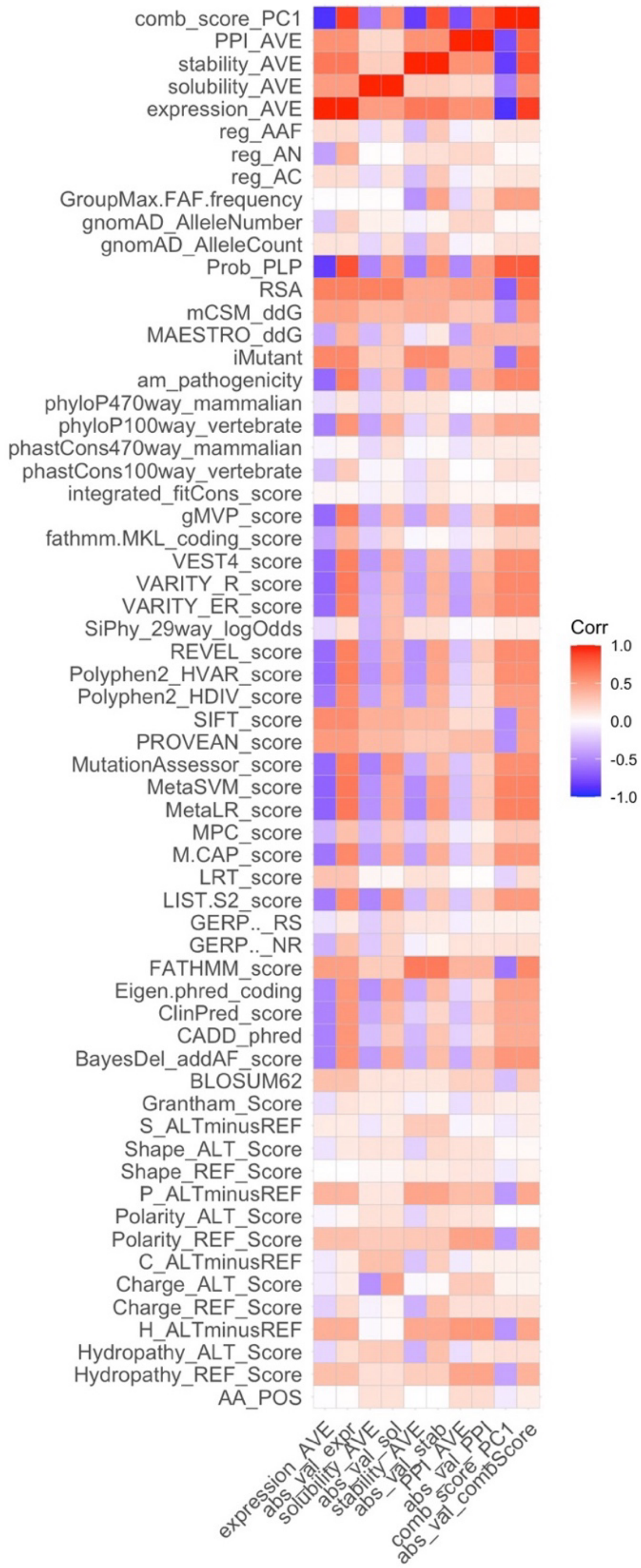

Supplementary Figure 8: Correlation plot (Pearson) for functional data, EpiPred, and all features used in the EpiPred model as well as common global VEPs

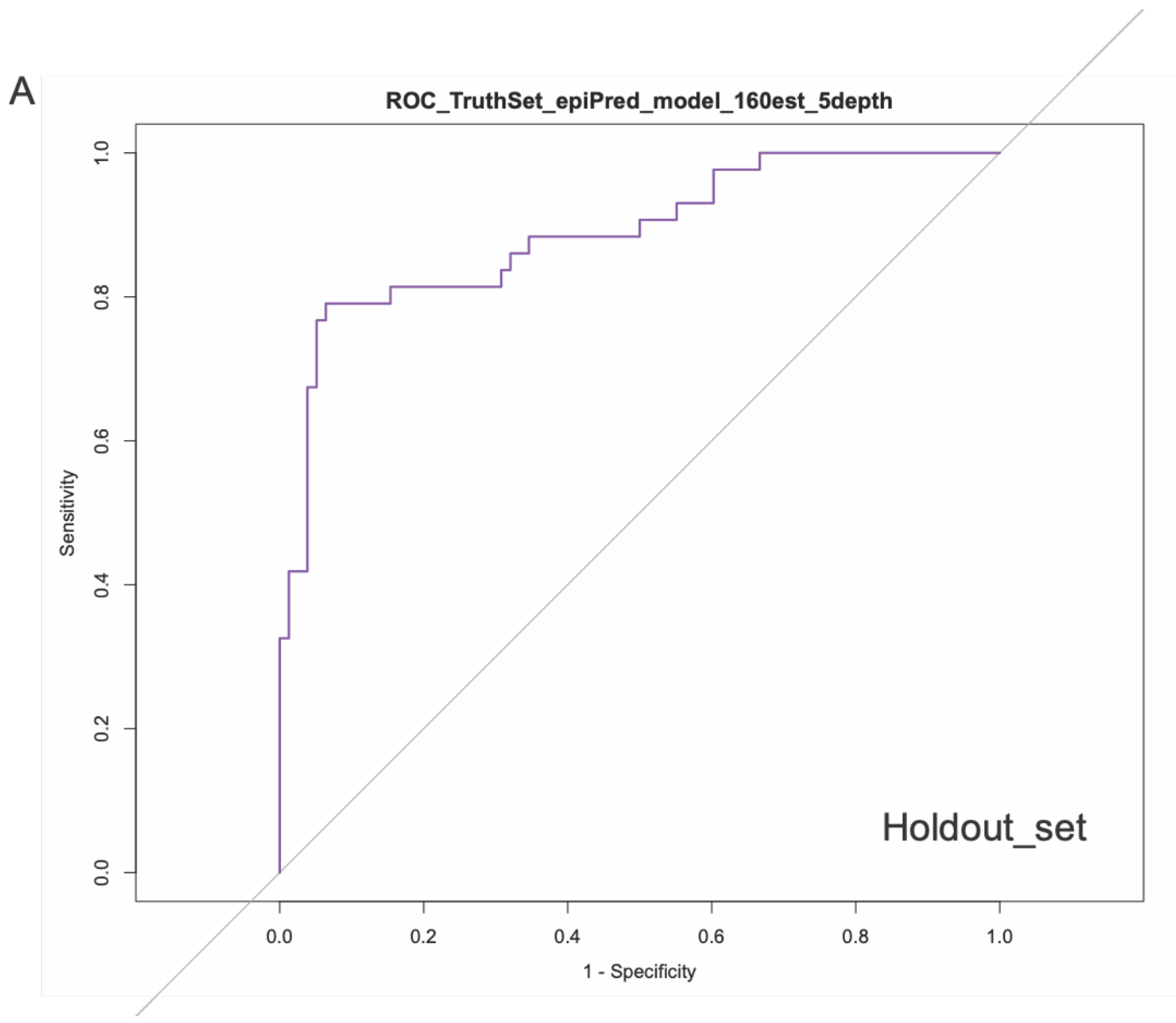

Supplementary Figure 9: ROC for the EpiPred holdout dataset

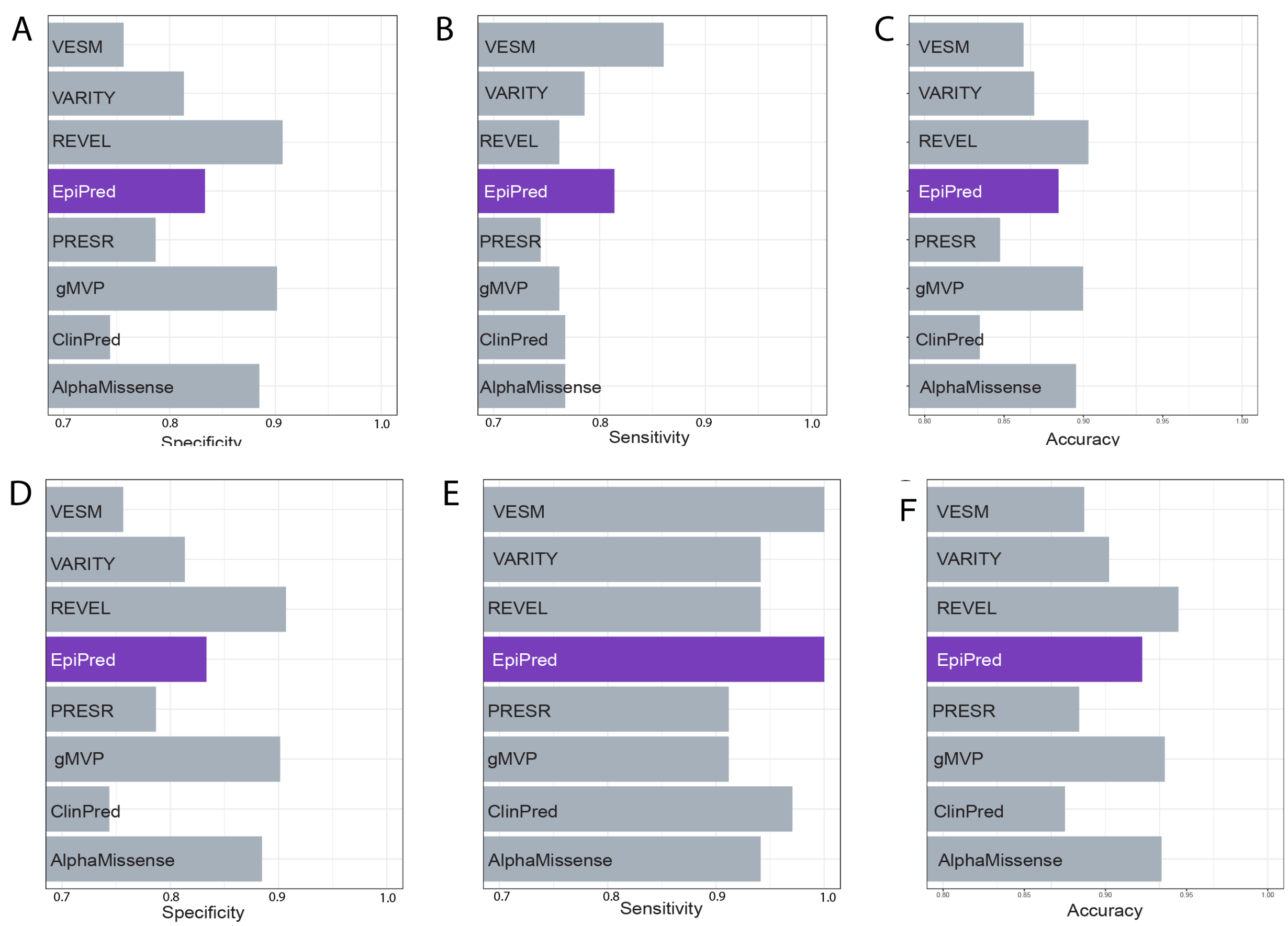

Supplementary Figure 10: Specificity, sensitivity, and accuracy of EpiPred and other VEPs on the full holdout set with and without the PLP variants (n=9) reclassified as VUS. (A-C) Metrics on full holdout set. (**D-F**) Metrics on holdout set excluding reclassified variants.

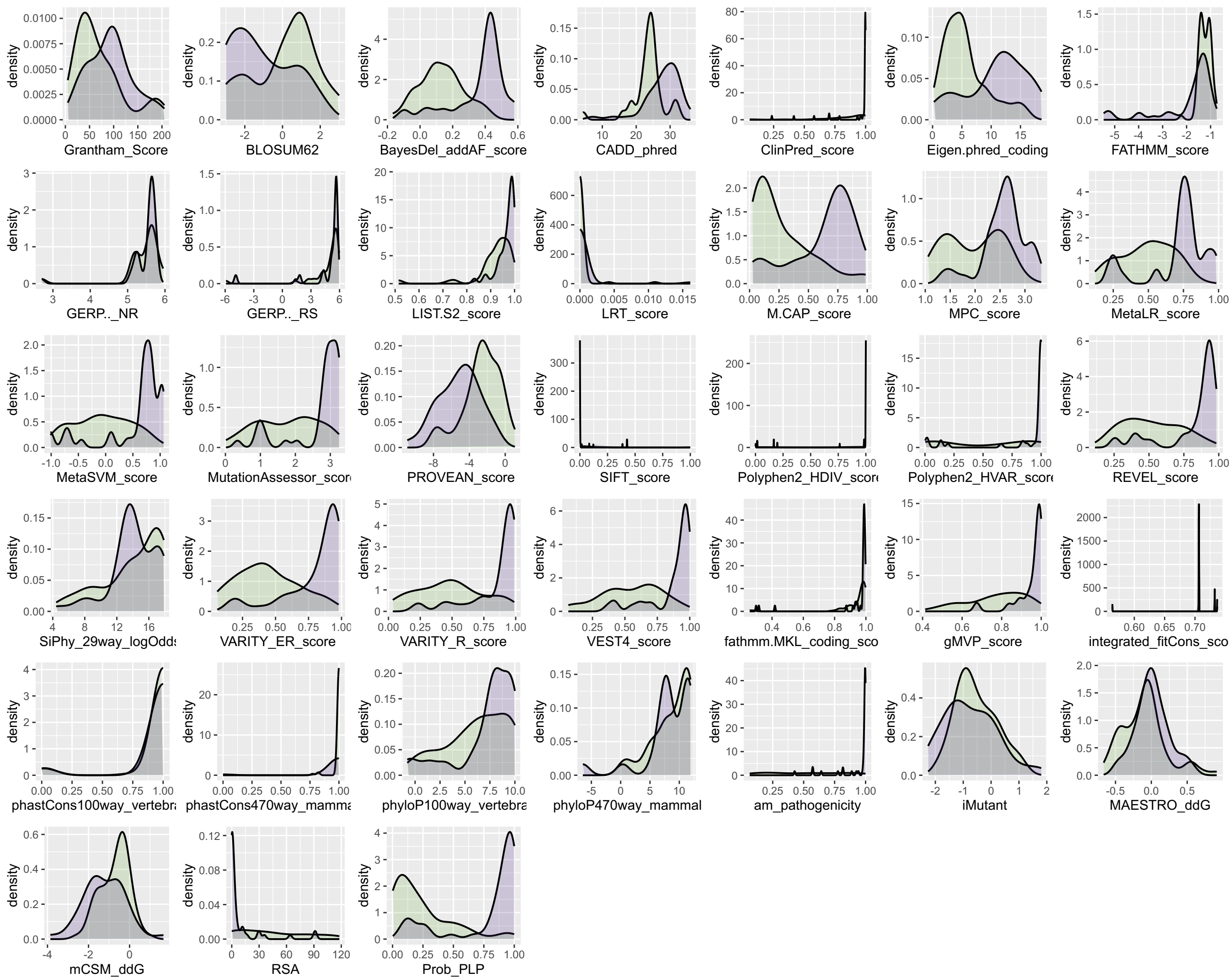

Supplementary Figure 11: Feature distribution for EpiPred in the holdout set. Green is class 5 (Holdout set BLB); purple is class 6 (Holdout set PLP). For unabbreviated feature names, please see **Table S3.**

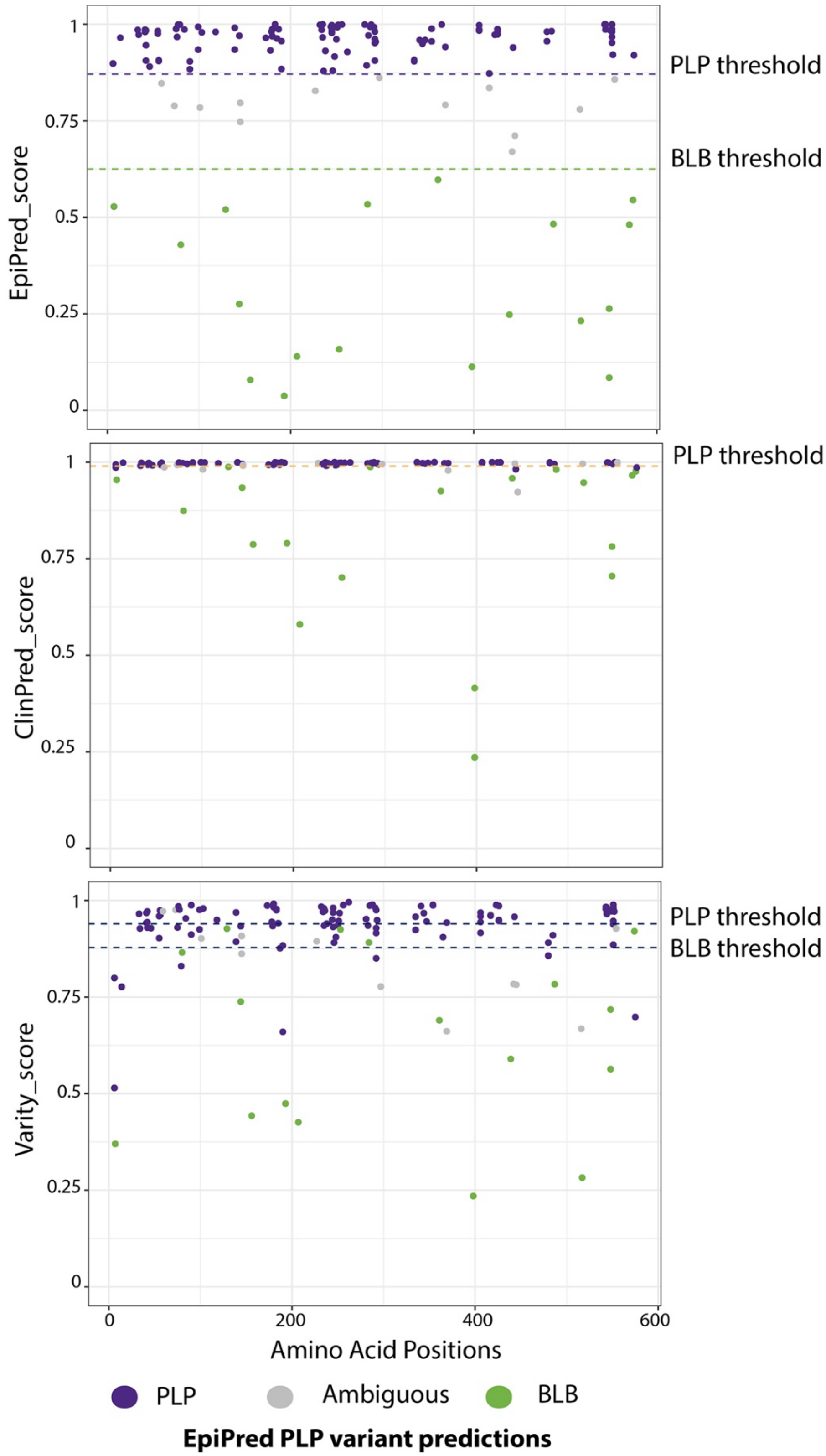

Supplementary Figure 12: EpiPred and global VEP comparisons

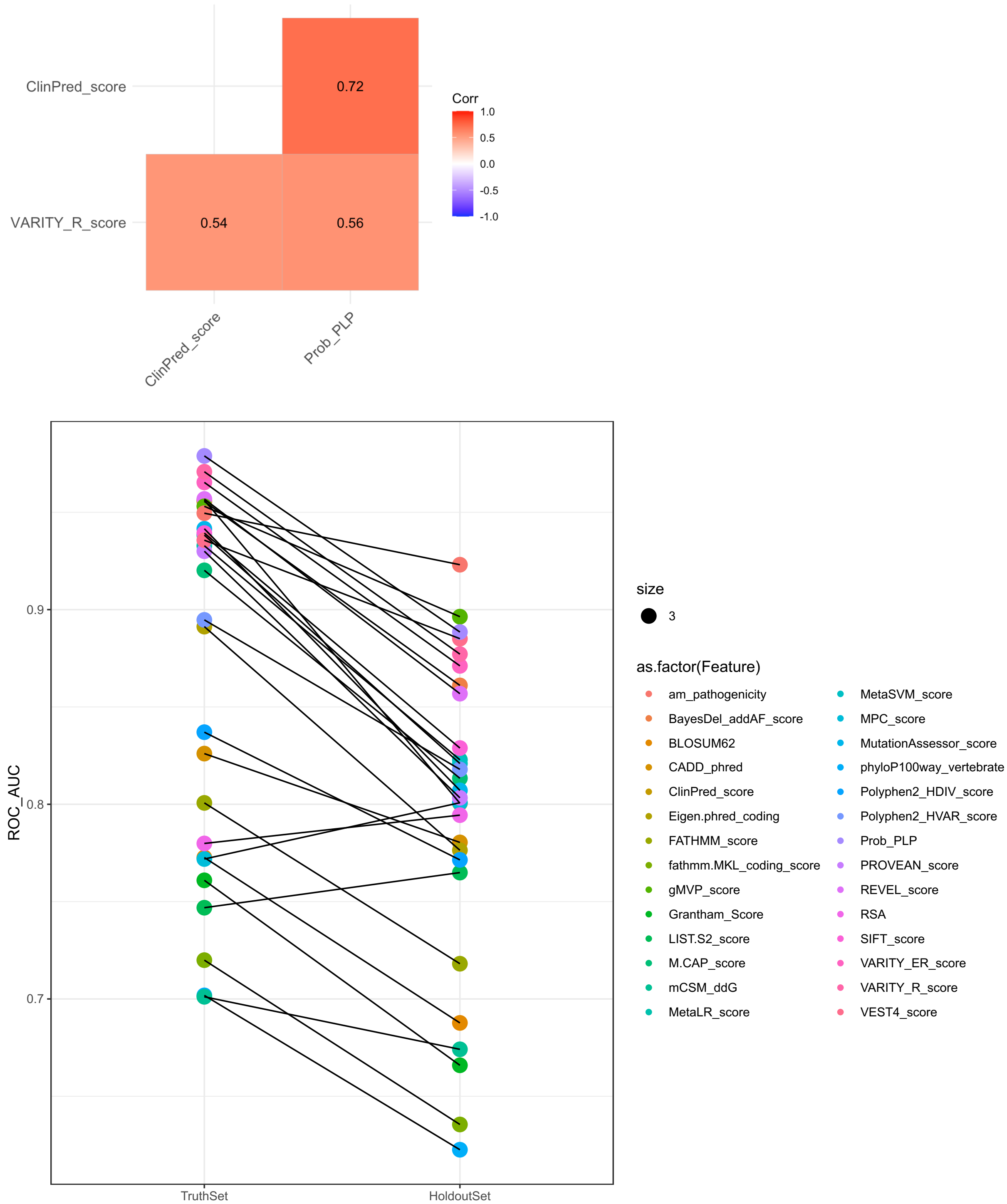

Supplementary Figure 13: Most VEPs/features have better classification performance on truth set as compared to holdout set.

ROC_{AUC} shown below, but also true for other model metrics. Filtered for only features/models with at least ROC_{AUC} of 0.7.

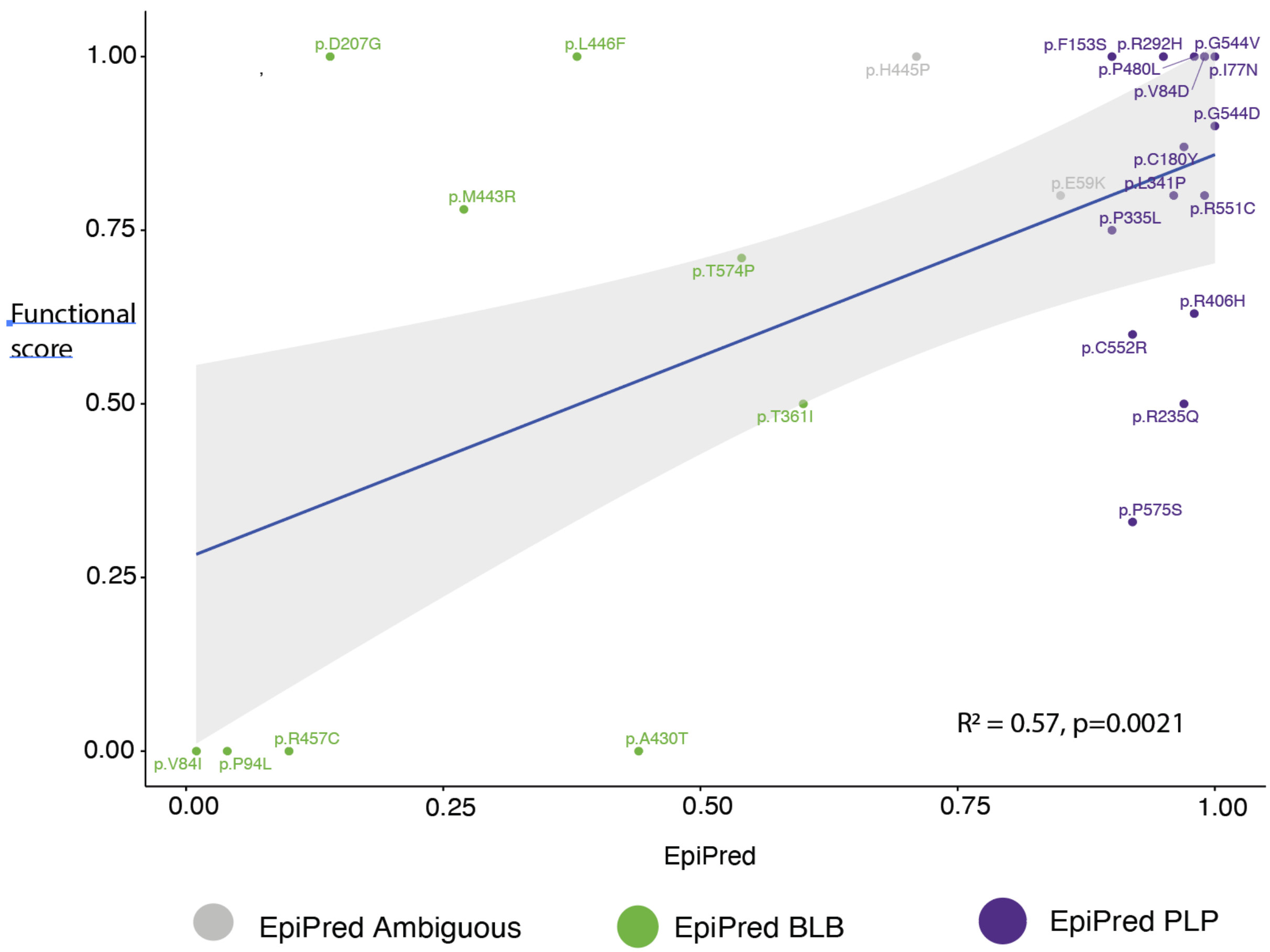

Supplementary Figure 14: An aggregate functional score from multiple publications, and correlation with EpiPred-PPLP scores. (r=0.57; p=0.0021 Pearson).

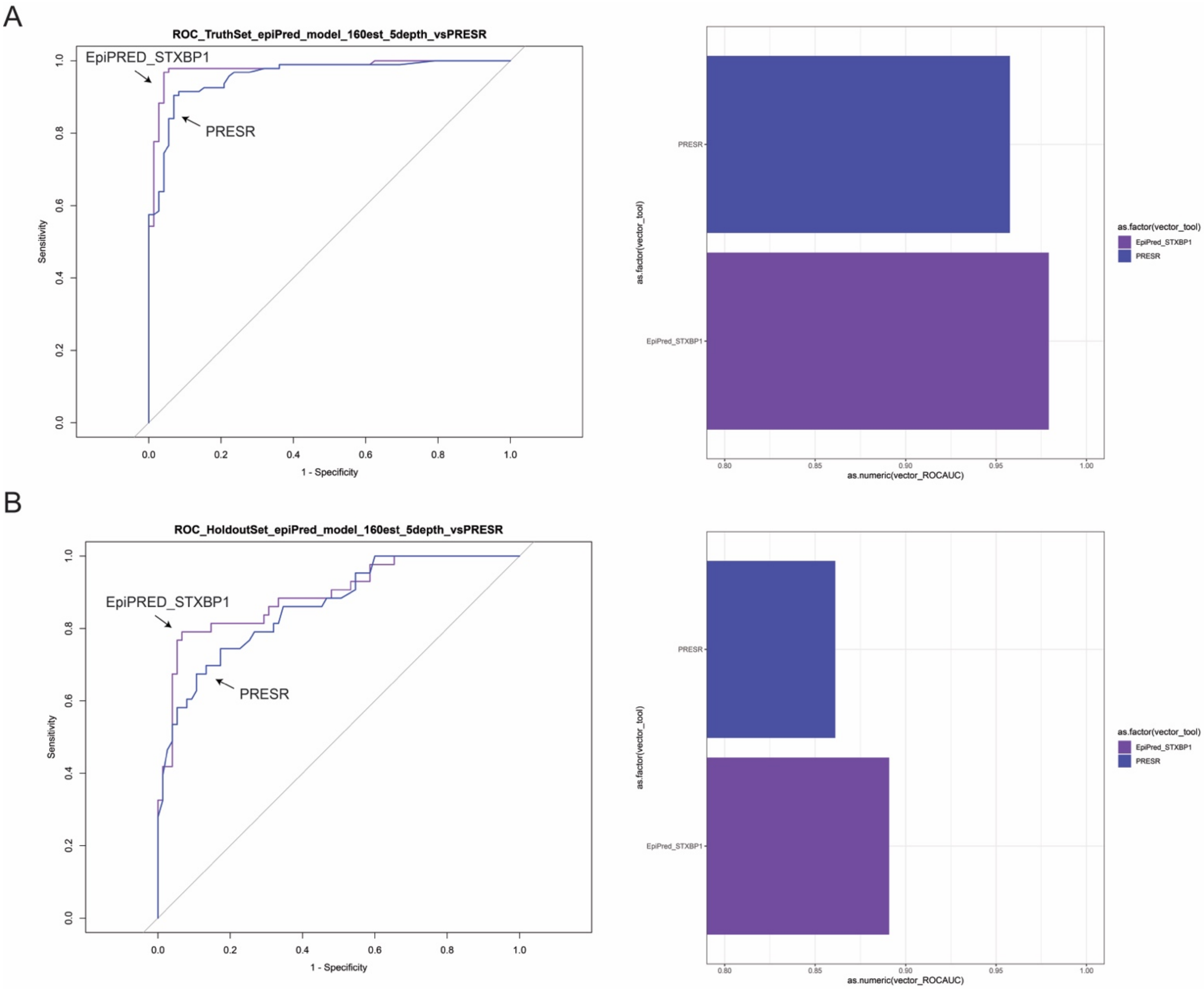

Supplementary Figure 15: ROC curve and ROC_{AUC} for EpiPRED compared with PRESR. Plots shown for full truth set (A) and the holdout dataset (B).

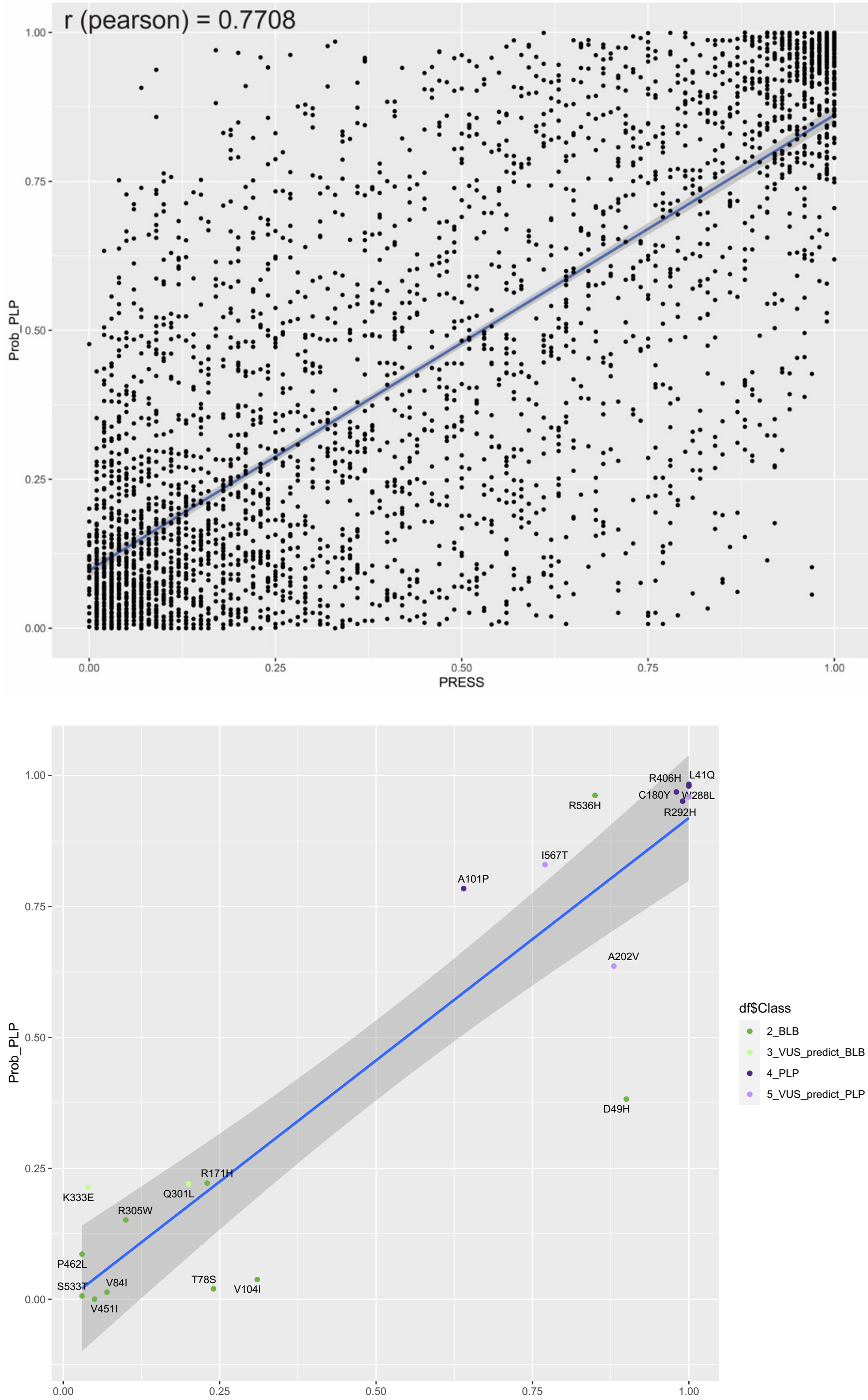

Supplementary Figure 16: PRESR and EpiPRED are highly correlated.
(Top) Scatterplot of EpiPred output (Prob_PLP) and PRESR output (PRESS) across the full EpiPred dataset including truth set, holdout set, VUS, and simulation variants. (Bottom) Scatterplot for a subset of variants with functional data (n=20) shown.
