# Supplementary material for: Gene-specific machine learning model EpiPred identifies likely pathogenic variants in the epilepsy-related gene STXBP1
Source: J Clin Invest. 2026 Sep 17;136(21):e207160. doi: 10.1172/JCI207160 (PMC13722379; doi:10.1172/JCI207160)
Supplement: Supplementary file 2 [file jci-136-207160-s169.pdf]

__Unedited western blots__

Set 1: Functional characterization assay for STXBP1: protein abundance assay.
Cells were transfected with equal amounts of either WT or variant expression plasmids, and STXBP1 abundance was measured with an anti-STXBP1 antibody.

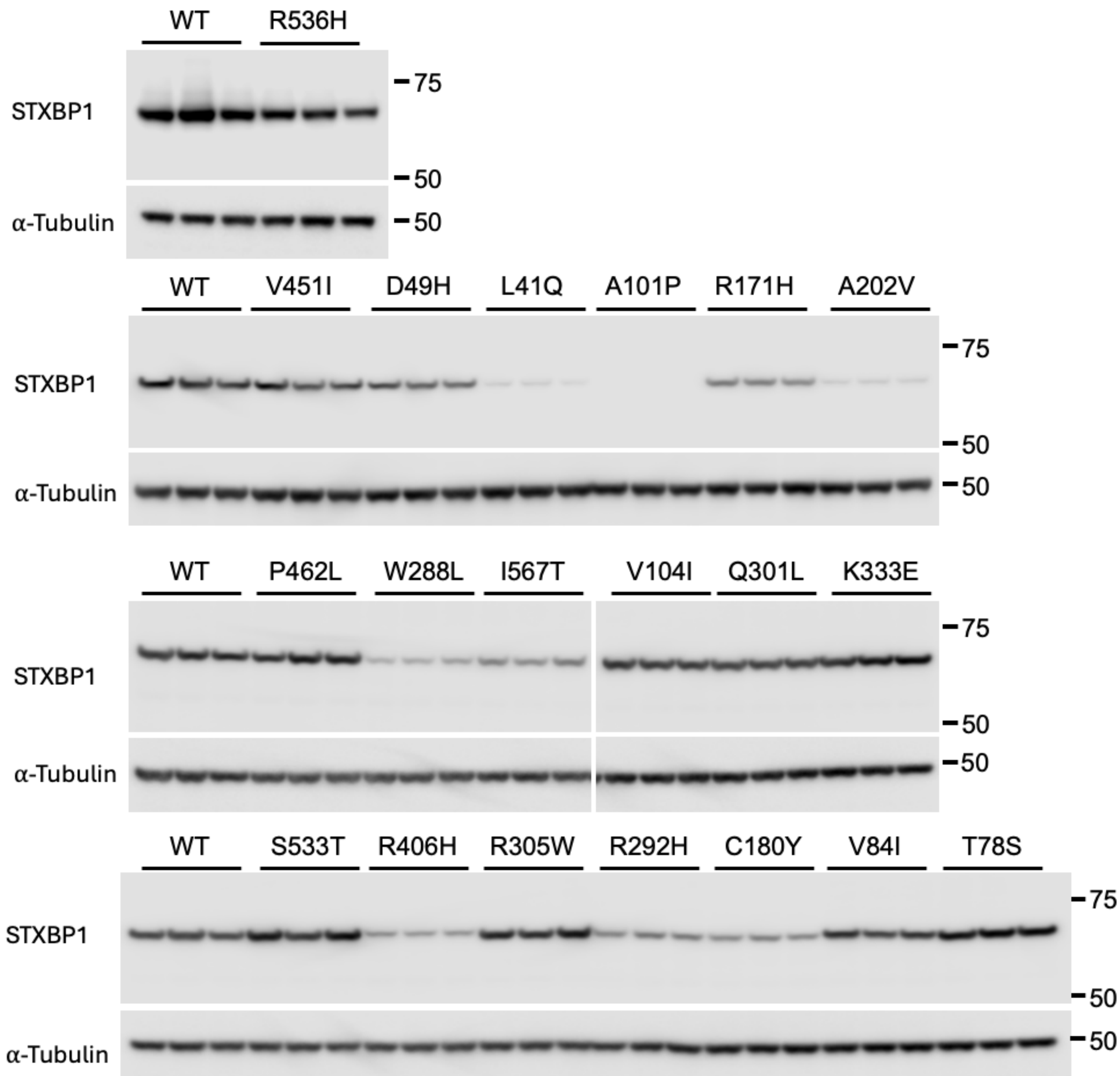

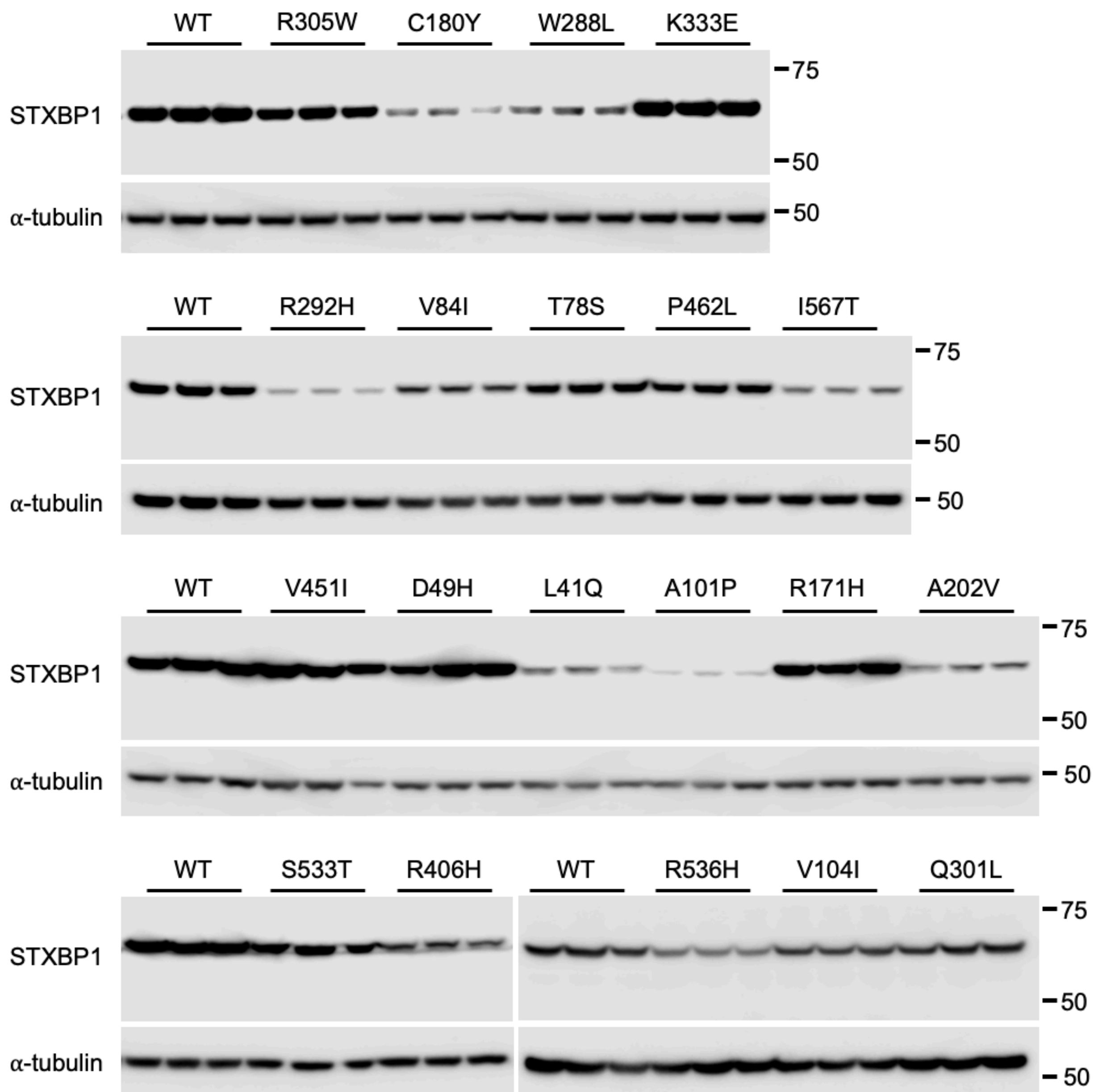
WT
R305W
C180Y
W288L
K333E
STXBP1
α-tubulin
75
50
50
WT
R292H
V84I
T78S
P462L
I567T
STXBP1
α-tubulin
75
50
50
WT
V451I
D49H
L41Q
A101P
R171H
A202V
STXBP1
α-tubulin
75
50
50
WT
S533T
R406H
WT
R536H
V104I
Q301L
STXBP1
α-tubulin
75
50
50

Set 2: Functional characterization assay for STXBP1: protein solubility assay.

Cells were transfected with equal amounts of either WT or variant expression plasmids, and insoluble and soluble fractions were collected. STXBP1 abundance was measured in each fraction. Revert 700 reagent was utilized to confirm loading of protein.

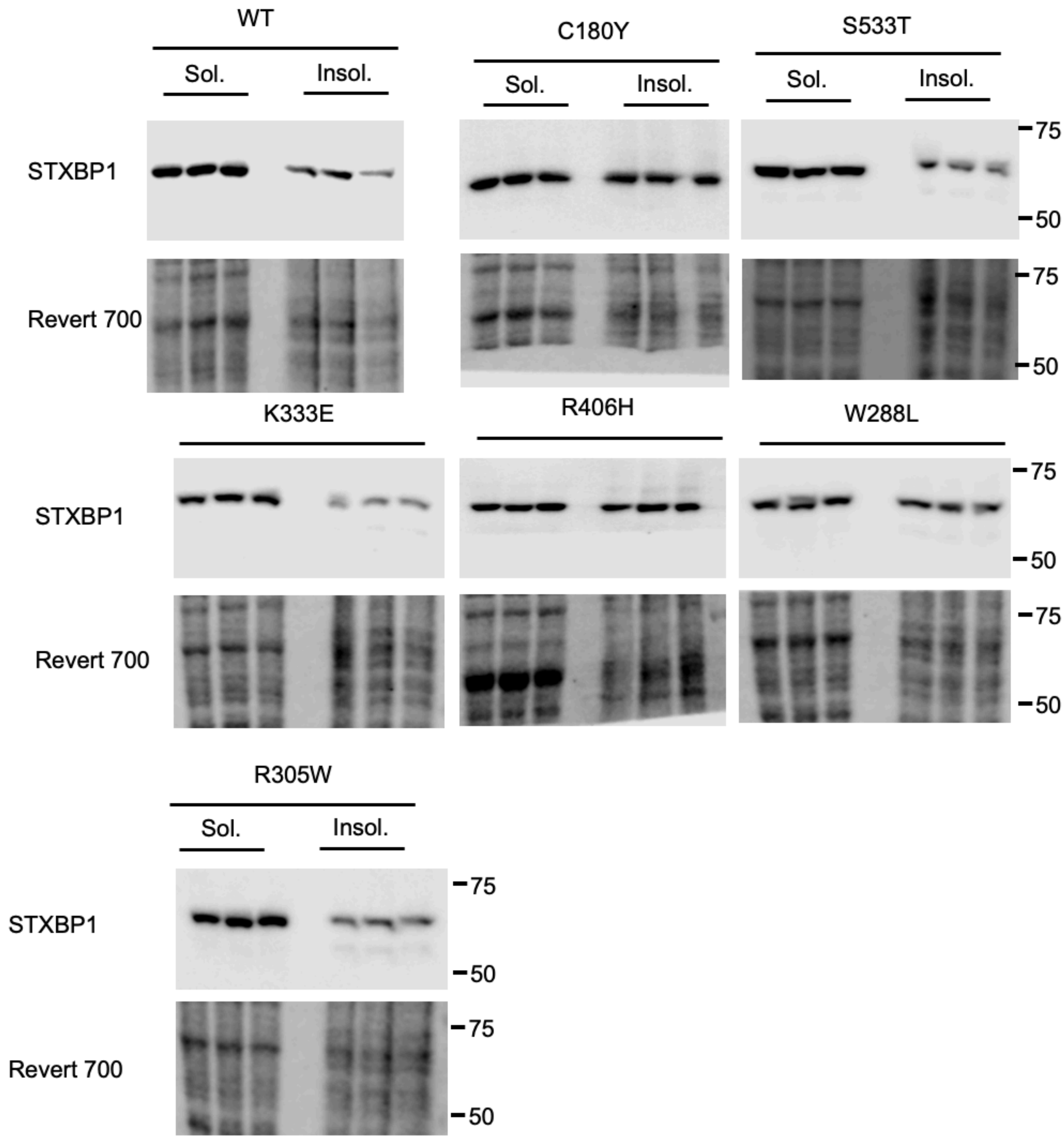

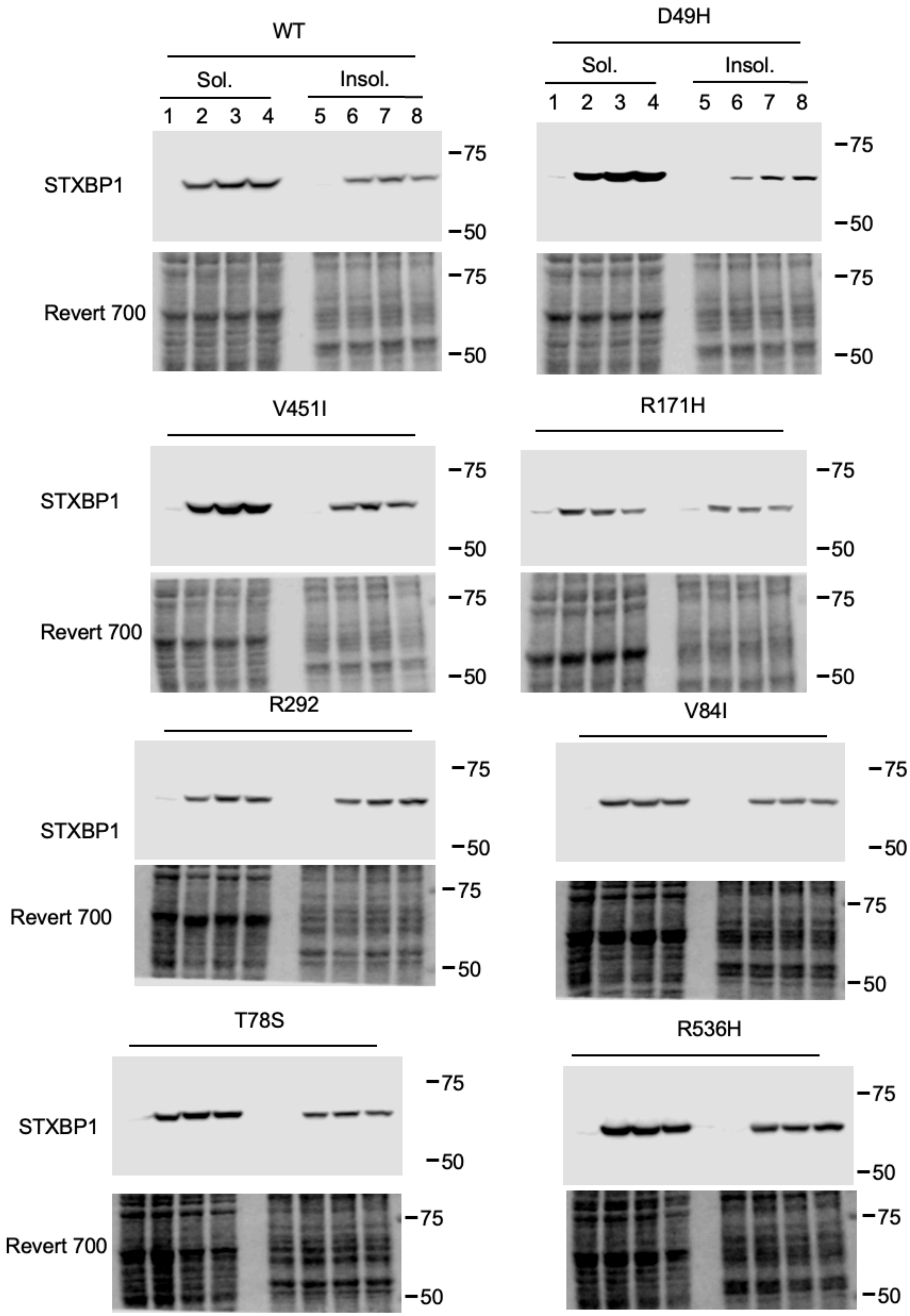

WT
Sol.
Insol.
1 2 3 4 5 6 7 8
STXBP1
Revert 700
D49H
Sol.
Insol.
1 2 3 4 5 6 7 8
STXBP1
Revert 700
V451I
STXBP1
Revert 700
R171H
R292
STXBP1
Revert 700
V84I
T78S
STXBP1
Revert 700
R536H
75
50

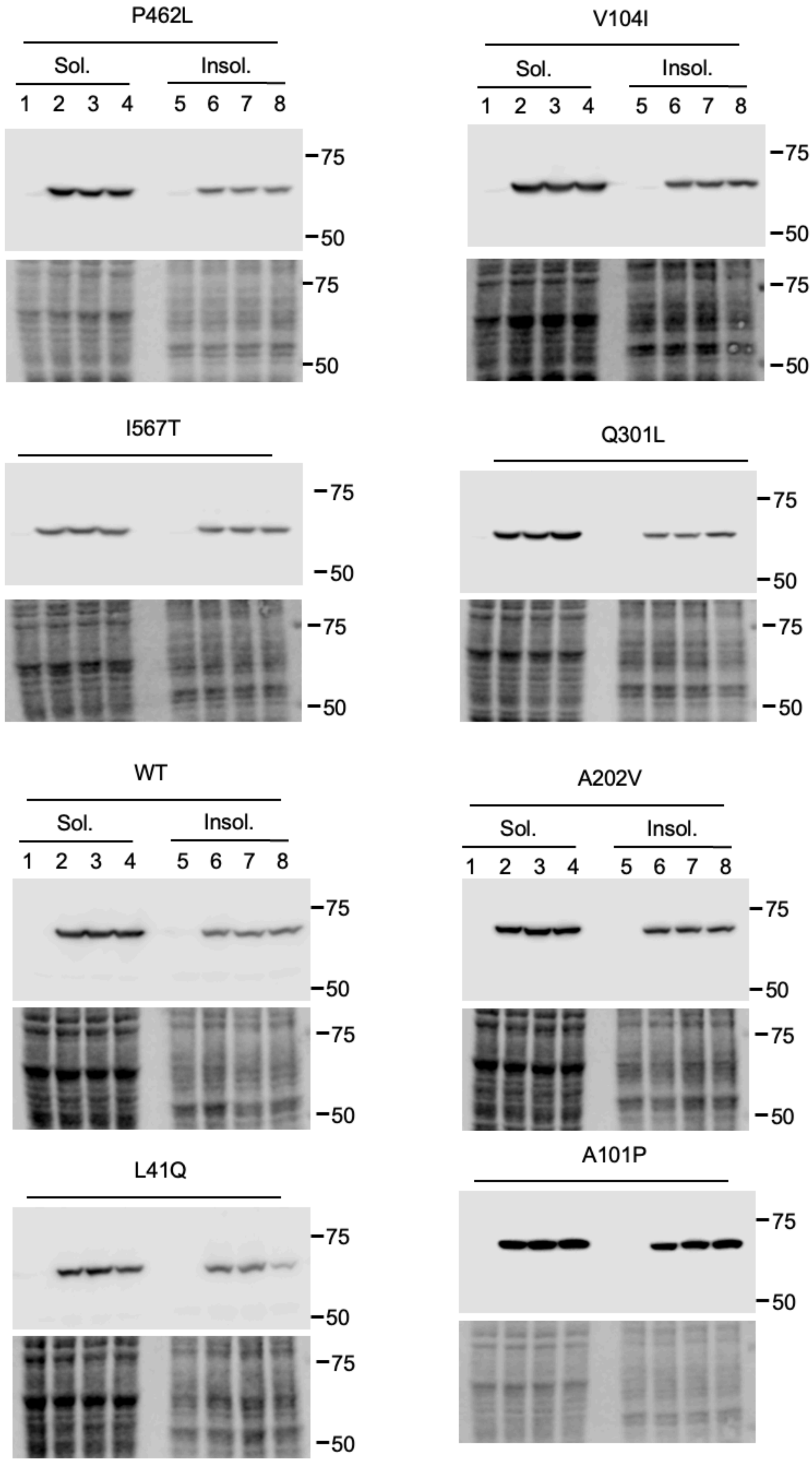
P462L
Sol.
Insol.
1 2 3 4 5 6 7 8
75
50
75
50
V104I
Sol.
Insol.
1 2 3 4 5 6 7 8
75
50
75
50
I567T
75
50
75
50
Q301L
75
50
75
50
WT
Sol.
Insol.
1 2 3 4 5 6 7 8
75
50
75
50
A202V
Sol.
Insol.
1 2 3 4 5 6 7 8
75
50
75
50
L41Q
75
50
75
50
A101P
75
50

Set 3: Functional characterization assay for STXBP1: protein stability assay.

Cells were transfected with equal amounts of either WT or variant expression plasmids. Cells were treated with either vehicle (DMSO) or cycloheximide (CHX) for 8 hrs prior to collection of total cell lysate, and STXBP1 abundance was measured in each treatment condition. Revert 700 reagent was utilized to confirm loading of protein.

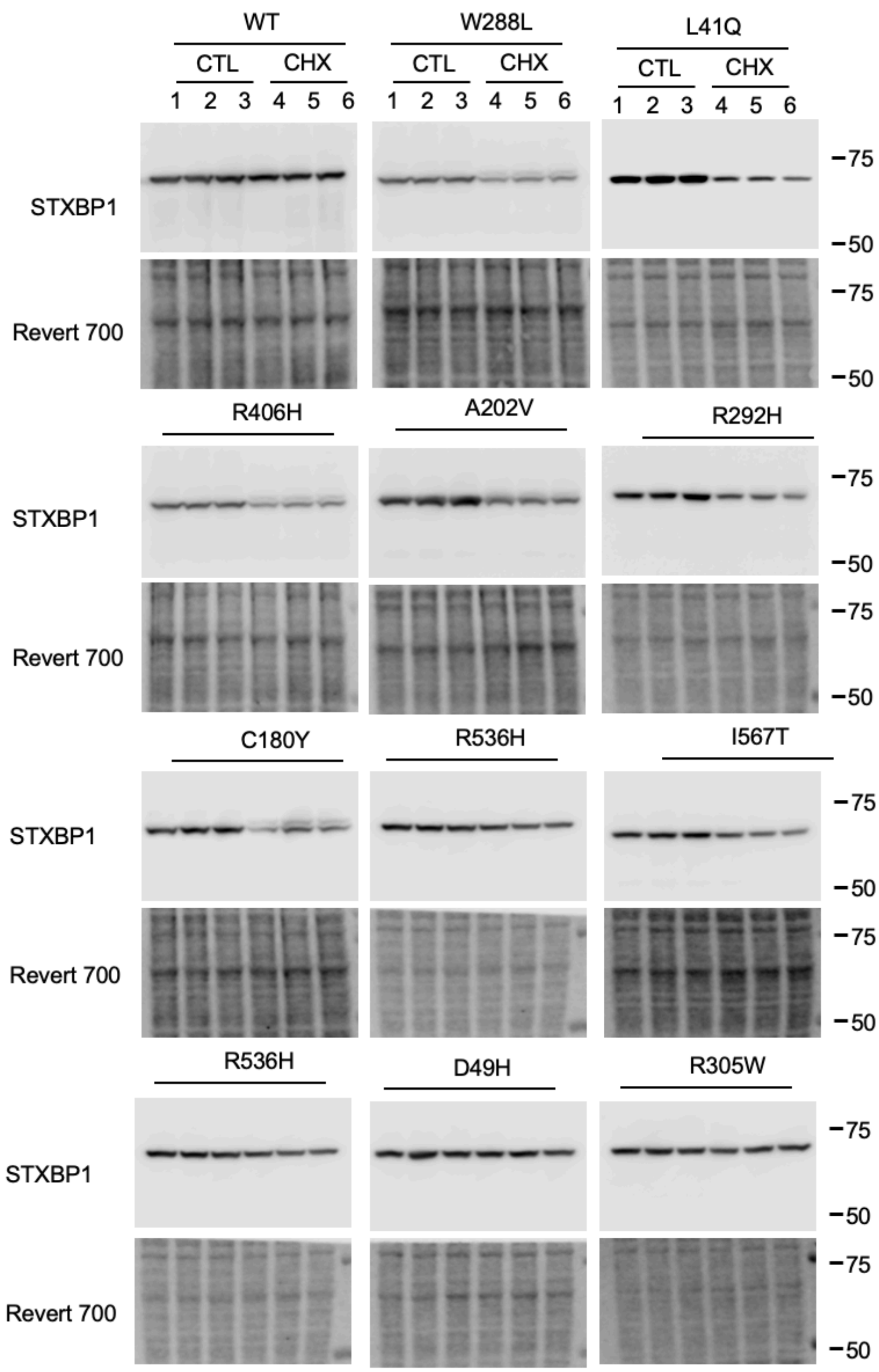

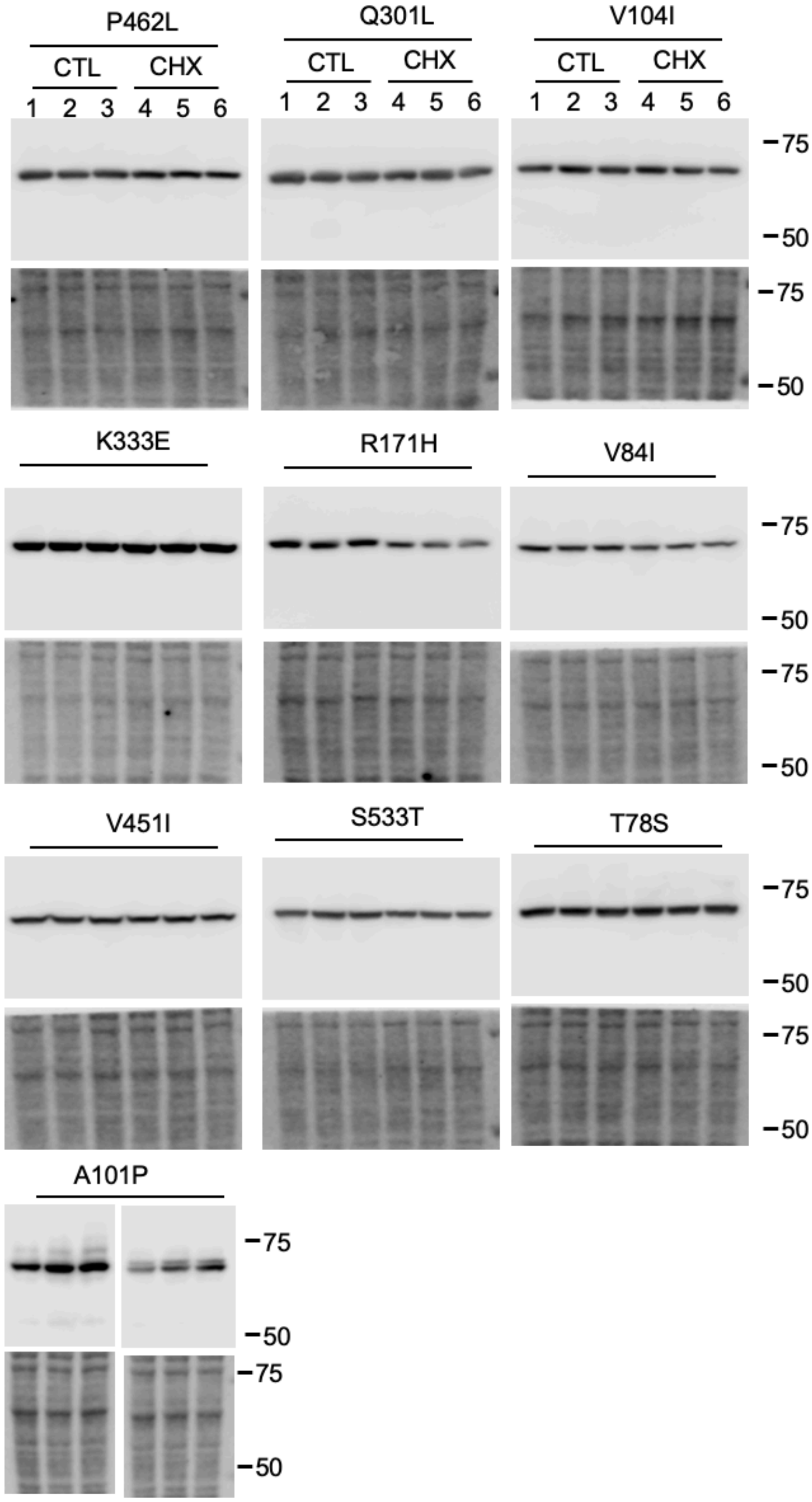
P462L
CTL
CHX
1 2 3 4 5 6
Q301L
CTL
CHX
1 2 3 4 5 6
V104I
CTL
CHX
1 2 3 4 5 6
75
50
75
50
K333E
R171H
V84I
75
50
75
50
V451I
S533T
T78S
75
50
75
50
A101P
75
50
75
50

Set 4 Functional characterization assay for STXBP1 interaction with STX1.

TRE-T-SNARE cells in 6xwell plates were transfected with different amounts of STXBP1 plasmid DNA to ensure a similar protein expression. Additionally, cells were co-transfected with equal amounts of an STX1 expression plasmid. Interaction was assessed by immunoprecipitation (IP) of STXBP1 followed by immunoblotting (IB) for S TX1.

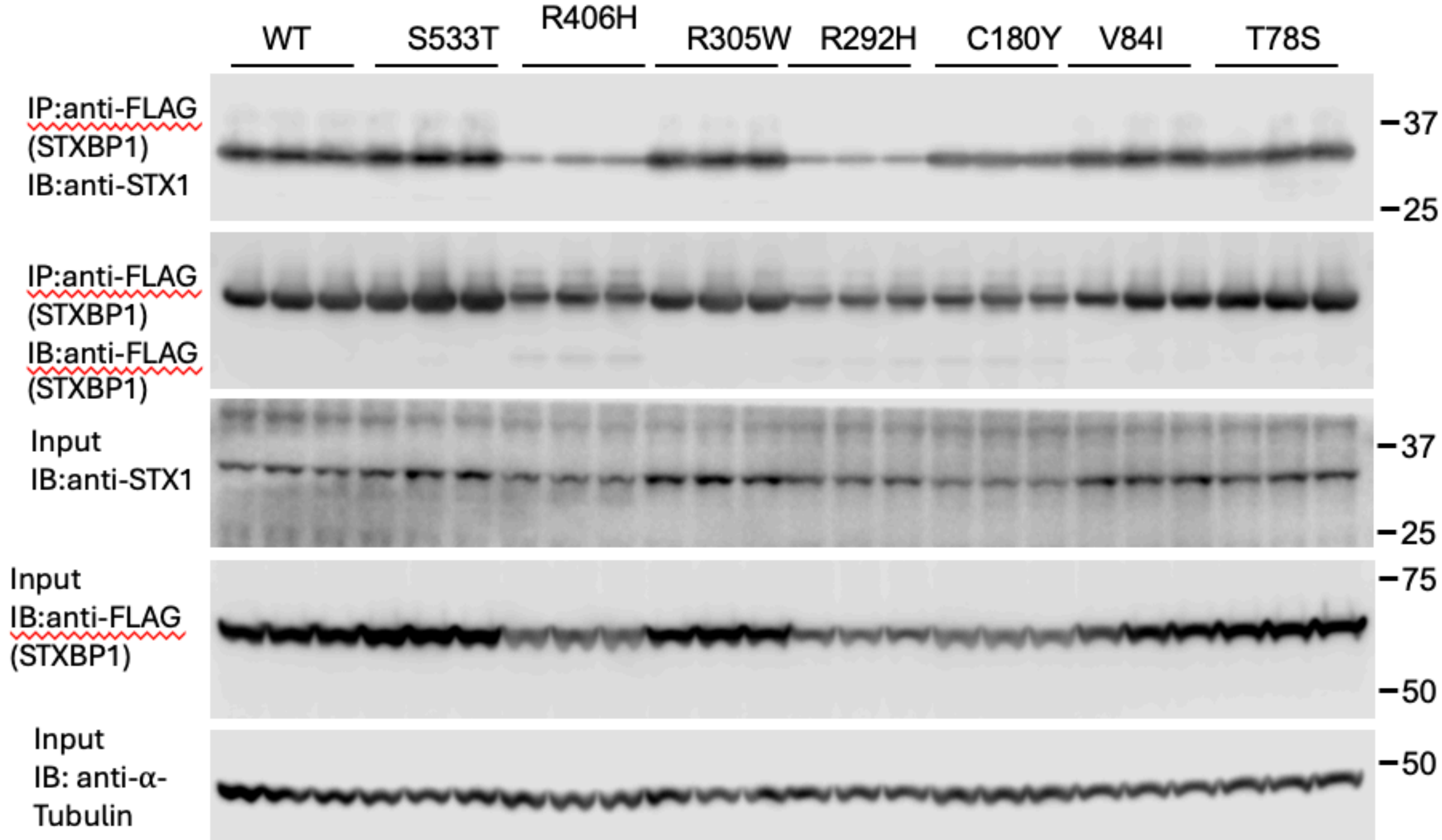

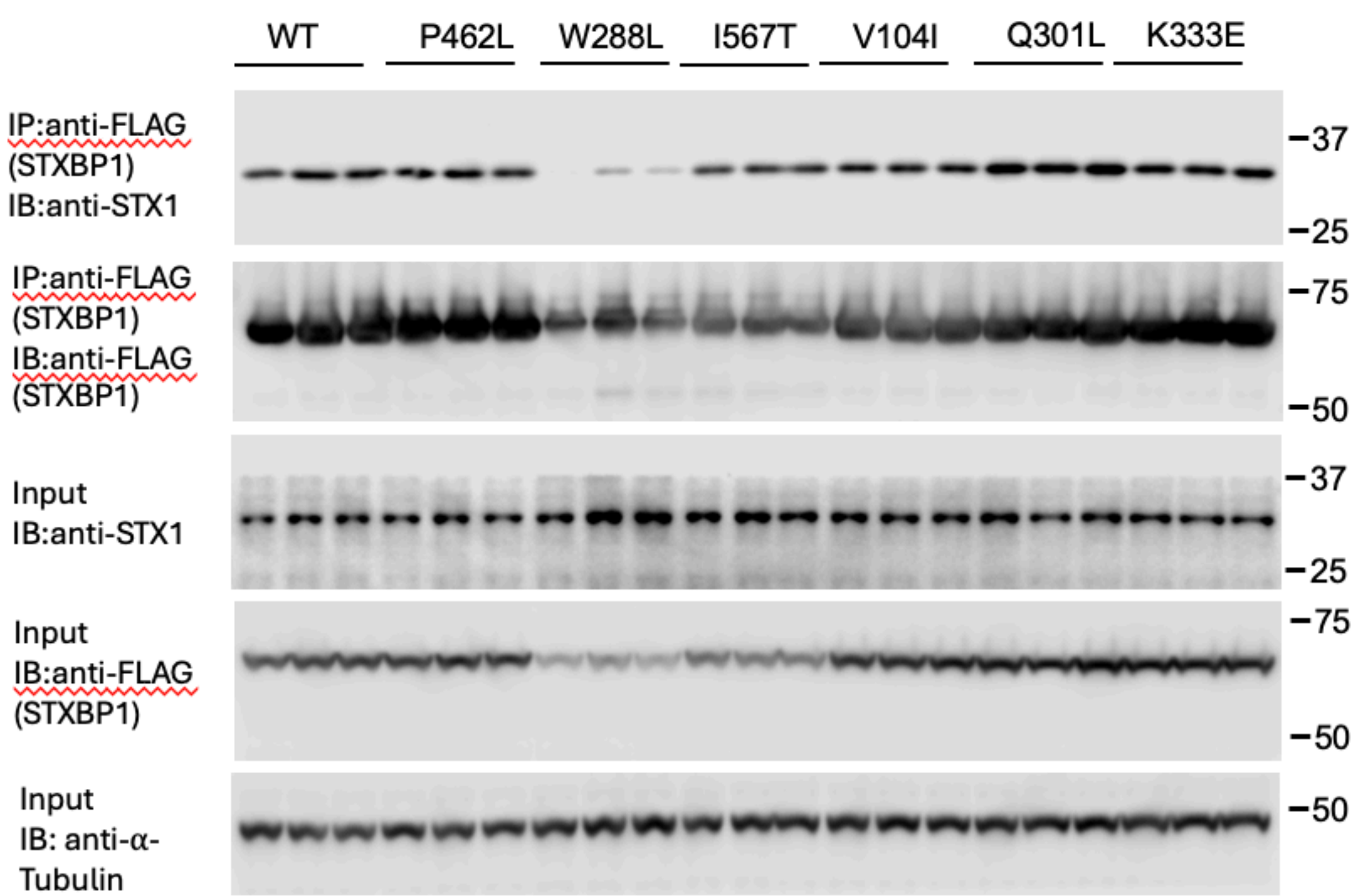

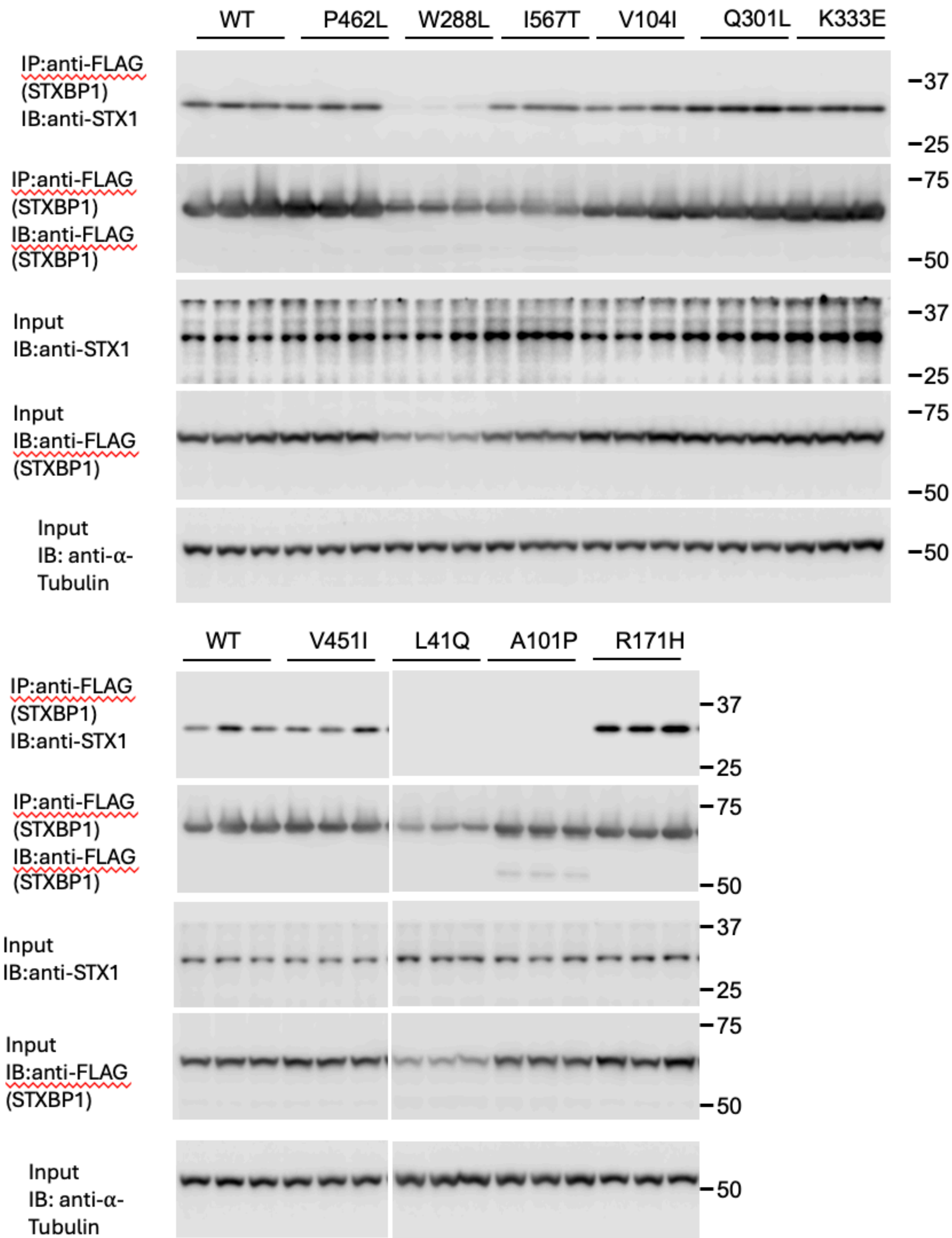
WT
P462L
W288L
I567T
V104I
Q301L
K333E
IP:anti-FLAG (STXBP1) IB:anti-STX1
IP:anti-FLAG (STXBP1) IB:anti-FLAG (STXBP1)
Input IB:anti-STX1
Input IB:anti-FLAG (STXBP1)
Input IB: anti-α-Tubulin
37
25
75
50
WT
V451I
L41Q
A101P
R171H

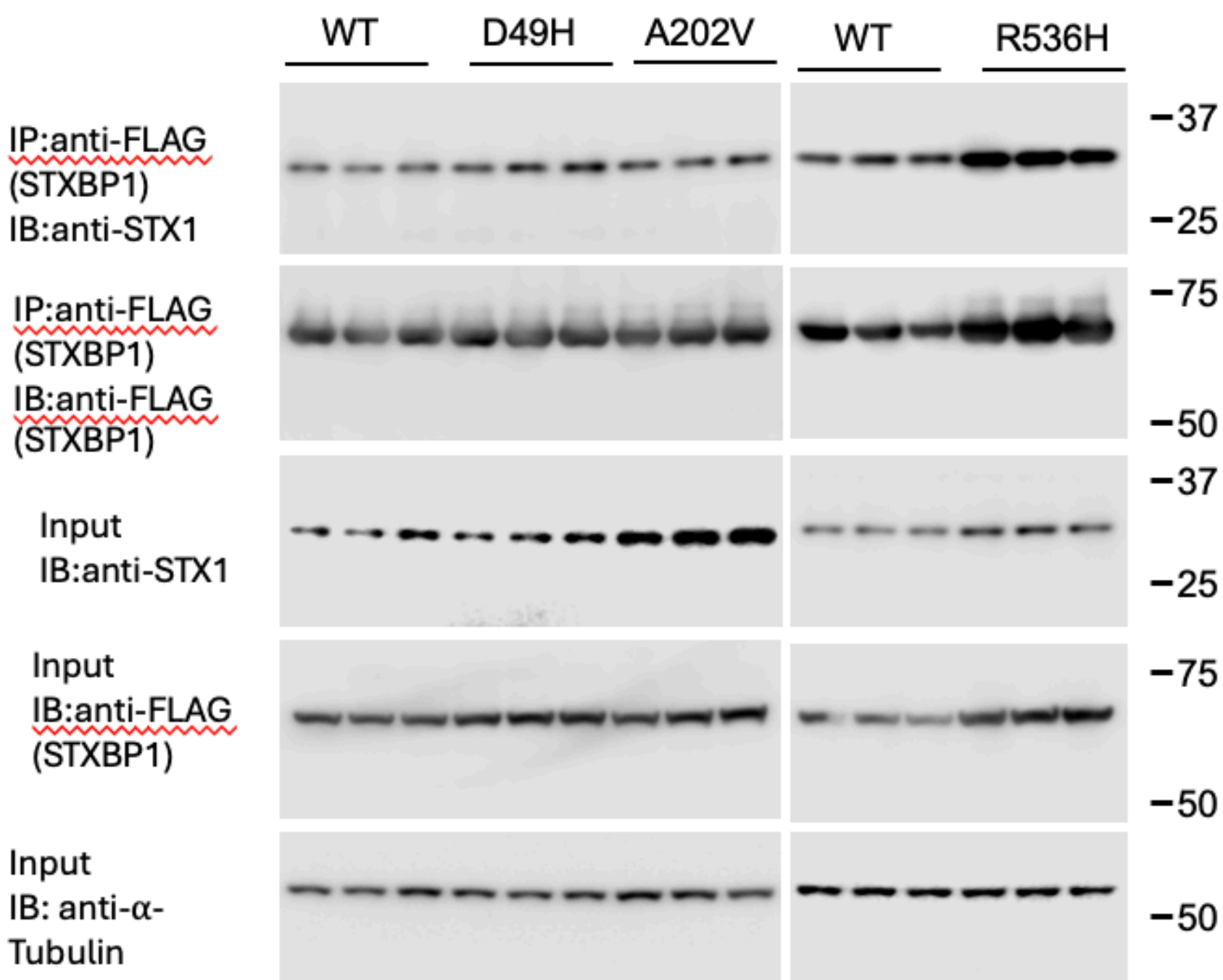
WT
D49H
A202V
WT
R536H
IP:anti-FLAG (STXBP1) IB:anti-STX1
IP:anti-FLAG (STXBP1) IB:anti-FLAG (STXBP1)
Input IB:anti-STX1
Input IB:anti-FLAG (STXBP1)
Input IB: anti-α-Tubulin
37
25
75
50
37
25
75
50
50
